# A Cell‐Permeable Photosensitizer for Selective Proximity Labeling and Crosslinking of Aggregated Proteome

**DOI:** 10.1002/advs.202306950

**Published:** 2024-03-05

**Authors:** Huan Feng, Qun Zhao, Nan Zhao, Zhen Liang, Yanan Huang, Xin Zhang, Lihua Zhang, Yu Liu

**Affiliations:** ^1^ State Key Laboratory of Medical Proteomics National Chromatographic R. & A. Center CAS Key Laboratory of Separation Science for Analytical Chemistry Dalian Institute of Chemical Physics Chinese Academy of Sciences 457 Zhongshan Road Dalian 116023 China; ^2^ University of Chinese Academy of Sciences Beijing 100049 China; ^3^ Department of Chemistry and Westlake Laboratory of Life Sciences and Biomedicine, Westlake University 18 Shilongshan Road Hangzhou 310024 China

**Keywords:** chaperone proteins, fluorescent probes, photoaffinity labeling, photosensitizers, proteomics

## Abstract

Intracellular proteome aggregation is a ubiquitous disease hallmark with its composition associated with pathogenicity. Herein, this work reports on a cell‐permeable photosensitizer (P8, Rose Bengal derivative) for selective photo induced proximity labeling and crosslinking of cellular aggregated proteome. Rose Bengal is identified out of common photosensitizer scaffolds for its unique intrinsic binding affinity to various protein aggregates driven by the hydrophobic effect. Further acetylation permeabilizes Rose Bengal to selectively image, label, and crosslink aggregated proteome in live stressed cells. A combination of photo‐chemical, tandem mass spectrometry, and protein biochemistry characterizations reveals the complexity in photosensitizing pathways (both Type I & II), modification sites and labeling mechanisms. The diverse labeling sites and reaction types result in highly effective enrichment and identification of aggregated proteome. Finally, aggregated proteomics and interaction analyses thereby reveal extensive entangling of proteostasis network components mediated by HSP70 chaperone (HSPA1B) and active participation of autophagy pathway in combating proteasome inhibition. Overall, this work exemplifies the first photo induced proximity labeling and crosslinking method (namely AggID) to profile intracellular aggregated proteome and analyze its interactions.

## Graphical abstract

A cell‐permeable photosensitizer is discovered to selectively bind to intracellular aggregated proteins and perform photo‐catalytic proximity labeling and crosslinking. Via both type I and type II reactive oxygen species, the photosensitizer enables the in situ profiling of the composition and interaction of cellular aggregated proteome.

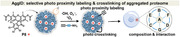

## 1 Introduction

Proteins perform their physiological functions when they fold into well‐defined three‐dimensional structures.^[^ ^1^ ^]^ However, they easily misfold, accumulate, and finally misassemble to insoluble aggregates upon mutations and prolonged stresses.^[^ ^2^ ^]^ The protein homeostasis (proteostasis) network, comprising protein biogenesis machineries, molecular chaperones and degradation systems (ubiquitin‐proteasome system, lysosome, autophagy, etc.), governs both the proper folding and clearance of proteins.^[^ ^3^ ^]^ Aberrant proteome stress and consequent proteome aggregation, resulting from imbalanced proteostasis, is a ubiquitous hallmark of various diseases, such as neurodegeneration, metabolic disorders, and certain types of cancers.^[^ ^4^ ^]^ Reliable identification of intracellular aggregated proteome is highly desired when exploring the underlying biological mechanisms and developing therapeutic approaches for these diseases.

Much light has been shed on the development of imaging sensors to visualize the location and morphology of misfolded and aggregated proteome.^[^ ^5^ ^]^ Hong and Hatters et al. pioneered in the application of covalent fluorescent probes to detect unfolded proteome in stressed live cells.^[^ ^6^ ^]^ Liu et al. tracked the dynamics of amorphous aggregated proteome using fluorescent protein chromophore analogues.^[^ ^7^ ^]^ Sensors of diverse signal readouts have been developed for in vivo mapping of amyloid‐*β* plaques and Tau tangles.^[^ ^8^ ^]^ However, very limited chemical tools (Figure S1, Supporting Information) are available to report on the composition inside aggregated proteome, an essential piece of information intimately associated with disease pathogenicity.

Proximity labeling technology (PLT) in combination with chemical proteomics analysis flourishes in the past decade given its indispensable role in resolving biological components with spatio‐temporal resolution. This method has been applied to profile spatiotemporal transcriptome, sub‐organellar proteome and transient protein interactome.^[^ ^9^ ^]^ PLT commonly employs enzyme tags (APEX, biotin ligase, miniSOG, et al.) or photocatalysts (Ru complex, dibromofluorescein, flavin, µMAP, et al.) to perform its in situ labeling functions (Figure S2, Supporting Information).^[^ ^10^ ^]^ Mediated by genetic encoding or antibody targeting, selective proximity labeling of a protein‐of‐interest (POI)’s interactome can be achieved via sets of novel photo‐catalytic reactions.^[^ ^11^ ^]^ Inspired by these seminal works, we envision that a small molecule guided PLT may be realized to profile aggregated proteome in live cells if the chemical probe bears the following properties: 1) selective & general binding to various protein aggregates; 2) effective photosensitized oxidation of protein residues for afterward covalent labeling and enrichment; 3) photo crosslinking function to stabilize the easily dissociated intracellular aggresome complex.

**Figure 1:**
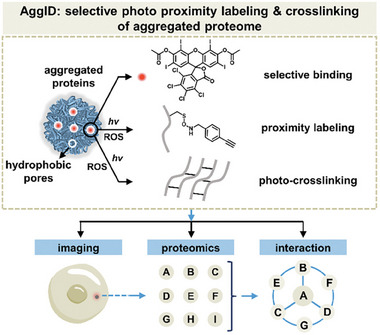
Discovery of a small molecule photosensitizer enables proximity labeling and crosslinking of aggregated proteome in live cells to profile its composition and interaction network.

Herein, we reported that xanthene‐based Rose Bengal (RB, P5) can selectively and generally bind to aggregated proteins after searching out of a collection of organic photosensitizer scaffolds (**Figure** **1**; Figure S2, Supporting Information). The selective binding affinity was governed by probes’ hydrophobicity (cLogP). Such strong binding affinity combined with intrinsic triplet photo‐oxidation function enabled proximity labeling and crosslinking of aggregated proteins. Photochemical analysis revealed that both type I and type II reactive oxygen species (ROS) contributed to the effective labeling via nucleophilic and radical reactions. Using tandem mass spectrometry, we identified diverse labeling sites at different amino acid side chains via various reaction mechanisms for expanded labeling coverage compared to the classic miniSOG enzyme. Cell permeabilization of P5 through acetylation allowed for selective imaging, labeling, crosslinking, and enrichment of aggregated proteome in live HeLa cells stressed by proteasome inhibitor drug Bortezomib. Aggregated proteomics and interactions analysis thereby uncovered that key components of autophagy pathway along with other proteostasis network players was sequestered in cellular aggresome. Notably, the most extensively labeled hub protein was identified as HSPA1B (a HSP70 chaperone), suggesting its central role in networking and chaperoning the formation of intracellular aggresome upon failure of proteasome function. To the best of our knowledge, the reported method (AggID) is the first photo proximity labeling and crosslinking tool to profile cellular aggregated proteome.

## 2 Results and Discussion

### 2.1 Searching for Selective Binder of Aggregated Proteins from the Fluorophore and Photosensitizer Pool

**Figure 2:**
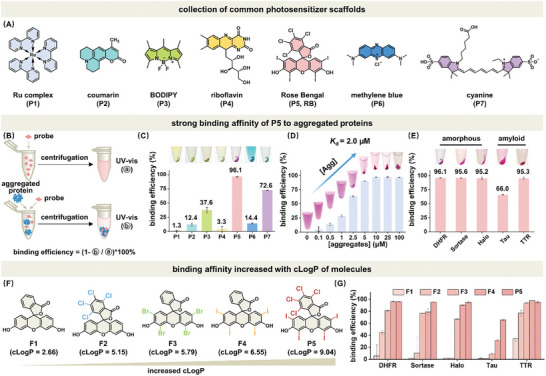
Searching for a selective binder of aggregated proteins from common fluorophore and photosensitizer scaffolds. A) Common fluorescent dyes (coumarin, BODIPY, methylene blue, and cyanine) and photosensitizers (Ru complex, riboflavin, and Rose Bengal) scaffolds. B) Assessing binding efficiency of probes to aggregated proteins by measuring the absorption partition of probes into insoluble fraction through centrifugation fractionation. C) P5 outperformed other probes in binding efficiency (up to 96.1%) toward aggregated DHFR protein. D) P5 gradually partitioned into increasing amount of aggregated DHFR (0.0–100.0 µm). *K*_d_ of P5 toward aggregated DHFR was determined as 2.0 µm. E) P5 generally bound to different aggregated proteins (both amorphous and amyloid) at 20.0 µm. F) Structures of xanthene family fluorophores and photosensitizers with increasing cLogP. G) The binding affinity of probes to aggregated proteins increased with cLogP across all tested types of aggregated proteins. Unless otherwise noted, protein concentration used in these experiments was 100.0 µm except TTR (25.0 µm). Probe concentration used in these experiments was 20.0 µm. Error bars: standard error (*n* = 3).

In this part, we aim to search for a selective binder that is generally applicable to different types of aggregated proteins. As noted in the introduction, the prerequisites for small molecule mediated photo proximity labeling and crosslinking of aggregated proteome are 1) probe's selective binding affinity to different types of protein aggregates; 2) photosensitized modifications of protein residues. To this end, we embarked on searching for an ideal probe from a collection pool of common fluorophore and photosensitizer scaffolds, including Ru complex (P1), coumarin (P2), BODIPY (P3), riboflavin (P4), Rose Bengal (P5), methylene blue (P6), and cyanine (P7) (**Figure** **2**A). Using *Escherichia coli* dihydrofolate reductase (DHFR) as a model protein that formed amorphous aggregates upon heating, we quantified the normalized binding efficiency of these probes to protein aggregates by measuring the absorption partition of probes into insoluble fraction through centrifugation fractionation (Figure 2B). We observed that up to 96.1% of P5 bound to aggregated DHFR, outperforming the other compounds (Figure 2C). Such strong binding affinity retained above 80% even after 5 times PBS washing of aggregates (Figure S3, Supporting Information). We determined the dissociation constant (*K*_d_) of P5 to be 2.0 µm by analyzing its binding efficiency at different concentrations of protein aggregates (Figure 2D, Equation (1)). We next interrogated the binding selectivity of P5 to aggregated proteins over their folded counterparts (Figure S4, Supporting Information). Importantly, the selective binding affinity of P5 to protein aggregates was generally applicable to other protein types (amorphous aggregated proteins: sortase, HaloTag; amyloid aggregated proteins: Tau‐K18, transthyretin (TTR), Figure 2E) as well as cell lysates (Figure S5a,b, Supporting Information 98.6% partitioned in aggregates). Such high binding efficiency in aggregated cell lysate retained (88.5%) even in the presence of folded lysate (Figure S5c,d, Supporting Information). However, the concern about non‐specific binding (11.5%) to folded counterparts can be addressed by background correction in downstream proteomics analysis.

We hypothesized the hydrophobic effect during protein aggregation may contribute to P5's preferential binding affinity toward aggregated proteins over folded ones, given the fact that protein misfolding and aggregation often involves the exposure of hydrophobic residues.^[^ ^12^ ^]^ To validate the origin of such selective binding, we uncovered that the increasing binding efficiency of P1 to P7 to all tested types of protein aggregates was positively correlated to probes’ cLogP values (Table S1, Supporting Information). Such observation was further supported by the positive correlation between binding efficiency to protein aggregates and cLogP values within xanthene family (Figure 2F,G). However, one may pay attention to the non‐specific binding of the probe to the hydrophobic region of folded counterparts, raising potential background false‐positive issue (Figure S5c,d, Supporting Information). Overall, we discovered out of the common fluorophore and photosensitizer pool that P5 (Rose Bengal) was a selective and strong binder for different types of amorphous and amyloid aggregated proteins, which satisfied the first prerequisite to develop this tool.

### 2.2 Photo Induced Proximity Labeling and Crosslinking of Aggregated Proteins Using P5

**Figure 3:**
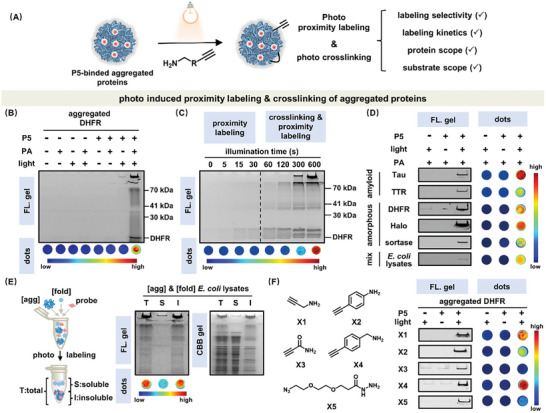
Photo induced proximity labeling and crosslinking of aggregated proteins using P5. A) Photo induced labeling and crosslinking of aggregated proteins by P5 were analyzed in terms of labeling selectiveity, kinetics, protein, and substrate scope. B) P5 covalently modified aggregated DHFR in proximity using propargylamine as the labeling substrate. C) Time course of photo proximity labeling within 30 s and photo crosslinking afterward for aggregated DHFR. [P5]: 2.0 µm; Light intensity: 3 mW·cm^−2^. D) P5 generally proximity labeled and crosslinked different types of aggregated proteins and lysate upon photo illumination. E) P5 selectively photo proximity labeled and crosslinked aggregated *E. coli* lysate over folded one. F) Benzylamine substrate offered the highest labeling efficiency for P5. Unless otherwise specified, these experiments were performed under following conditions: [protein]: 2.0 mg·mL^−1^; [P5]: 20.0 µm; [substrate]: 10.0 mm; Light intensity: 25 mW·cm^−2^; Illumination time: 20 min. Error bars: standard error (*n* = 3). Dots experiments were performed on nitrocellulose film and imaged using VISQUE InVivo Smart‐LF bio‐imaging system. The fluorescence signal of photo proximity labeling was from conjugation with TMR. All controlled coomassie brilliant blue (CBB) gel and quantification of dots experiment were shown in Figure S6 (Supporting Information) for (B), Figure S8 (Supporting Information) for (C), Figure S9 (Supporting Information) for (D), and Figure S13 (Supporting Information) for (F).

We next examined whether P5 can selectively proximity label and crosslink aggregated proteins upon photo‐illumination with focuses on labeling selectivity, kinetics, protein, and substrate scopes (**Figure** **3**A). Given the above‐demonstrated strong & selective binding affinity to aggregated proteins, as well as its intrinsic triplet photosensitizing properties, P5 in theory can label aggregated proteins in proximity upon photo illumination. We next examined the labeling efficiency by fluorescence SDS‐page gel and dots quantification assay designed for protein aggregates (Experimental sections). Previous works showed propargyl amine (PA) was a commonly employed substrate for photo induced proximity labeling and downstream enrichment.^[^ ^13^ ^]^ In line with our expectations, upon P5 mediated photo induced proximity labeling, protein aggregates were effectively labeled by propargyl amine and visualized by clicking with fluorescent tetramethylrhodamine (TMR) (Figure 3B; Figure S6, Supporting Information). Intriguingly, we also observed crosslinked products on the gel at higher molecular weights in addition to the monomeric aggregated protein, suggesting its potential in crosslinking and stabilizing the easily dissociable aggregated proteome in live cells (Figure 3B, lane 8). Furthermore, we confirmed that the proximity labeling efficiency depended on the concentration of P5, light intensity, and illumination time (Figure S7, Supporting Information). Notably, rapid proximity labeling of aggregated proteins was achieved within 30 s, and prolonged illumination led to effective crosslinking of the aggregated proteins (Figure 3C; Figure S8, Supporting Information). Such rapid proximity labeling aligned with desired crosslinking property, in principle, may assist the capture of the highly dynamic aggregated proteome and their interacting information. Importantly, in line with general binding (Figure 2E), the labeling and crosslinking was also applicable to various types of aggregated proteins, including amyloids (Tau‐K18 and TTR), amorphous aggregates (DHFR, HaloTag, and sortase), and cellular aggregates (lysates) (Figure 3D; Figure S9, Supporting Information).

The protein aggregation process is a multi‐step biochemical pathway that involves the conversion of folded proteins (*F*) to unfolded proteins (*U*), misfolded oligomers (*M*), and ultimately insoluble aggregates (*I*) (Figure S10, Supporting Information).^[^ ^14^ ^]^ In this part, we aimed to dissect at which stage P5 began to label proteins. Our results demonstrated that the labeling efficiency significantly increased as proteins misfolded into oligomeric state (Figure S10, Supporting Information). These results were further validated by monitoring the labeling efficiency of P5 using aggregated *E. coli* lysates proteome upon gradual misfolding and aggregation controlled by temperatures (Figure S11, Supporting Information). Moreover, the proximity labeling selectively occurred on aggregated *E. coli* lysates even in the presence of folded one (Figure 3E). We deduced that the confinement of P5 inside aggregated proteins together with the nature of the short‐lived ROS though limited the labeling range but ensured the labeling selectivity to aggregated proteins even in presence of folded counterpart (Figure S12, Supporting Information).

To optimize the labeling efficiency, we finally tested different types of various nucleophilic substrates (X1 to X5, aliphatic amine, aniline, amide, benzylamine, and hydrazide) (Figure 3F; Figure S13, Supporting Information). Benzylamine (X4) and aliphatic amine (X1) exhibited better photo induced proximity labeling of aggregated proteins, with X4 slightly surpassing X1. Due to its commercial availability and smaller size, X1 was selected as substrates for in‐cell applications afterward. Together, P5 can rapidly and selectively proximity label and crosslink aggregated proteins upon photo illumination.

### 2.3 Mechanistic Insights of Photo Induced Proximity Labeling Reactions Mediated by P5

**Figure 4:**
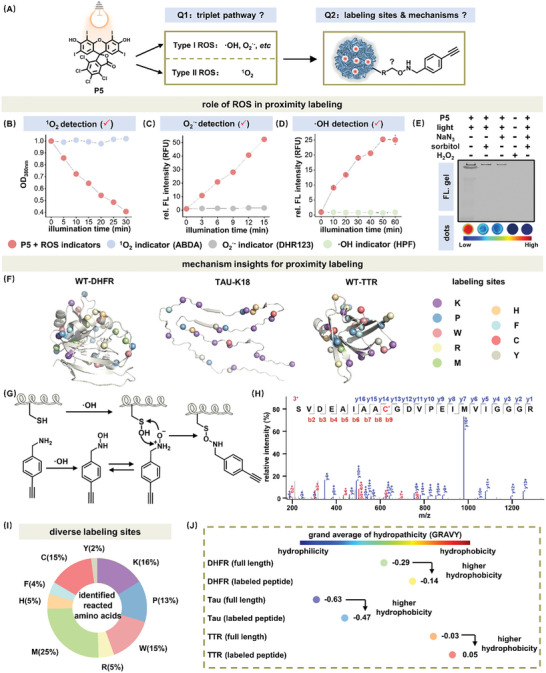
Mechanistic insights of P5 mediated photo induced proximity labeling reactions. A) Potential pathways that P5 underwent upon photo induced proximity labeling of aggregated proteins and how aggregated proteins were proximity labeled. B) Singlet oxygen generated by P5 (3.0 µm) was detected by ABDA (0.1 mm). Absorbance at 378 nm was collected. C) Superoxide anions generated by P5 (3.0 µm) was detected by DHR123 (6.0 µm, ex. 480 nm, em. 525 nm). D) Hydroxyl radicals generated by P5 (3.0 µm) was detected by HPF (3.0 µm, ex. 480 nm, em. 514 nm). E) The proximity labeling efficiency of aggregated proteins induced by P5 was quenched by type II singlet oxygen scavenger or type I ROS scavenger. [NaN_3_], [sorbitol] and [H_2_O_2_]: 10.0 mm. The controlled CBB gel and quantification of dots experiment were shown in Figure S14 (Supporting Information). F) Labeling sites of aggregated DHFR, Tau‐K18, and TTR that were highlighted on folded structures for illustration purpose (PDB: 1RX5, 6TJO, and 3KGU). G) Proposed reaction mechanism of benzylamine labeled cysteine and (H) its corresponding LC‐MS/MS spectra. I) Labeling preference of amino acids. J) The labeling sites of three model proteins tended to be more hydrophobic. The grand average of hydropathicity (GRAVY) was calculated using Expasy Protparam. The length of the calculated labeling peptide was nine amino acids. Experiments in Figure 4F–J were performed under following conditions: [protein]: 2.0 mg·mL^−1^; [P5]: 20.0 µm; [substrate]: 10.0 mm; Light intensity: 25 mW·cm^−2^; Illumination time: 30 min. All experiments were performed in triplicates. Error bars: standard error (*n* = 3).

It is essential to understand the photochemical mechanism underlying P5's proximity labeling process by addressing two questions: 1) the types of ROS; 2) the labeling sites and reaction types (**Figure** **4**A). To investigate the role of ROS species in P5's proximity labeling and crosslinking reaction, we first determined the photosensitizing pathway (type I for superoxide anions, hydroxyl radicals, etc. or type II for singlet oxygen) that P5 underwent upon photo illumination. Intriguingly, our assays demonstrated that P5 generated both type I ROS (superoxide anions and hydroxyl radicals) and type II ROS (^1^O_2_) under light illumination (Figure 4B–D; Figure S14, Supporting Information).^[^ ^15^ ^]^ Therefore, singlet oxygen might not be the sole factor to enable the proximity labeling of aggregated proteins, which was different from previously reported scenario in miniSOG enzyme and organic photosensitizers.^[^ ^16^ ^]^ To consolidate this mechanism, we used scavengers specific to the type I or type II photosensitizing pathway to block them individually. In line with our expectation, addition of NaN_3_ (singlet oxygen scavenger) or sorbitol (superoxide anion and hydroxyl radical scavenger) both significantly reduced the labeling efficiency (Figure 4E; Figure S15, Supporting Information). The labeling disappeared when NaN_3_ and sorbitol were used in combination, suggesting that both photosensitizing pathways contributed to proximity labeling (Figure 4E; Figure S15, Supporting Information). However, despite being a significant component of type I ROS, H_2_O_2_ appeared ineffective for proximity labeling (Figure 4E, lane 4; Figure S15, Supporting Information).

We next interrogated the labeling mechanism by searching for potential modification sites on aggregated proteins and deducing the covalent reaction mechanism using LC‐MS/MS experiment. To control the impact of residue abundance and distinct 3D structures of aggregated proteins on the labeling chemoselectivity preference, we selected three aggregated proteins (DHFR, Tau‐K18, and TTR) as our models. Upon P5 induced modification, we identified a wide scope of diverse amino acid residues as labeling sites, including lysine, proline, tryptophan, methionine, histidine, phenylalanine, arginine, cysteine, and tyrosine with different abundance (Figure 4F; Figures S16–S35 and Table S2, Supporting Information), highlighting the origin of its effective labeling. We scrutinized the tandem mass spectrometry results of labeled peptides and deduced different types of modification mechanisms, including the recently reported N‐O‐S reaction (Figure 4G,H; Figure S16, Supporting Information).^[^ ^17^ ^]^ Additionally, lysine, a readily labeled amino acid using the singlet electron transfer (SET) labeling mechanism, was also observed to be extensively labeled in this study (Figure 4I; Figure S33, Supporting Information).^[^ ^18^ ^]^ Importantly, different from previous reports, histidine was not top‐ranked in P5 enabled proximity labeling (Figure 4I; Figure S20, Supporting Information).^[^ ^19^ ^]^ Interestingly, by comparing the grand average of hydropathicity (GRAVY) of labeled peptides and full‐length proteins, we observed that these labeling sites in all three model proteins were predominantly located in the hydrophobic peptide region (Figure 4J; Tables S3–S5, Supporting Information). This result further supported the hypothesis that the hydrophobic effect governs P5's binding to aggregated proteins (Figure 2G; Table S1, Supporting Information). Additionally, the crosslinked products observed in gel analysis (Figure 3C) were analyzed by LC‐MS/MS to preliminarily explore the reaction types underlying protein photo crosslinking (Figures S36 and S37, Supporting Information).

### 2.4 Permeabilization of P5 for In‐Cell Imaging and Labeling of Aggregated Proteome

**Figure 5:**
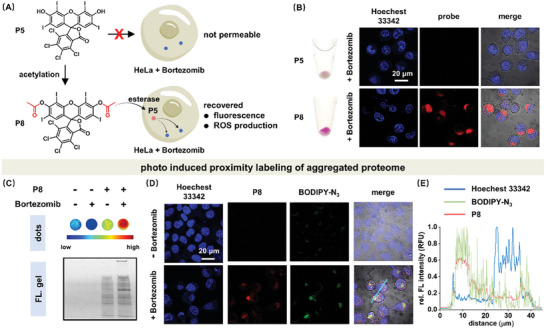
Permeabilization of P5 for in‐cell imaging and proximity labeling of aggregated proteome. A) Acetylation of P5 for cell permeabilization purpose. B) Bottom: P8 (5.0 µm) was cell permeable and imaged aggregated proteome upon proteasome inhibition by Bortezomib (0.8 µm) for 24 h. Upper: P8 accumulated in harvested cells. C) P8 (5.0 µm) selectively labeled misfolded and aggregated proteome in Bortezomib (0.8 µm) stressed cells under white light illumination (10 mW·cm^−2^). The illumination time was 20 min. The fluorescence signal of proximity labeling was from conjugation with TMR‐N_3_. D) The proximity labeling region (green fluorescence) colocalized well with the aggregated proteome stained by P8 (red fluorescence). The green fluorescence signal of proximity labeling was from conjugation with BODIPY‐N_3_. E) Colocalization image analysis on cyan line area in Figure 5D.

We next examined whether P5 can perform photo proximity labeling of aggregated proteome caused by inhibiting proteasome function using Bortezomib in HeLa cells (**Figure** **5**). Like most fluorescein‐based molecules, P5 was not envisioned to effectively penetrate cell membrane and stain aggregated proteome in live cells. To address this potential issue, we acetylated P5 (P8) to enhance its cell permeability. Upon deacetylation by endogenous esterase, P8 can convert back to P5 with restored fluorescence and photo‐oxidation properties (Figure 5A; Figure S38, Supporting Information). As expected and supported by confocal images, P8's fluorescence was restored inside the cell within 30 min and primarily located in the perinuclear aggresome caused by global proteome aggregation (Figure 5B, lower panel; Figure S39, Supporting Information), while P5 was not capable of penetrating the cell (Figure 5B, upper panel) allowed us to directly visualize its permeabilization and binding processes. In addition, cellular retention of dyes was exclusively observed in P8 but not P5 stained stressed cells (Figure 5B, bottom tube). No staining of cytoplasmic membranes by P8 confirmed its binding selectivity though it bears high hydrophobicity (Figure 5B, upper panel). Photo induced generation of ROS species was confirmed by DCFH‐DA assay, suggesting P8's successful conversion to the active P5 in cells (Figure S40, Supporting Information). Additionally, both dark toxicity and photo‐toxicity of P8 can be neglected when exposed to brief illumination duration (Figure S41, Supporting Information). Overall, P5 after acetylation can permeabilize into cells and selectively stain aggregated proteome.

We next explored its proximity labeling and crosslinking function for aggregated proteome in live stressed cells. Both dots (Figure 5C) and gel (Figure 5C; Figure S42, Supporting Information) experiments confirmed the labeling selectivity to aggregated proteome in stressed HeLa cells. Aggregated proteome crosslinking was also found in stressed cells (Figure 5C). One may notice that mild labeling of proteome can be detected even in non‐treated HeLa cells, which was caused by the presence of large amount of misfolded proteome in cancer cells due to massive protein synthesis out of cellular folding capacity.^[^ ^20^ ^]^ To validate the labeling actually occurred in proximity, we performed co‐localization imaging experiment to compare the fluorescence signals from P8 (P5) stained aggregated proteome (red fluorescence) and proximity labeling by BODIPY‐N_3_ (green fluorescence) (Figure 5D,E). As a characteristic feature of intracellular amorphous aggregated proteome, both colocalized red and green fluorescence formed perinuclear punctate aggresome structure in Bortezomib induced stressed HeLa cells, confirming the validity of P8 for proximity labeling. Furthermore, the general applicability of P8 was demonstrated using HEK‐293T and RAW264.7 cell lines (Figures S43 and S44, Supporting Information). Together, the enhanced permeability of P8 allows for photo induced proximity labeling of aggregated proteome in live cells.

### 2.5 P8 Profiles the Composition and Interaction of Cellular Aggregated Proteome

**Figure 6:**
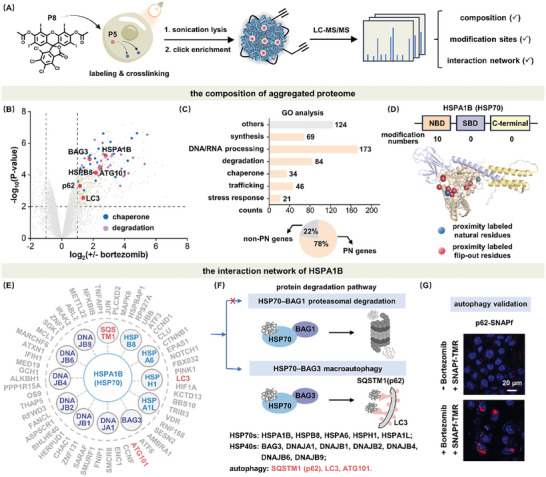
Profiling the composition and interaction of aggregated proteome. A) Scheme of proteomics analysis of aggregated proteome using LC‐MS/MS after in situ capture by proximity labeling. Cell samples were treated with 0.8 µm Bortezomib for 24 h to induce proteome aggregation and 5.0 µm P8 to stain and induce proximity labeling of aggregated proteome. Cell samples were illuminated under white light (10 mW·cm^−2^) for 20 min. B) Volcano plot of the significantly differentially expressed aggregated proteins upon Bortezomib treatment. Blue dots: chaperone related proteins. Purple dots: degradation related proteins. C) Gene Ontology (GO) analysis of up‐regulated aggregated proteins in stressed HeLa cells. The majority of up‐regulated aggregated proteins were functionally related to protein homeostasis network. D) Detailed labeling sites on HSPA1B (AlphaFold code: AF‐P0DMV9‐F1). Labeling primarily occurred on the NBD. Blue/Red dots: positive/negative correlation between the labeling frequency and the calculated solvent accessibility of the labeled sites. The solvent accessible surface area (SASA) values were calculated using NACCESS program. E) HSPA1B interaction network identified by P8 proximity labeling method. Most key components in BAG3‐mediated autophagy pathway were identified. F) BAG3‐mediated autophagy was preferentially activated for degrading aggregated proteins instead of the proteasomal degradation pathway following treatment of cells with Bortezomib for 24 h. G) Target validation by imaging the aggregation of p62‐SNAPf in stressed HeLa cells using TMR‐SNAPf.

Powered by P8's proximity labeling and crosslinking functions in cell, it is now possible to unveil layers of information regarding cellular aggregated proteome: 1) its proteomic composition; 2) proximity labeling sites; 3) proteins’ interaction network (**Figure** **6**A). Previously, it is technically difficult to in situ profile these lines of evidence in live cells due to the lack of “enrichment anchors” and the loosely packed nature of aggregated proteins. To this end, we first utilized P8 to selectively label aggregated proteome in proximity and profiled its composition via LC‐MS/MS analysis. We identified 551 up‐regulated proteins enriched in the aggregates from Bortezomib‐induced stressed HeLa cells (Figure 6B). The number of identified proteins (551) was almost double those obtained using traditional fractionation method shown by the Venn diagram (370, Figure S45 and Table S6, Supporting Information). Such improved protein ID may be gifted by P8 (P5)’s diverse labeling sites and reaction types via both radical and nucleophilic modifications in proximity (Figure 4)^[^ ^21^ ^]^ Gene ontology analysis showed that 78% of these enriched aggregated proteins were related to the proteostasis network (PN) (Figure 6C; Table S7, Supporting Information), which governs DNA/RNA processing, protein synthesis, folding, trafficking, degradation, and related stress responses. Specifically, most proteins with over fourfold of change can be classified into cell stress response and protein degradation functions (Table S8, Supporting Information). Observation of proteostasis components in cellular aggregates is hallmark evidence that supports the validity of our proteomics results as proteostasis is known for its surveillance role in preventing proteome aggregation.

Second, detailed investigation of modification sites revealed that HSPA1B (a HSP70 chaperone) was the most extensively labeled protein (Figure 6D, ten modification sites). Meanwhile, the labeling efficiency in stressed cells was much higher than that in non‐stressed cells (151 vs 11 spectral counts, Table S9, Supporting Information). As a member of the HSP70 chaperones that function in HSP70/40 refolding cycle, the high labeling frequency of HSPA1B suggested its active involvement against stress‐induced proteome aggregation to maintain proteostasis balance. We also observed a negative correlation between the labeling frequency and the calculated solvent accessibility of the labeled sites in HSPA1B (Figure 6D, red dots, Table S9, Supporting Information), suggesting its conformational fluctuation upon entangling in aggregates. Intriguingly, structural domain analysis of these labeling sites identified all modifications occurred at the nucleotide binding domain (NBD) of HSPA1B. The HSP70's NBD is known as an “anchor” to recruit misfolded proteins via HSP40s during the refolding process. Upon aggregation, misfolded proteins are stalled in proximity to the HSP70's NBD, which may explain the above observations.

Third, gifted by the in situ photo crosslinking property of P8 (P5) that stabilized aggregated complex, we depicted the interaction network of the above‐identified hub protein HSPA1B inside cellular protein aggregates. Such information was invisible by conventional fractionation method. As the main protein clearance systems, the ubiquitin‐proteasome system (UPS) and autophagy pathways are delicately evolved to handle misfolded and aggregated proteome in stressed cells in a cooperative manner.^[^ ^22^ ^]^ Co‐chaperones BAG1 and BAG3 were reciprocally regulated when cells experience acute stresses. Under physiological conditions, turnover of misfolded proteins is primarily regulated by the BAG1‐dependent UPS mechanism. On the other hand, under pathophysiological conditions, the BAG3‐driven autophagic degradation pathway is activated to compensate insufficient proteostatic degradation capacity.^[^ ^23^ ^]^ Intriguingly, major components in the BAG3‐mediated autophagy degradation pathway but not BAG1‐mediated ubiquitin‐proteasome system were identified through HSP70 (HSPA1B) interactions analysis (Figure 6E,F; Figures S46 and S47, Supporting Information).^[^ ^24^ ^]^ These key factors included HSP40 co‐chaperones (DNAJA1, DNAJB1, DNAJB2, DNAJB4, DNAJB6, DNAJB9, and BAG3), HSP70 chaperones (HSPA1B, HSPB8, HSPA6, HSPH1, and HSPA1L), and autophagosome biomarkers (p62, LC3, ATG101). To confirm the active participation of autophagy in clearing protein aggregates, we validated the identified p62 autophagy biomarker (BAG3 autophagy) by imaging the exaggerated aggregation of p62‐SNAPf in HeLa cells stressed by Bortezomib using TMR‐SNAPf probe (Figure 6G). Furthermore, upon addition of YM‐1, an inhibitor of HSP70‐BAG3,^[^ ^25^ ^]^ to Bortezomib‐induced stressed cells, a notable increase in aggresome fluorescence signal was observed, suggesting the involvement of BAG3‐mediated degradation pathway (Figure S48, Supporting Information).

Together, P8's photo induced proximity labeling and crosslinking functions identified aggregated proteins with improved coverage, precise modification sites with structural insights, and first‐glance of interaction information.

## 3 Conclusion

In this work, out of classic fluorescent and photosensitizer scaffolds, we identified Rose Bengal (P5) and its cell permeable acetylated derivative (P8) to effectively label and crosslink intracellular aggregated proteins in proximity. These functions were enabled by the unique binding affinity of P5 to different types of protein aggregates governed by the hydrophobic effect. Mechanistically, both type I and type II ROS generated by P5 contributed together to proximity labeling and crosslinking. Tandem mass spectrometry identified diverse modification sites and reaction mechanisms, which explained the rapid and efficient labeling. Permeabilization of P5 by acetylation allowed for effective labeling, crosslinking, and enrichment of cellular protein aggregates. Aggregated proteomics analysis revealed key players in BAG‐3‐mediated autophagy sequestered in the aggresome of stressed cells, which were mediated by the hub HSPA1B chaperone. The AggID platform reported herein combined P8's selective binding affinity, triplet photosensitizing labeling function, and residual singlet fluorescence property to in situ image and profile cellular aggregated proteome. The method reported in this work also suffers from non‐specific binding of probes to hydrophobic microenvironment in cellular milieu (e.g., membrane proteins, hydrophobic protein domains, lipid droplets), causing potential background issue and false‐positive hits. Thus, background correction for proteomics analysis is necessary to count for these technical limitations.

## 4 Experimental Section

### Measurement of *K*_d_ Value between Probe and Aggregated Protein

Solutions of probe (20.0 µm, final concentration) and aggregated proteins of gradient concentrations (from 0.1 to 100.0 µm) were mixed and incubated at ambient temperature for 20 min. Subsequently, the mixture was centrifuged at 13 000 rpm and 4 °C for 20 min to separate the solid and liquid components. The absorbance of the probe in the soluble solution was quantified by Tecan Spark Fluorescence Plate Reader in BeyoGold 96‐Well transparent plate (abs1). For comparison, the absorbance of 20.0 µm probe was also measured (abs2). Next, the partition of probe in aggregated proteins could be calculated by the difference between abs1 and abs2. The dissociation constant (*K*_d_) of the aggregated protein‐probe interaction was then obtained by fitting the partition of probe in aggregated protein and the concentrations of the proteins to the equation as below, using GraphPad 6.0.

(1) $$Y\;=\frac{X*B_{max}}{K_{d}+X}\;$$

The variable Y represented the proportion of the probe that partitioned in the aggregated protein, while *B*_max_ denoted the maximum value. The variable X denoted the concentration of aggregated proteins.^[^ ^26^ ^]^

### Proximity Labeling in Aggregated Proteins for Gel Analysis and Dots Experiments

The aggregated protein (2 mg·ml^−1^, final concentration), which was prepared as depicted above, was mixed with probes (20.0 µm) to incubate under ambient temperature for 20 min. Then, a 10 mm substrate probe (various amine compounds) was added to the reaction system before white light illumination (25 mW·cm^−2^). The obtained aggregated protein which was labeled by substrate probe was next precipitated by acetone at −20 °C for at least 4 h to remove any unlabeled substrate probe on the following click treatment. The resulting precipitated aggregated protein was re‐dissolved in 4% SDS and then reacted with freshly prepared click solution (1.7 mm tert‐Butyl 2,2,2‐trichloroacetimidate (TBTA), 50.0 mm CuSO_4_, 50.0 mm Tris (2‐carboxyethyl) phosphine (TCEP), and 50.0 mm tetramethylrhodamine azide (TMR‐N_3_)) in the dark for 1 h. Acetone precipitation was done again to remove excess click reagent and the obtained precipitated aggregated protein was subsequently dissolved in 10% SDS for further SDS‐PAGE electrophoresis and Dots experiments. The labeling results could be visualized by the Tanon‐2500BR system for SDS‐PAGE imaging and the VISQUE InVivo Smart‐LF bio‐imaging system for dots experiments, respectively.

For experiments defining the impact of specific reactive oxygen species (ROS) type on proximity labeling, 20.0 mm sorbitol, 20.0 mm NaN_3_ was pre‐added to the reaction system before white light illumination to eradicate radicals (Type I ROS) and ^1^O_2_ (Type II ROS), respectively.^[^ ^10c^ ^]^

### ROS Definition

For singlet oxygen (^1^O_2_) detection, commercially available kit, 9,10‐anthracenediyl‐bis(methylene) (ABDA) was utilized.^[^ ^27^ ^]^ Specifically, 1.0 mm stock solution of ABDA was prepared in DMF and stored at −20 °C with light‐free. In practice use, the working concentration was 0.1 mm and the absorbance of ABDA reacted with singlet oxygen produced by probes (3.0 µm) at 378 nm was recorded at different light illumination time; For superoxide anions (O_2_^•−^) detection, commercially available kit, dihydrorhodamine 123 (DHR 123) was utilized.^[^ ^28^ ^]^ Specifically, 1.0 mm stock solution of DHR123 was prepared in DMF and stored at −20 °C with light‐free. In practice use, the working concentration was 6.0 µm and the fluorescence at 525 nm of rhodamine123 which was the reaction product of DHR 123 and superoxide anions was recorded at different light illumination time under the excitation of 480 nm; For hydrogen radical (•OH) detection, commercially available kit, hydroxyphenyl fluorescein (HPF) was utilized.^[^ ^29^ ^]^ Specifically, 1.0 mm stock solution of HPF was prepared in DMF and stored at −20 °C with light‐free. In practice use, the working concentration was 3.0 µm and the fluorescence at 514 nm of fluorescein which was the reaction product of HPF and hydrogen radical was recorded at different light illumination time under the excitation of 480 nm.

For type I reactive oxygen scavenging, sorbitol was used. Specifically, 100.0 mm stock solution of sorbitol was prepared distilled water and stored in dark at −20 °C. In practice use, the working concentration was 10.0 mm to eradicate type I reactive oxygens produced by 20.0 µm probe; For type II reactive oxygen scavenging, NaN_3_ was used. Specifically, 100.0 mm stock solution of sorbitol was prepared in distilled water and stored in dark at −20 °C. In practice use, the working concentration was 10.0 mm to eradicate type II reactive oxygens produced by 20.0 µm probe.

### Intracellular ROS Detection

The generation of ROS by P8 in HeLa cells was monitored using the Reactive Oxygen Species Assay Kit (CA1410, Solarbio).^[^ ^30^ ^]^ HeLa cells were seeded into 96‐well plate and cultured at 37 °C, 5% CO_2_ atmosphere. After reaching 80% cell density, the cells were treated with P8 (5.0 µm) for 6 h. The reactive oxygen species assay was performed following the provided instructions of the commercial assay kit. To induce intracellular ROS production, the cells were exposed to white light (3 mW·cm^−2^) for 15 min. Fluorescence images were collected using Olympus IX73 Research Inverted Microscope. The cells were excited using the blue light channel (460–490 nm), and emission images were collected using the green light channel (500–530 nm).

### Aggregated Proteome Proximity Labeling in HeLa Cells

HeLa cells were seeded and cultured in 35 mm confocal dish and 25 cm^2^ culture flask for fluorescence and gel analysis, respectively before treatment of Bortezomib (0.8 µm) and P8 (5 µm) for 24 h. For fluorescence analysis, cells were next fixed and permeabilized with 4% formaldehyde and 0.1% Triton X‐100 at room temperature consistently for 30 min. The cells were then added with propargylamine (10.0 mm) before white light illumination for 20 min. Freshly premixed click solution (1.7 mm TBTA, 50.0 mm CuSO_4_, 50.0 mm TCEP, and 50.0 mm BODIPY‐N_3_) was finally added to pretreated cells and reacted for 1 h. Cells were washed with PBS three times to remove unreacted click solution and treated with Hoechst 33342 before imaging with Olympus FV1000 FluoViewTM confocal microscope. For gel analysis, cells were incubated with propargylamine (10.0 mm) before white light illumination for 20 min. Next, cells were ultrasonic lysed with 1% SDS followed by reaction with newly prepared click solution (1.7 mm TBTA, 50.0 mm CuSO_4_, 50.0 mm TCEP, and 50.0 mm TMR‐N_3_). The obtained proteome samples were precipitated with acetone before gel analysis.

### Peptide Hydrophobicity Analysis

The grand average of hydropathicity index (GRAVY) was employed to quantify the hydrophobicity value of a peptide, by summing the hydropathy values of all the amino acids and dividing the result by the sequence length. The calculation of GRAVY was based on the hydropathy values established by Kyte and Doolittle.^[^ ^31^ ^]^ Positive GRAVY values indicated hydrophobicity, whereas negative values indicated hydrophilicity. All these physicochemical values could be obtained directly from the Expasy website (https://web.expasy.org/protparam/).^[^ ^32^ ^]^

### Solvent Accessible Surface Area (SASA) Analysis

The structure of HSPA1B (HSP70) used for solvent accessible surface area (SASA) analysis were obtained from the Protein Data Bank (PDB). The absolute SASA value of each labeling residue was calculated using NACCESS program.^[^ ^33^ ^]^

## Conflict of Interest

The authors declare no conflict of interest.

## Author Contributions

H.F. and Q.Z. contributed equally to this work. H.F. performed the experiments and wrote the manuscript draft. Q.Z. performed the experiments and revised the manuscript. N.Z. and Z.L. prepared the samples for LC‐MS/MS analysis. Y.H. and X.Z. contributed to the cell imaging part., Y.L. and L.Z. conceptualized this work and revised it.

## Supporting information

Supporting Information

## Data Availability

The data that support the findings of this study are available in the supplementary material of this article.
