# Supplementary material for: A Cell‐Permeable Photosensitizer for Selective Proximity Labeling and Crosslinking of Aggregated Proteome
Source: Adv Sci (Weinh). 2024 Mar 5;11(18):2306950. doi: 10.1002/advs.202306950 (PMC11095223; doi:10.1002/advs.202306950)
Supplement: Supplementary file 1 — Supporting Information [file ADVS-11-2306950-s001.pdf]

ADVANCED SCIENCE
Open Access

Supporting Information

for *Adv. Sci.*, DOI 10.1002/advs.202306950

A Cell-Permeable Photosensitizer for Selective Proximity Labeling and Crosslinking of Aggregated Proteome

Huan Feng, Qun Zhao, Nan Zhao, Zhen Liang, Yanan Huang, Xin Zhang, Lihua Zhang and Yu Liu**

Supporting Information

A Cell-Permeable Photosensitizer for Selective Proximity Labeling and Crosslinking of Aggregated Proteome

Huan Feng, Qun Zhao, Nan Zhao, Zhen Liang, Yanan Huang, Xin Zhang, Lihua Zhang, and Yu Liu**

Table of Contents

Table of Contents 2
1. **Experimental Procedures** 3
1.1 Plasmids construction and protein purification 3
1.2 Aggregation conditions of different model proteins 3
1.3 Partition of probe in aggregated proteins 3
1.4 Conditions to obtain different states of DHFR protein 4
1.5 Model protein (WT-DHFR, TauK18-WT, or WT-TTR) digestion 4
1.6 Mass spectrometry analysis 4
1.7 Cell samples preparation for mass analysis and proteome digestion 4
1.8 Mass spectrometry analysis Mass data analysis 5
1.9 Mass data analysis 5
2. **Supporting Figures and Tables** 6
3. **Synthesis and Characterizations** 71
4. **References** 73

1. Experimental Procedures

1.1 Plasmids construction and protein purification.

Genes of *E. coli* wild type dihydrofolate reductase (WT-DHFR), sortase, mutant superoxide dismutase 1(SOD1(V31A)-Halo), HaloTag, wild type transthyretin (WT-TTR), α-synuclein and Tau-K18 wild type (Tau-K18-WT) were all optimized and synthesized by GenScript, Nanjing, China, and sub-cloned into pET-29b(+) vectors. DHFR (WT-DHFR), sortase, SOD1(V31A)-Halo, Halo and Tau-K18 were cloned with His-tag at their C-termini for easy purification purpose. No His-tag at C-termini of WT-TTR and α-synuclein was due to potential interferences of amyloid formation.

As previously reported. Briefly, for WT-DHFR, sortase, SOD1(V-31A)-Halo, HaloTag and WT-TTR: the plasmids were transformed into BL21(DE3) *E. coli* cells. Cells were then grown in Luria Bertani (LB) media and incubated at 37 °C under continuous shaking until the OD_{600} was in the range of 0.6-0.8. Protein synthesis was subsequently induced by isopropyl β-Dthiogalactoside (IPTG) at various conditions (WT-DHFR: 37 °C for 4 h, Sortase: 30 °C for 16 h, SOD1(V31A)-Halo: 18 °C for 16 h, Halo: 37 °C for 4 h, WT-TTR: 37 °C for 4 h). Then, cells re-suspended in buffer A (50.0 mM Tris·HCl, 100.0 mM NaCl, pH = 8.00) and lysed by sonication at 4 °C. The supernatant obtained by centrifugation (12,000 rpm, 30 min) was loaded into a 10 mL Ni-NTA column and then was eluted by buffer A (50.0 mM Tris·HCl, 100.0 mM NaCl, pH = 8.00) with gradient increasing of buffer B (50.0 mM Tris·HCl, 100.0 mM NaCl, 500.0 mM imidazole, pH = 8.00). Ni-NTA column pre-purified proteins were further purified by using a 120 mL Superdex 200 size-exclusion column in phosphate buffer (10.0 mM sodium phosphates, 100.0 mM KCl, 1.0 mM EDTA, pH = 7.40). The protein containing fractions were identified by SDS-PAGE electrophoresis gel analysis, then pooled, and concentrated. No significant impurity was identified and the purity was estimated to be at least 98% based on SDS-PAGE gel.[1]

For TauK-18-WT: the plasmids were transformed into BL21(DE3) *E. coli* cells. Cells were then grown in Luria Bertani (LB) media and incubated at 37 °C under continuous shaking until the OD_{600} was in the range of 0.6-0.8. Protein synthesis was subsequently induced by IPTG at 16 °C overnight. Then, cells were harvested and resuspended in lysis buffer (50.0 mM Tris·HCl, 500 mM NaCl, and 10 mM imidazole, pH = 8.00) before sonication lysing at 4 °C. The supernatant obtained by centrifugation (12,000 rpm, 30 min) was loaded into a 10 mL Ni-NTA column and then was washed by buffer (50.0 mM Tris·HCl, 500 mM NaCl, and 10 mM imidazole,

pH = 8.00). The eluent buffer was then exchanged back to 10 μM imidazole buffer during an incubation with 1 mM DTT and 1~2 ml 5U·μL^{-1} TEV protease overnight at 4 °C and further run over the Ni-NTA column. Further purification of proteins was carried on Superdex 200 size-exclusion column in phosphate buffer (10.0 mM sodium phosphates, 100.0 mM KCl, 1.0 mM EDTA, pH = 7.40). The protein containing fractions were identified by SDS-PAGE electrophoresis gel analysis, then pooled, and concentrated. No significant impurity was identified and the purity was estimated to be at least 95% based on SDS-PAGE gel.[2]

For α-synuclein: the plasmids were transformed into BL21(DE3) *E. coli* cells. Cells were then grown in Luria Bertani (LB) media and incubated at 37 °C under continuous shaking until the OD_{600} was in the range of 0.3-0.4. Protein synthesis was subsequently induced by IPTG at 37 °C for 5 h. Then, cells were harvested and resuspended in osmic shock buffer (30.0 mM Tris·HCl, 40% sucrose, and 2 mM EDTA disodium, pH = 7.20) and incubated at room temperature for 10 min. The pallet obtained by centrifugation (12,000 rpm, 20 min) was resuspended quickly with cold water followed by addition of saturated $MgCl_2$ and keeping on ice for 3 min. The crude proteins were obtained by centrifugation (12,000 rpm, 20 min) and further dialyzed against by buffer A (50.0 mM Tris·HCl, 100.0 mM NaCl, pH = 8.00) overnight. After another centrifugation at 12,000 rpm for 20 min, the supernatant was loaded onto Q-Sepharose Fast Flow column (company) and eluted with a 0.0-0.5 M NaCl gradient in buffer A. The protein containing fractions were identified by SDS-PAGE electrophoresis gel analysis, then pooled, and concentrated. No significant impurity was identified and the purity was estimated to be at least 98% based on SDS-PAGE gel. For amyloid formation assay, the protein buffer was exchanged to phosphate aggregation buffer using SEC chromatography.

1.2 Aggregation conditions of different model proteins.

For aggregated WT-DHFR: Freshly purified DHFR protein (100.0 μM, final concentration) was incubated in aggregation buffer (NaOAc 200.0 mM, KCl 100.0 mM, pH = 6.23) at 65 °C for 5 min.

For aggregated sortase: Freshly purified sortase protein (100.0 μM, final concentration) was incubated in aggregation buffer (NaOAc 200.0 mM, KCl 100.0 mM, pH = 6.23) at 80 °C for 5 min.

For aggregated SOD1-(V31A)-Halo: Freshly purified DHFR protein (100.0 μM, final concentration) was incubated in buffer A (Tris·HCl 50.0 mM, NaCl 100.0 mM, pH = 8.00) at 95 °C for 5 min.

For aggregated HaloTag: Freshly purified HaloTag protein (100.0 μM, final concentration) was incubated in aggregation buffer (NaOAc 200.0 mM, KCl 100.0 mM, pH = 6.23) at 65 °C for 5 min.

For aggregated WT-TTR: Freshly purified WT-TTR protein (25.0 μM, final concentration) was incubated in acidic aggregation buffer (NaOAc 200.0 mM, KCl 100.0 mM, pH = 4.40) at 37 °C for 72 h.

For aggregated TauK18-WT: Freshly purified TauK18-WT (100.0 μM, final concentration) was incubated in aggregation buffer (sodium phosphates 10.0 mM, KCl 100.0 mM, EDTA 1.0 mM, pH = 7.40, heparin 2.5 μM, DTT 1 mM) at 37 °C for 72 h.

For α-synuclein: Freshly purified α-synuclein (100.0 μM, final concentration) was incubated in aggregation buffer (PBS 100 mM, pH = 7.40) and then was shaken on a Heidolph vibrating platform shaker at 37 °C with 1,350 rpm for 72 h.

1.3 Partition of probe in aggregated proteins.

Aggregated protein (100.0 μM, final concentration), which was prepared as depicted above, was mixed with probes (20.0 μM) (corresponding 100.0 μM folded protein was together mixed for Figure 2B) to incubate under ambient temperature for 20 min. Afterward, the mixture was centrifuged at 13,000 rpm at 4 °C for 20 min to separate the solid and liquid portions. The absorbance of the probes in the soluble solution was measured using a Tecan Spark Fluorescence Plate Reader in a transparent BeyoGold 96-Well plate (abs1). As a comparison, the absorbance of the 20.0 μM probe alone was also measured (abs2). The partition of the probe in the aggregated proteins was calculated by subtracting abs2 from abs1. This experiment was repeated three times. Photographs of the probe alone in buffer and in the centrifuged pellet were taken using a cell phone.

1.4 Conditions to obtain different states of DHFR protein.

Folded DHFR: folded state DHFR was prepared in phosphate buffer (sodium phosphate 10.0 mM, KCl 100.0 mM, EDTA 1.0 mM, pH = 7.40) at 25 °C.

Unfolded DHFR: unfolded state DHFR was in 6.0 M urea at 4 °C overnight.

Misfolded DHFR: misfolded state DHFR was prepared in phosphate buffer (sodium phosphate 10.0 mM, KCl 100.0 mM, EDTA 1.0 mM, pH = 7.40) at 65 °C for 5 min.

Aggregated DHFR: aggregated state DHFR was prepared in acidic aggregation buffer (NaOAc 200.0 mM, KCl 100.0 mM, acidified by AcOH to pH = 6.23) at 65 °C for 5 min.

1.5 Model protein (WT-DHFR, TauK18-WT, or WT-TTR) digestion.

For labeling sites identification of model protein (WT-DHFR, Tau-K18-WT, or WT-TTR), aggregated protein (2.0 mg·mL^{-1}) and P5 probe (20.0 μM) was incubated at ambient temperature for 20 min. Then, 10.0 mM substrate probe was added to the reaction system before white light illumination (25 mW·cm^{-2}) for 1 h. After acetone precipitation, the precipitated oxidated aggregated protein was re-dissolved in 6.0 M urea.

Next, these proteins were reducted with 5.0 mM DTT for 1 h and alkylated with 12.5 mM IAA for 20 min at room temperature in the dark. The sample was diluted to 1.0 M urea with 50.0 mM ammonium bicarbonate (ABC). They were digested with trypsin at an enzyme-to-protein ratio of 1:20 (w/w) at 37 °C for 14 h. Finally, the resulting peptides were desalted using homemade C18 tips (5 μm, 150 A, Venusil XBP) desalted. For C18 tips, solvent B (80% acetonitrile/H_2O, 0.1% FA, v/v) was added to activate the C18 silicon particles three times by centrifugation (500 g, 5 min). After activation, the C18 tips were equilibrated by solvent A (0.1% FA/H_2O, v/v) three times by centrifugation (500 g, 5 min). The peptides were loaded onto the C18 tips and washing with solvent A three times

by centrifugation (500×g, 5 min) for desalted. Finally, the resulting peptides were eluted by solvent B twice by centrifugation (300×g, 10 min). Then, the resulting peptides were lyophilized and subsequently subjected to nano-LC-MS/MS analysis. All samples were technically repeated three times.

1.6 MTT assay.

Dark cytotoxicity and photo cytotoxicity of P8 in HeLa cells was assessed by MTT assay.[3] Specifically, HeLa cells were seeded into 96-well plate and cultured at 37 °C, 5% CO_2 atmosphere. P8 treatment (0.0, 1.0, 2.5, 5.0, 7.5, 10.0, 12.5, 15.0, 17.5, 30.0, 50.0, 70.0, 80.0 μM) was done when the cell density reached 80%. After a 24 h incubation, 10.0 μL MTT (5.0 mg·mL^{-1}) was added to each well and cells were incubated for an additional 4 hours. Then, the supernatant media was carefully removed and the purple formazan left was dissolved thoroughly with 150.0 μL DMSO. Cell viability was determined by measuring absorbance at 490 nm. Error bars: standard error (n = 3). For photo cytotoxicity, cells pretreated with P8 (5.0 μM) require an additional white light illumination for 0-60 min.

1.7 Cell samples preparation for mass analysis and proteome digestion.

HeLa cells were seeded and cultured in 75 cm^2 culture flask and then treated with Bortezomib (0.8 μM) and P8 (5.0 μM) for 24 h. In the control group, HeLa cells were treated with P8 (5.0 μM) only for 24 h. After treatment, all cells were incubated with propargylamine (PA, 10.0 mM) in the dark for 20 min, followed by white light illumination for 20 min. For the click reaction of the proteins, cells from the experimental group and control group were resuspended in 1% (w/v) SDS/PBS by sonication at 80 W on ice and diluted into 0.2% SDS with 1× PBS. Then, 90.0 μL of 20.0 mM dialkoxydiphenylsilane (DADPS) biotin azide (biotin-N_3, click chemistry tools), 45.0 μL of 160 mM tris(3-hydroxypropyltriazolyl-methyl) amine, 45.0 μL of 20.0 mM $CuSO_4$, and 45.0 μL of 500.0 mM vitamin C were added in series to initiate the click reaction. The sample was kept at 60 °C for 2 h with constant shaking. A 4-fold excess of precooled acetone was added to the sample followed by incubation at -20 °C overnight and washed three times with precooled acetone to completely remove the excess DADPS biotin azide. Next, the labeled proteins dissolved in 8.0 M urea and diluted to 1.0 M urea with 50.0 mM NH_4HCO_3. Then the sample was incubated with streptavidin agarose resin (17511301, cytiva) at room temperature for 2 h with gentle rotation. The beads were then washed twice with 8.0 M urea and 2.0 M NaCl solution and resuspended in 0.3 M urea. Next, 10.0 mM dithiothreitol was added and incubated at room temperature for 1 h. Subsequently, 20.0 mM iodoacetamide aqueous solution was added and incubated at room temperature for 30 min in the dark. After centrifugation, beads were washed twice with NH_4HCO_3 solution (50.0 mM) followed by the treatment of trypsin at 37 °C for 12 h at an enzyme to protein ratio of 1:30 (w/w), and the released peptides in supernatant were collected by centrifugation. Then beads were washed with 1.0 M KCl in 1× PBS, and the collected supernatant was merged with the above released peptides for component identification. Thereafter, the peptides binding on beads were released by eluting with 5-fold volume of 10% formic acid (FA) for three times, and the collected peptides in supernatant were merged for site identification.

For peptides, using homemade C18 tips (5 μm, 100 Å; Durashell) desalted and fractionated. After activated and equilibrated the C18 tips, the peptides were loaded onto the C18 tips and washing with solvent A (H_2O, ammonia was added until pH reached 10.0) twice for desalted. The elution solvent consisted of solvent A and solvent B (acetonitrile, same volume of ammonia was added to solvent B as A), mixed into 9 eluates (6%, 9%, 12%, 15%, 18%, 21%, 25%, 30%, 80%B), combining 6% and 25% for fraction 1, 9% and 30% for fraction 2, 12% and 80% for fraction 3, 15%, 18% and 21% are fraction 4, 5 and 6, respectively. All other steps are the same as 'homemade C18 tips desalted'. Then, the resulting peptides were desalted using homemade C18 tips desalted. Finally, the resulting peptides were lyophilized and subsequently subjected to nano-LC-MS/MS analysis. Three biological replicates were performed for cell samples with and without Bortezomib treatment.

1.8 Mass spectrometry analysis.

The dried tryptic digestion samples were initially re-dissolved in a solution containing 0.1% formic acid (FA). These samples were later analyzed using an Easy-nano LC 1200 system, which was coupled to an Orbitrap Exploris 480 instrument equipped with a FAIMS Pro device (Thermo Fisher Scientific). During FAIMS separations, the inner and outer electrode temperatures were maintained at 100 °C, and the total carrier gas flow rate was set at 4.0 L·min^{-1}. The CV (compensation voltage) values used for each injection were -45 and -65. For the mass spectrometry (MS) analysis, two mobile phases were employed. Mobile phase A consisted of 0.1% FA in HPLC H_2O, while mobile phase B consisted of acetonitrile with 20% water and 0.1% formic acid (FA). Separation of peptides using nano-RPLC (reversed-phase liquid chromatography) was carried out using a C18 capillary column with dimensions of 150 μm i.d. × 300 mm, packed with C18 silica particles (1.9 μm, 120 Å) from Dr. Maisch GmbH, Beim Brueckle, Germany. The column was heated to 55 °C, and peptide separation was performed at a flow rate of 600 nL·min^{-1}. The following gradient was used for the separation: starting from 3% B (mobile phase B) and increasing to 8% B over 5 minutes, followed by a gradient from 8% to 28% B in the next 80 minutes, then an increase from 28% to 38% B over 17 minutes, further increasing to 100% B in 8 minutes, and maintaining 100% B for 10 minutes. The mass spectrometer was operated in positive ion mode, and the MS analysis was carried out using a data-dependent acquisition (DDA) mode. The MS1 scans were performed with a resolution of 60,000 (at 200 m/z) from m/z 350 to 1500. The MS2 scans were conducted with a resolution of 15,000, and the first mass was set at 110. The maximum injection times for MS1 and MS2 were 20 and 30 ms. In each full MS scan, the most intense ions with charge states ranging from 2 to 7 were selected for monomer oxidation sequencing. The selection was performed in a cycle time of 1 second, using an isolation window of 1.6 m/z. The fragmentation of precursor ions was achieved using the data-dependent HCD (higher-energy collisional dissociation) mode, with a normalized collision energy set to 30%.

1.9 Mass data analysis.

The protein monomer labeling sequencing raw files were initially processed using pFind 3.2.0 software against the Human Uniprot FASTA database. Trypsin was designated as the enzyme, allowing for the allowance of up to 3 potential missed cleavage sites. Carbamidomethyl (C) was chosen for fixed modifications, oxidation on methionine, acetylation of the protein N-terminus and probe-induced modified sites and mass-shift listed in Table S2 were set as variable modifications. Mass tolerance of MS1 and MS2 scans was set to 20 ppm and peptides were filtered with the false discovery rate (FDR) ≤ 1%.

For the quantitative proteomic analysis of aggregated proteins induced by Bortezomib, we searched the raw MS files (with a charge state of 2-7) in MaxQuant software using label-free quantification. The parameters for the search were as follows: - The human protein database was downloaded from UniProt (20422 entries, reviewed). - Trypsin was used as the digestion enzyme with a maximum of 2 allowed missed cleavages. - Carbamidomethyl (Cysteine, +57.0214 Da) was set as a fixed modification. - Oxidation (Methionine, +15.9949 Da), Acetyl (Protein N-term, +42.0106 Da), and probe-induced labeling were included as variable modifications. - The false discovery rate (FDR) for proteins and peptides was set to 0.01. - "Match between runs" was enabled for label-free identification. - The proteins quantified in all three replicates were subjected to Student's t-test estimations, and those that passed the 0.05 threshold were further analyzed for differences. A 2-fold change cutoff was considered significant.

The mass spectrometry data and searching results have been deposited in the ProteomeXchange Consortium repository of open source under the dataset identifier PXD044948. The data can be accessed through the following link: https://proteomecentral.proteomexchange.org/cgi/GetDataset?ID=PXD044948.[4]

2. Supporting Figures and Tables

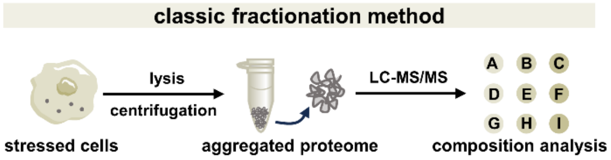

Figure S1. Conventional fractionation method to separate amyloid aggregated proteins and analyse their composition.[5]

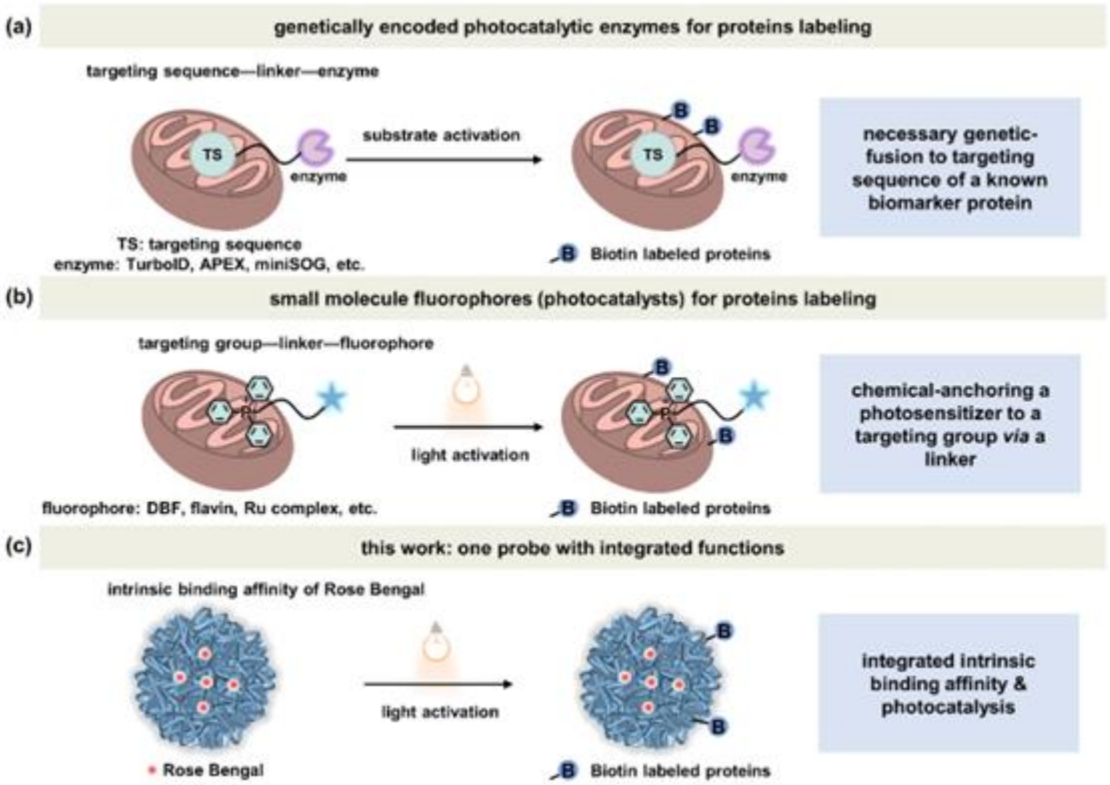

Figure S2. Design principles of previous reported proximity labeling methods and the AggID method reported in this work.[6] Previous methods, including APEX, TurboID, MiniSOG (a) or small molecule fluorophore (b) mediated proximity labeling, all require to anchor the photocatalytic enzymes to a known biomarker or fluorophores to a targeting probe via a linker. The fusion of biomarker and enzyme/fluorophore was tedious and complicated. (c) This work introduced an "all-in-one" small molecule design concept with integrated "targeted binding" and "photo-catalytic labeling" functions in one probe without knowing the biomarker. The AggID probe developed in this work is the first probe with both binding affinity to aggregated proteome and photocatalytic labeling functions in one probe.

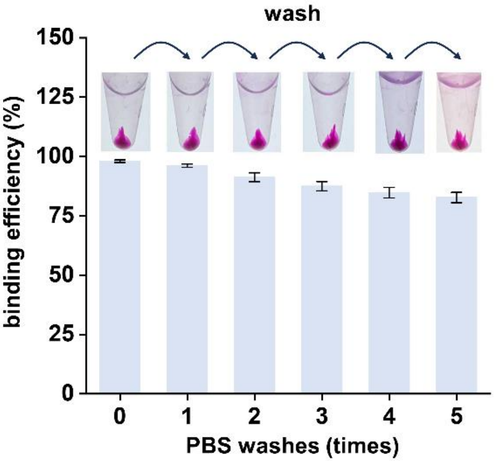

Figure S3. P5 maintained high binding affinity to aggregated DHFR even after five times PBS washing. DHFR (100.0 µM) was aggregated by heating the sample at 65 °C for 5 min in aggregation buffer (NaOAc 200.0 mM, KCl 100.0 mM, acidified by AcOH to pH = 6.23). 20.0 µM P5 was added to the prepared protein samples and then kept in dark for 20 min before further treatment. Error bars: standard error (n = 3).

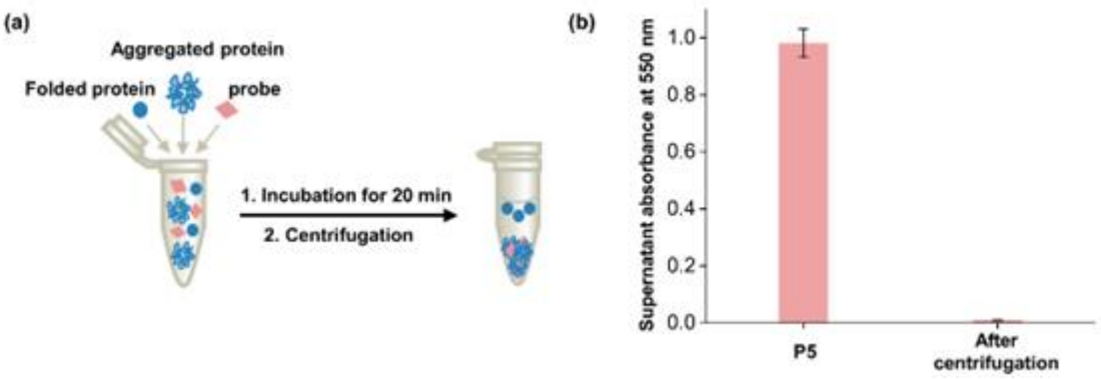

Figure S4. Selectivite binding of P5 to aggregated DHFR over folded ones. (a) Scheme of sample preparations: folded DHFR (100.0 µM), aggregated DHFR (100.0 µM), and P5 probe (20.0 µM) were mixed and incubated for 20 min, and then centrifugated at 13,000 rpm for 30 min at 4 °C. Aggregated DHFR protein was prepared by heating in aggregation buffer (NaOAc 200.0 mM, KCl 100.0 mM, acidified by AcOH to pH = 6.23) at 65 °C for 5 min. (b) Residual absorbance of P5 at 550 nm in supernatant (NaOAc 200.0 mM, KCl 100.0 mM, acidified by AcOH to pH = 6.23) after fractionation was measured to vigorously examine the selectivity in presence of folded proteins. Error bars: standard error (n = 3).

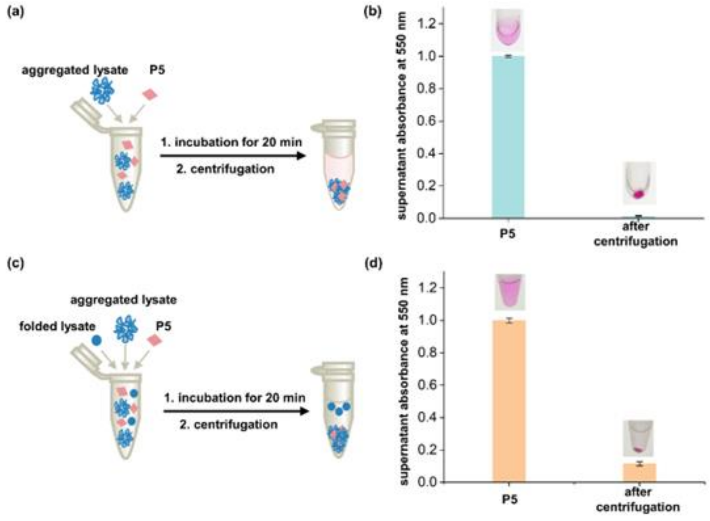

Figure S5. Selective binding of P5 to aggregated *E. coli* lysate. (a) Scheme of sample preparations to examine the binding selectivity of P5 to aggregated *E. coli* lysate. (b) Residual absorbance of P5 at 550 nm in supernatant after fractionation was measured following the scheme in (a). (c) Scheme of sample preparations to examine the binding selectivity of P5 to aggregated *E. coli* lysate in the presence of folded lysate. (d) Residual absorbance of P5 at 550 nm in supernatant after fractionation was measured following the scheme in (c). Aggregated *E. coli* lysate was prepared by heating in aggregation buffer (NaOAc 200.0 mM, KCl 100.0 mM, acidified by AcOH to pH = 6.23) at 65 °C for 5 min. Error bars: standard error (n = 3).

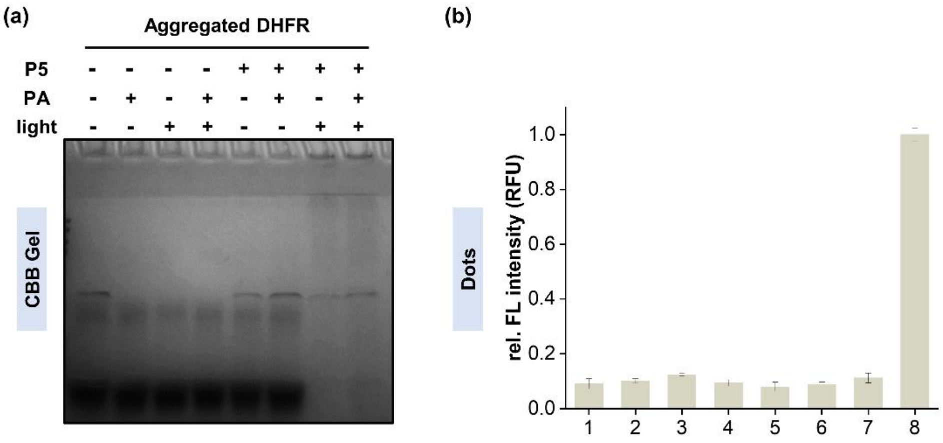

Figure S6. P5 induced aggregated DHFR proximity labeling using propargylamine as the labeling substrate (refers to **Figure 3B**). (a) Coomassie brilliant blue (CBB) gel corresponding to the FL gel in **Figure 3B**. (b) Quantification of dots experiments in **Figure 3B**. Protein concentration: 2.0 mg·mL^{-1}; P5 concentration: 20.0 μM; Propargyl amine: 10.0 mM; Light intensity: 25 mW·cm^{-2}; Illumination time: 20 min. Dots experiments were performed on nitrocellulose film and imaged using VISQUE InVivo Smart-LF bio-imaging system. Error bars: standard error (n = 3).

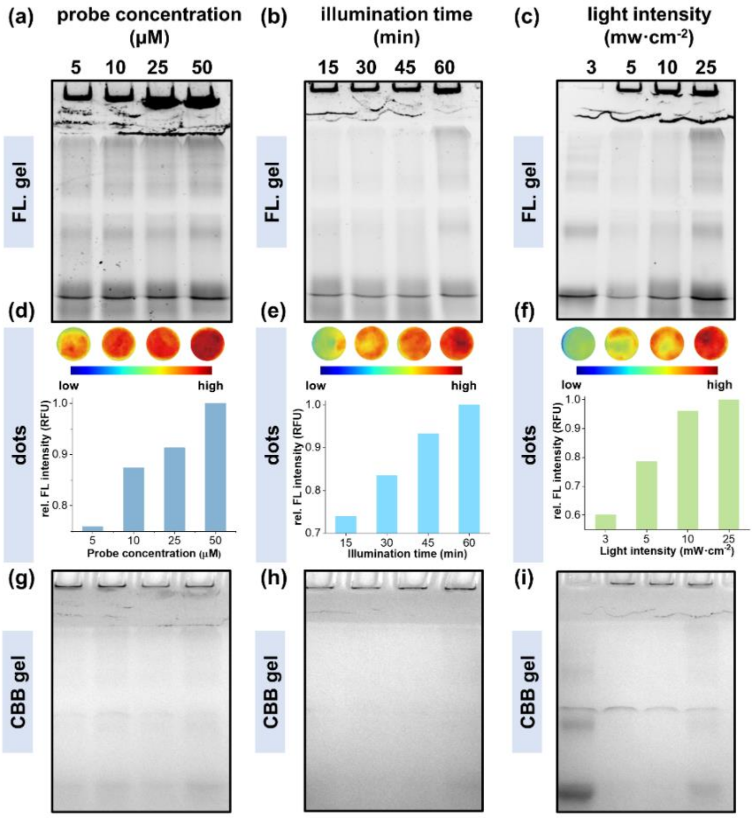

Figure S7. Photo induced proximity labeling and crosslinking efficiency using P5 probe was dependent on probe concentration, illumination duration, and illumination intensity. (a), (d), and (g) Increasing probe concentrations (5.0, 10.0, 25.0, 50.0 µM, respectively) improved labeling and crosslinking efficiency; (b), (e), and (h) Increasing illumination time (15, 30, 45, 60 min, respectively) improved photo labeling and crosslinking efficiency; (c), (f), and (i) Increasing light intensity (3, 5, 10, 25 mW·cm^{-2}, respectively) improved labeling and crosslinking efficiency. DHFR (2.0 mg·mL^{-1}) was aggregated by heating the sample at 65 °C for 5 min in aggregation buffer (NaOAc 200.0 mM, KCl 100.0 mM, acidified by AcOH to pH = 6.23). 20.0 µM P5 was added to the prepared protein samples and kepte in dark for 20 min before further treatment. Propargyl amine: 10.0 mM; Light intensity: 25 mW·cm^{-2}; Illumination time: 30 min.

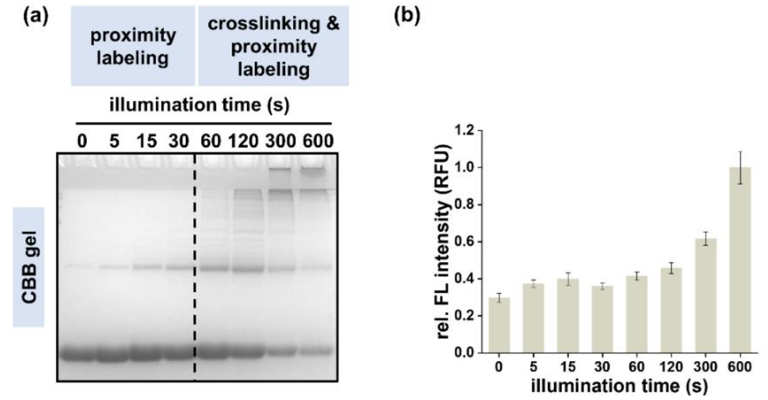

Figure S8. P5 induced proximity labeling of aggregated DHFR within 1 min and crosslinking happened with longer illumination time (refers to **Figure 3C**). (a) CBB gel corresponding to the FL gel in **Figure 3C**. (b) Quantification of dots experiments in **Figure 3C**. Protein concentration: 2.0 mg·mL^{-1}; P5 concentration: 2.0 μM; Propargyl amine concentration: 10.0 mM; Light intensity: 3 mW·cm^{-2}. Dots experiments were performed on nitrocellulose film and imaged using VISQUE InVivo Smart-LF bio-imaging system. Error bars: standard error (n = 3).

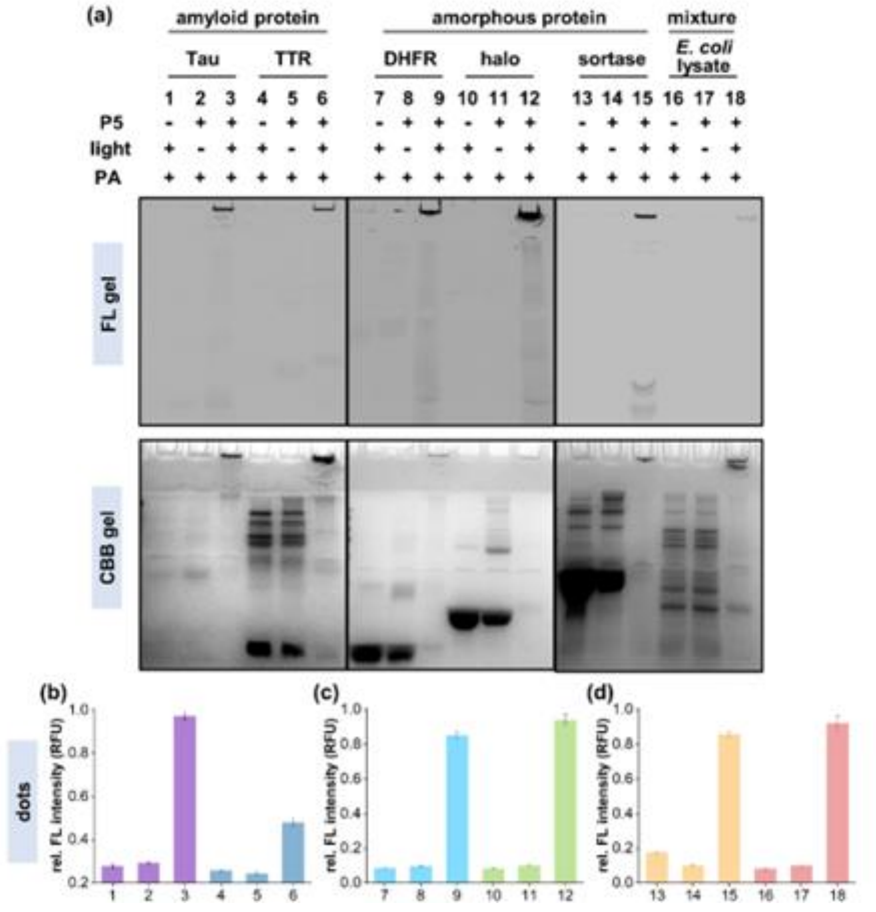

Figure S9. P5 generally proximity labeled and crosslinked different types of aggregated proteins and lysate upon photo illumination (refers to **Figure 3D**). (a) FL full gel and its corresponding CBB gel. (b), (c), and (d) Quantification of dots experiments in **Figure 3D**. Protein concentration: 2.0 mg·mL^{-1}; P5 concentration: 20.0 μM; Propargyl amine concentration: 10.0 mM; Light intensity: 25 mW·cm^{-2}. Dots experiments were performed on nitrocellulose film and imaged using VISQUE InVivo Smart-LF bio-imaging system. Error bars: standard error (n = 3).

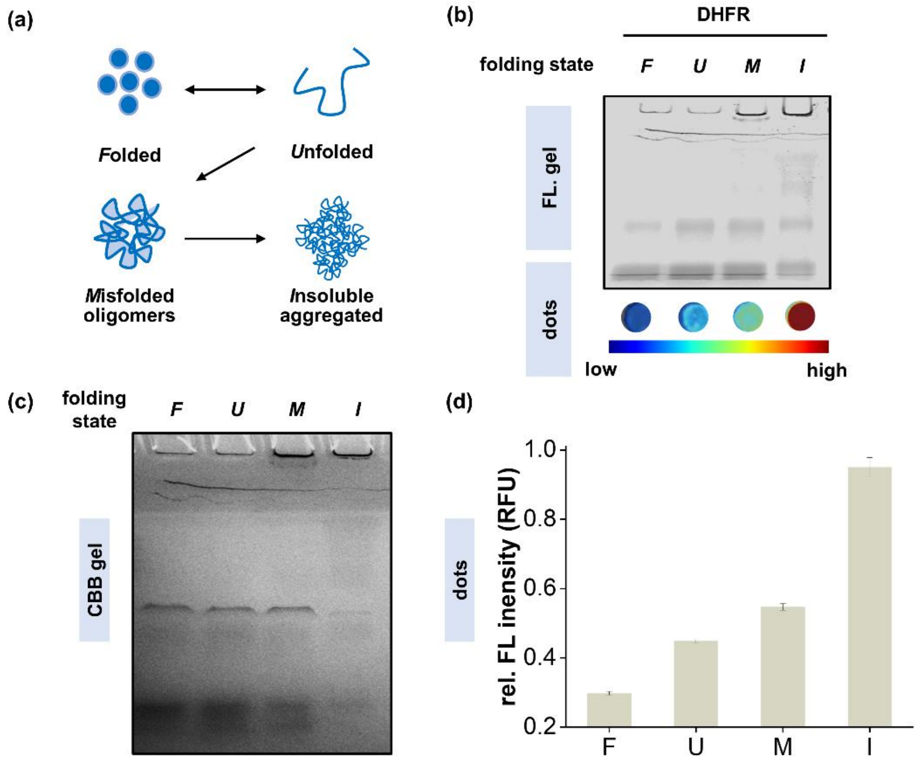

Figure S10. P5 started to label and crosslink proteins upon the formation of misfolded oligomers. *F*: folded proteins; *U*: unfolded proteins; *M*: misfolded oligomers; *I*: insoluble aggregates. (a) Scheme of protein aggregation process. (b) FL. Gel & dots and (c) CBB gel of P5 induced aggregated proteins labeling and crosslinking. (d) Quantification of dots experiments in (b). Protein concentration: 2.0 mg·mL^{-1}; P5 concentration: 20.0 µM; Propargylamine concentration: 10.0 mM; Light intensity: 25 mW·cm^{-2}. Dots experiments were performed on nitrocellulose film and imaged using VISQUE InVivo Smart-LF bio-imaging system. Error bars: standard error (n = 3).

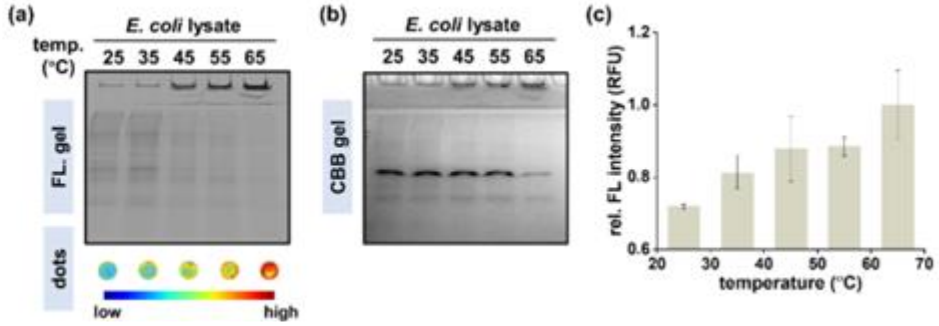

Figure S11. P5 induced *E. coli* lysates proximity labeling during its aggregation process. (a) FL gel and dots experiment for aggregated *E. coli* lysates proximity labeling and its corresponding (b) CBB gel and (c) quantification. *E. coli* lysate concentration: 2.0 mg·mL^{-1}; P5 concentration: 20.0 µM; Propargy lamine concentration: 10.0 mM; Light intensity: 25 mW·cm^{-2}. Dots experiments were performed on nitrocellulose film and imaged using VISQUE InVivo Smart-LF bio-imaging system. Error bars: standard error (n = 3).

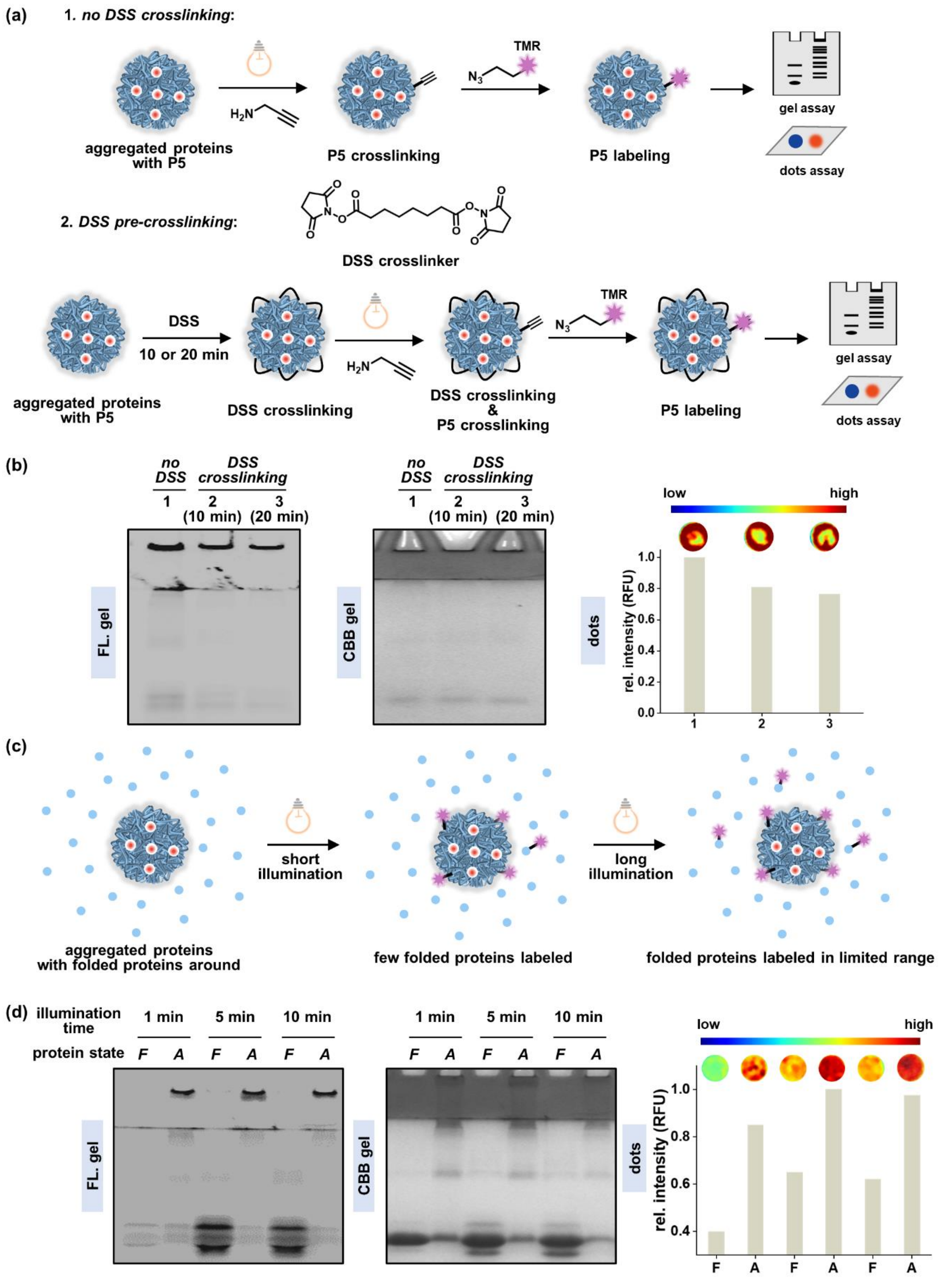

Figure S12. Photo-crosslinking of aggregated proteins and the short labeling range of ROS contribute to AggID's selectivity to aggregated proteins. (a) Scheme of validating crosslinking impact on labeling range and efficiency. Disuccinimidyl suberate (DSS) is a commercially available protein crosslinker to crosslink interacting lysines. (b) FL gel, CBB gel, and dots experiment to evaluate aggregated proteins labeling efficiency in the absence and presence of DSS. (c) Scheme of validating the short labeling range of ROS by measuring the labeling efficiency of aggregated proteins in the presence of folded proteins. (d) FL gel, CBB gel, and dots experiment for folded and aggregated proteins labeling under different illumination time. Protein concentration: 2.0 mg·mL^{-1}; P5 concentration: 20.0 μM; Substrate concentration: 10.0 mM; Light intensity: 25 mW·cm^{-2}. For DSS crosslinked aggregated proteins: 30 mM DSS was treated with aggregated proteins for 10 and 20 min. The quenching of DSS crosslinking effect was realized by the addition of 1.0 M Tris-HCl buffer (pH = 7.4).

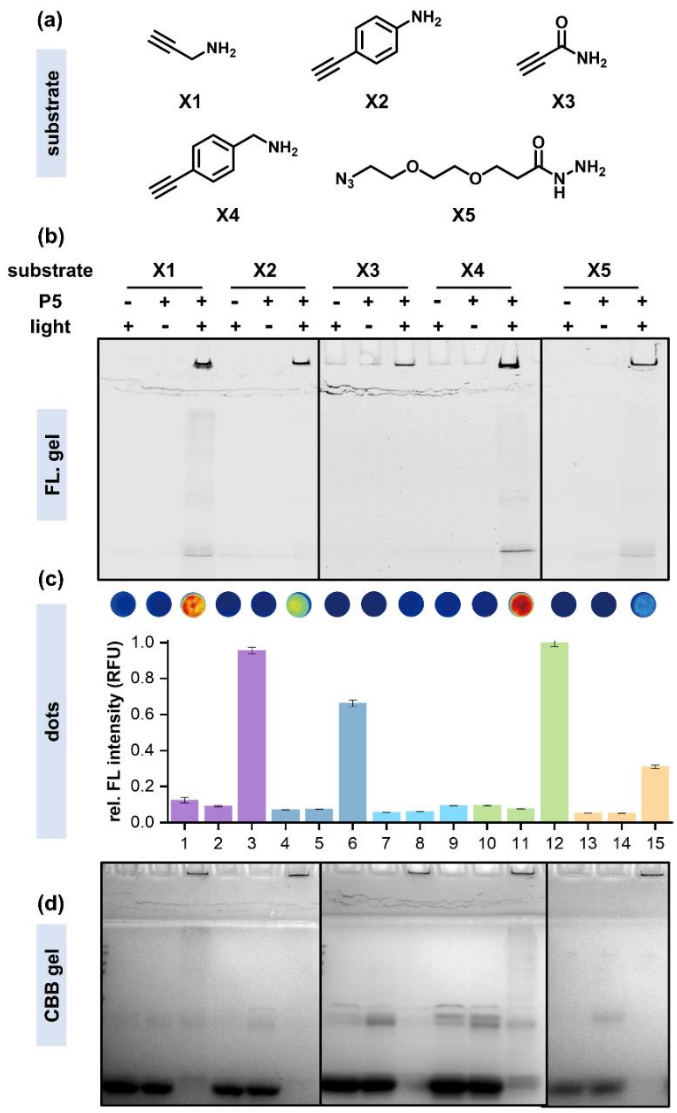

Figure S13. Benzylamine was the most ideal labeling substrate for P5 induced proximity labeling (refers to **Figure 3F**). (a) structures of different labeling substrates including aliphatic amine, aniline, benzylamine, amide, and hydrazide. (b) FL gel and dots experiment resuts of five different labeling substrate on P5 proximity labeling and its corresponding (c) quantification and (d) CBB gel to (b). Protein concentration: 2.0 mg·mL^{-1}; P5 concentration: 20.0 μM; Substrate concentration: 10.0 mM; Light intensity: 25 mW·cm^{-2}. Dots experiments were performed on nitrocellulose film and imaged using VISQUE InVivo Smart-LF bio-imaging system. Error bars: standard error (n = 3).

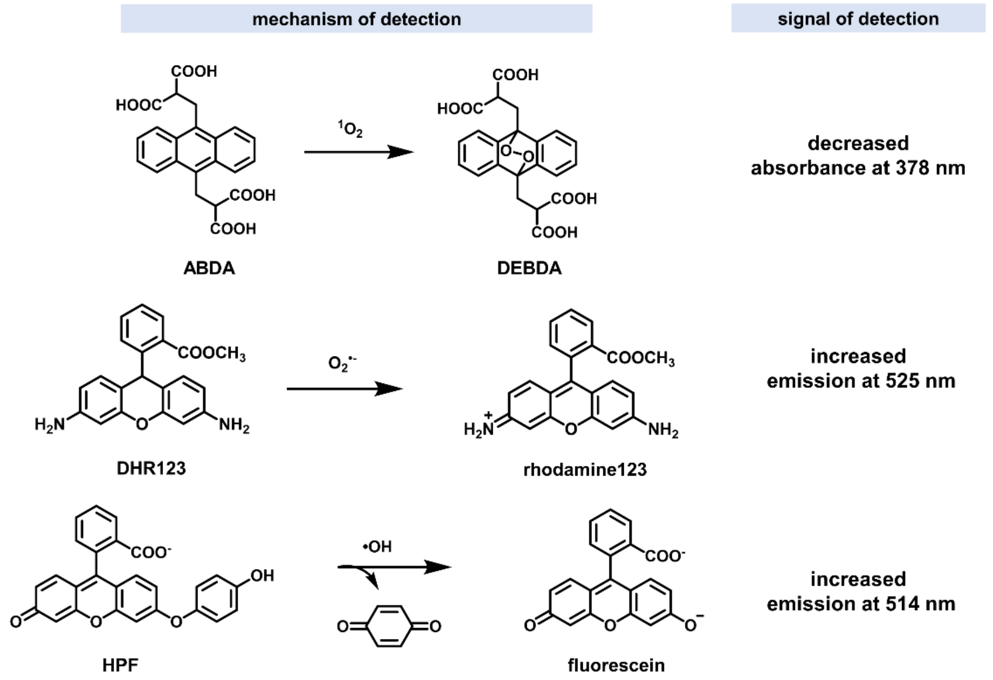

Figure S14. Sensing mechanism of ABDA, SOSG, DHR123, and HPF in detecting singlet oxygen, superoxide anions, and hydroxyl radical, respectively. For singlet oxygen detection, absorbance at 378 nm was collected. For superoxide anions detection, fluorescence emission at 525 nm was collected. For hydroxyl radicals detection, fluorescence emission at 514 nm was collected.

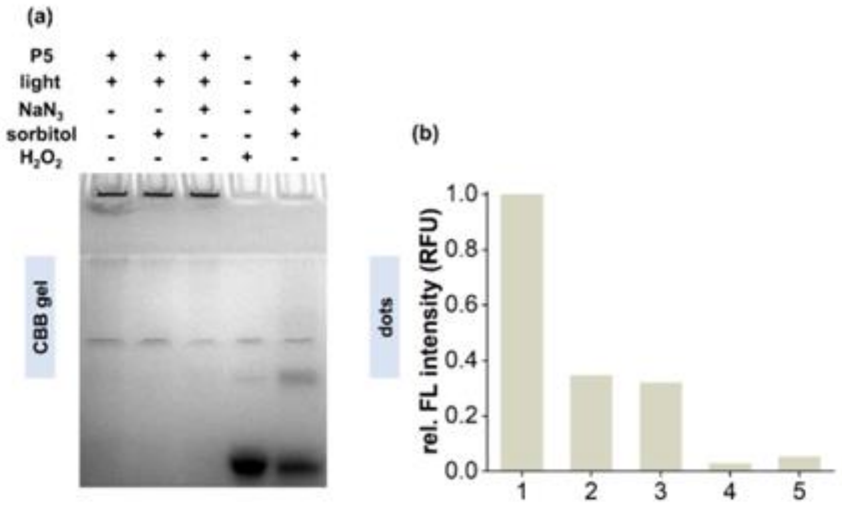

Figure S15. Both type I and type II ROS contribute to P5 induced proximity labeling (refers to **Figure 4E**). (a) CBB gel corresponding to the FL gel in **Figure 4E**. (b) Quantification of dots experiments in **Figure 4E**. Protein concentration: 2.0 mg·mL^{-1}; P5 concentration: 20.0 µM; Propargyl amine concentration: 10.0 mM; Light intensity: 25 mW·cm^{-2}; Illumination time: 30 min. NaN_3, sorbitol, and H_2O_2 concentration: 10.0 mM. Error bars: standard error (n = 3). Dots experiments were performed on nitrocellulose film and imaged using VISQUE InVivo Smart-LF bio-imaging system.

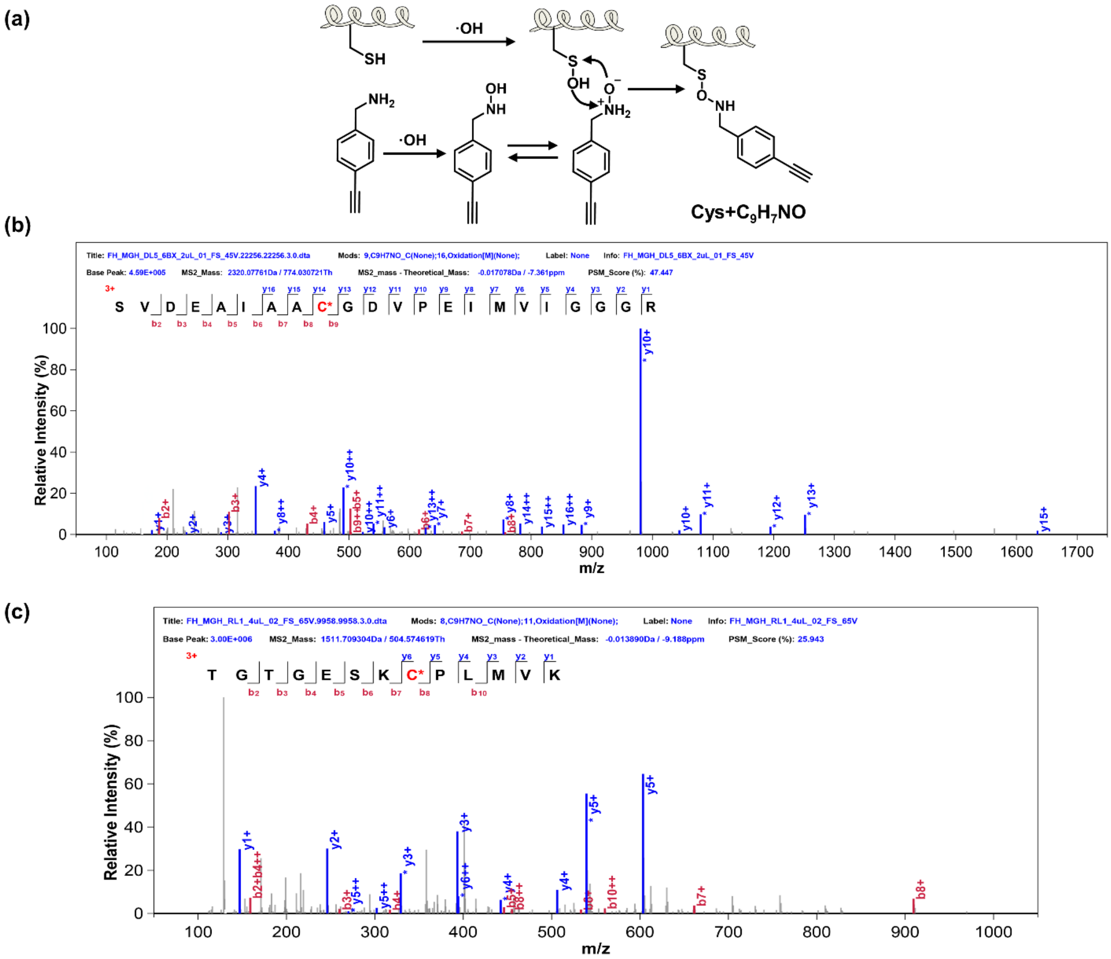

Figure S16. LC-MS/MS spectra of cysteine labeled with benzylamine. (a) Proposed labeling mechanism on cysteine (+C_9H_7NO) and corresponding identified labeling results on (b) WT-DHFR and (c) WT-TTR protein.[7]

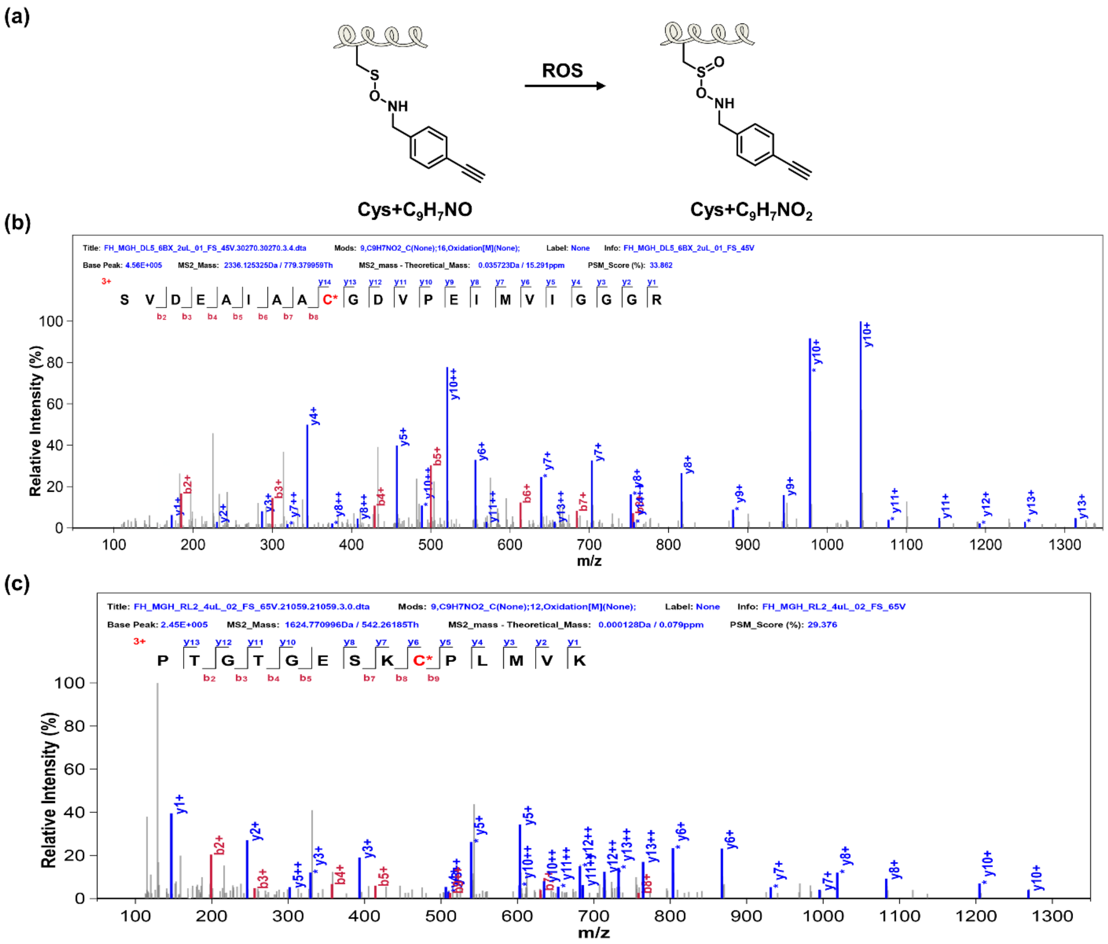

Figure S17. LC-MS/MS spectra of cysteine labeled with benzylamine. (a) Proposed labeling mechanism on cysteine (+$C_9H_7NO_2$) and corresponding identified labeling results on (b) WT-DHFR and (c) WT-TTR protein.[7]

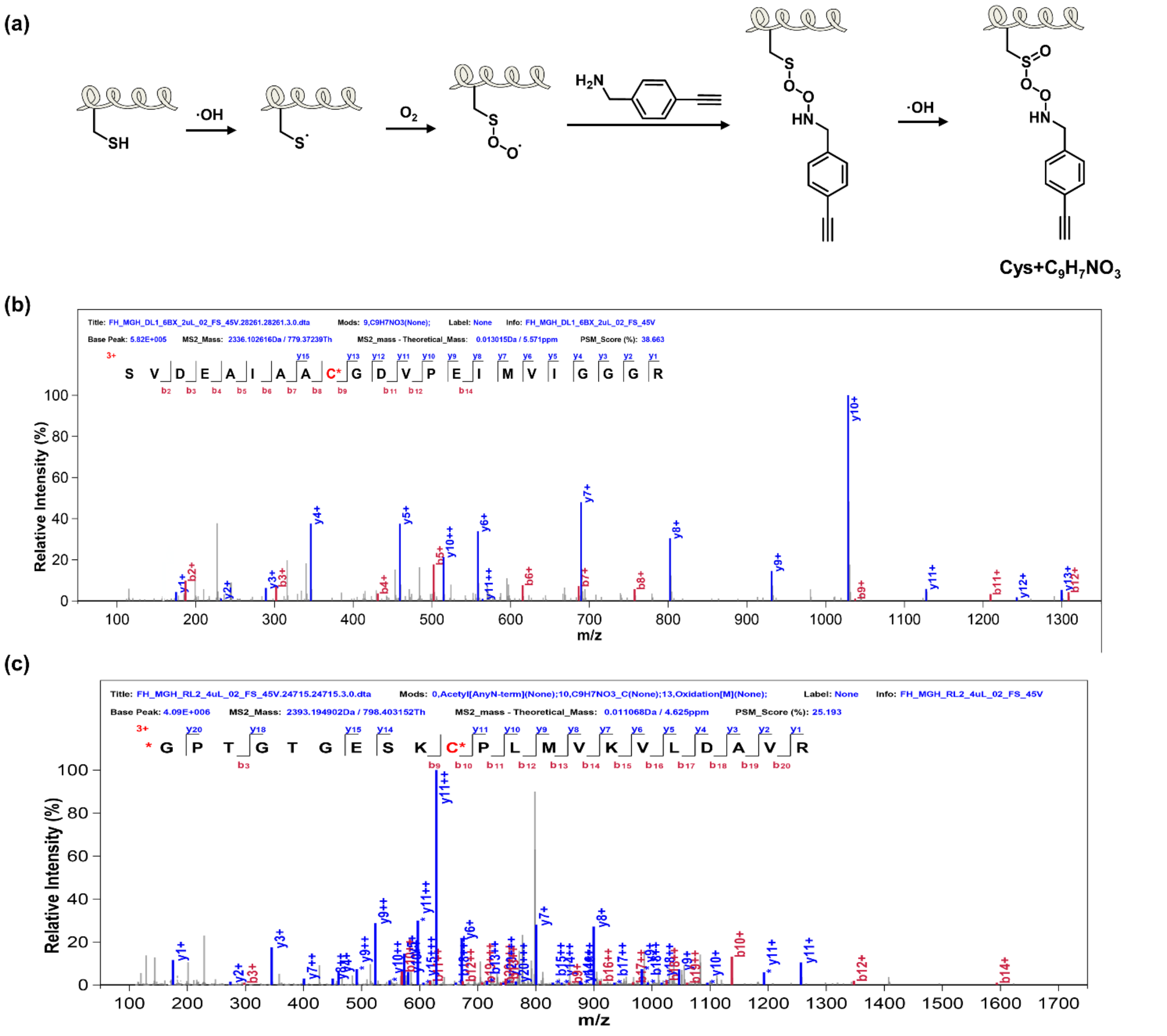

Figure S18. LC-MS/MS spectra of cysteine labeled with benzylamine. (a) Proposed labeling mechanism on cysteine (+$C_9H_7NO_3$) and corresponding identified labeling results on (b) WT-DHFR and (c) WT-TTR protein.[7]

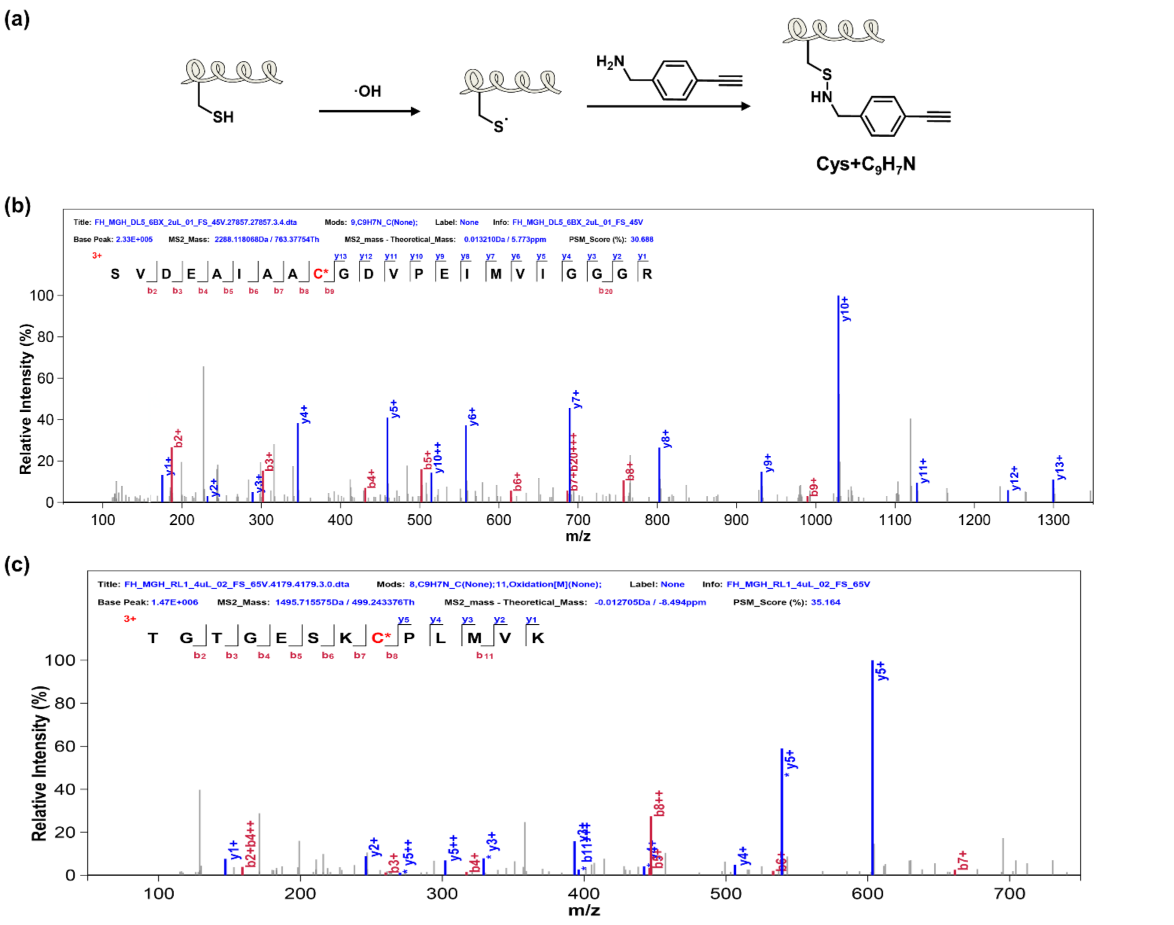

Figure S19. LC-MS/MS spectra of cysteine labeled with benzylamine. (a) Proposed labeling mechanism on cysteine (+C_9H_7N) and corresponding identified labeling results on (b) WT-DHFR and (c) WT-TTR protein.[8]

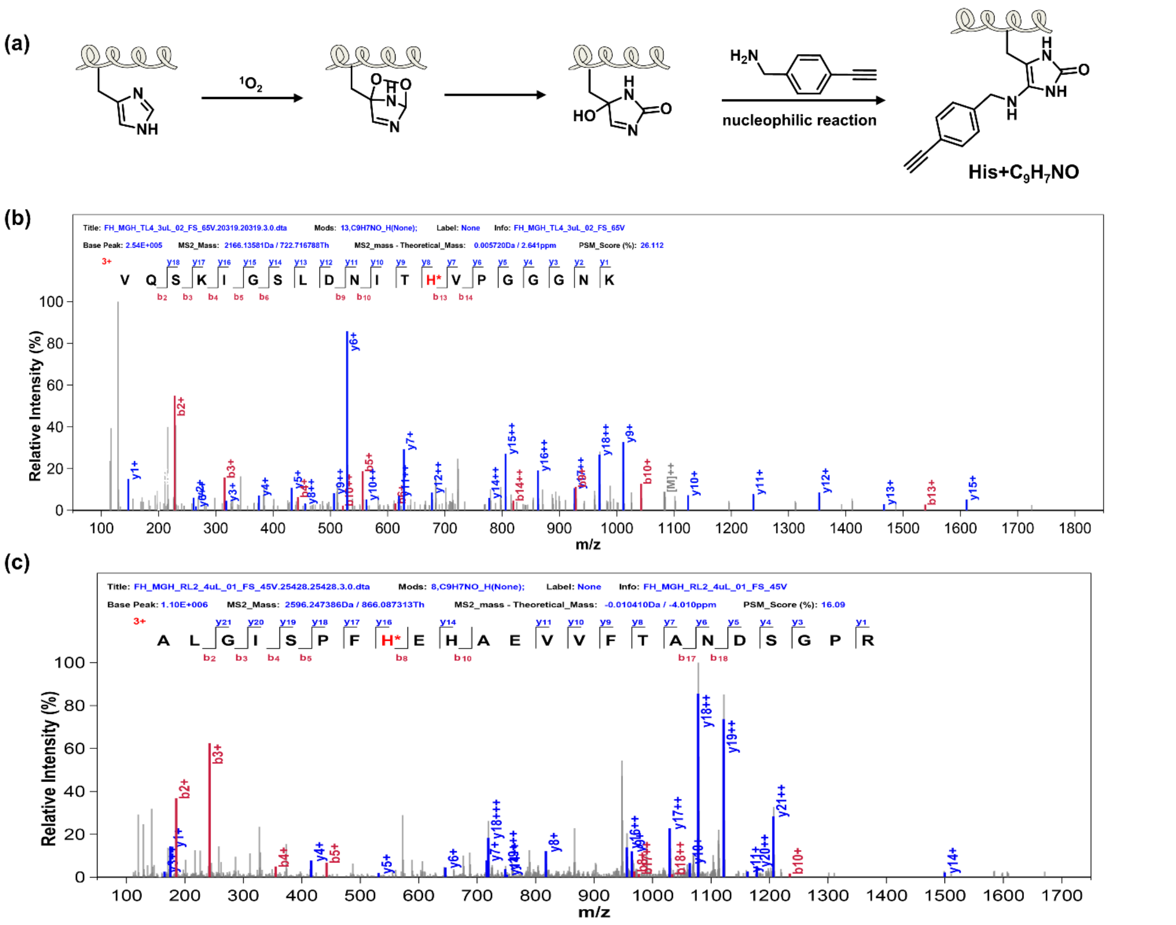

Figure S20. LC-MS/MS spectra of histidine labeled with benzylamine. (a) Proposed labeling mechanism on histidine (+C_9H_7NO) and corresponding identified labeling results on (b) Tau-K18 and (c) WT-TTR protein.[8]

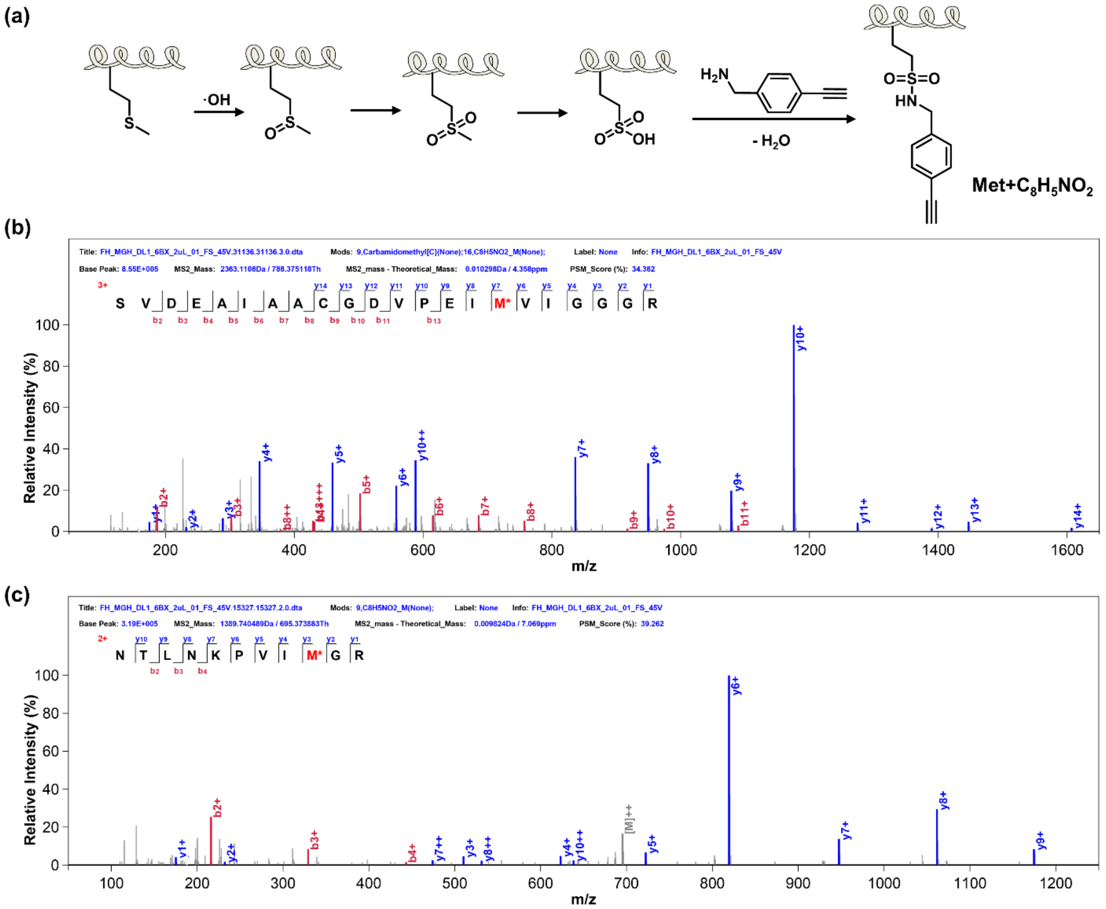

Figure S21. LC-MS/MS spectra of methionine labeled with benzylamine. (a) Proposed labeling mechanism on methionine (+$C_8H_5NO_2$) and corresponding identified labeling results on (b) and (c) WT-DHFR protein.[8]

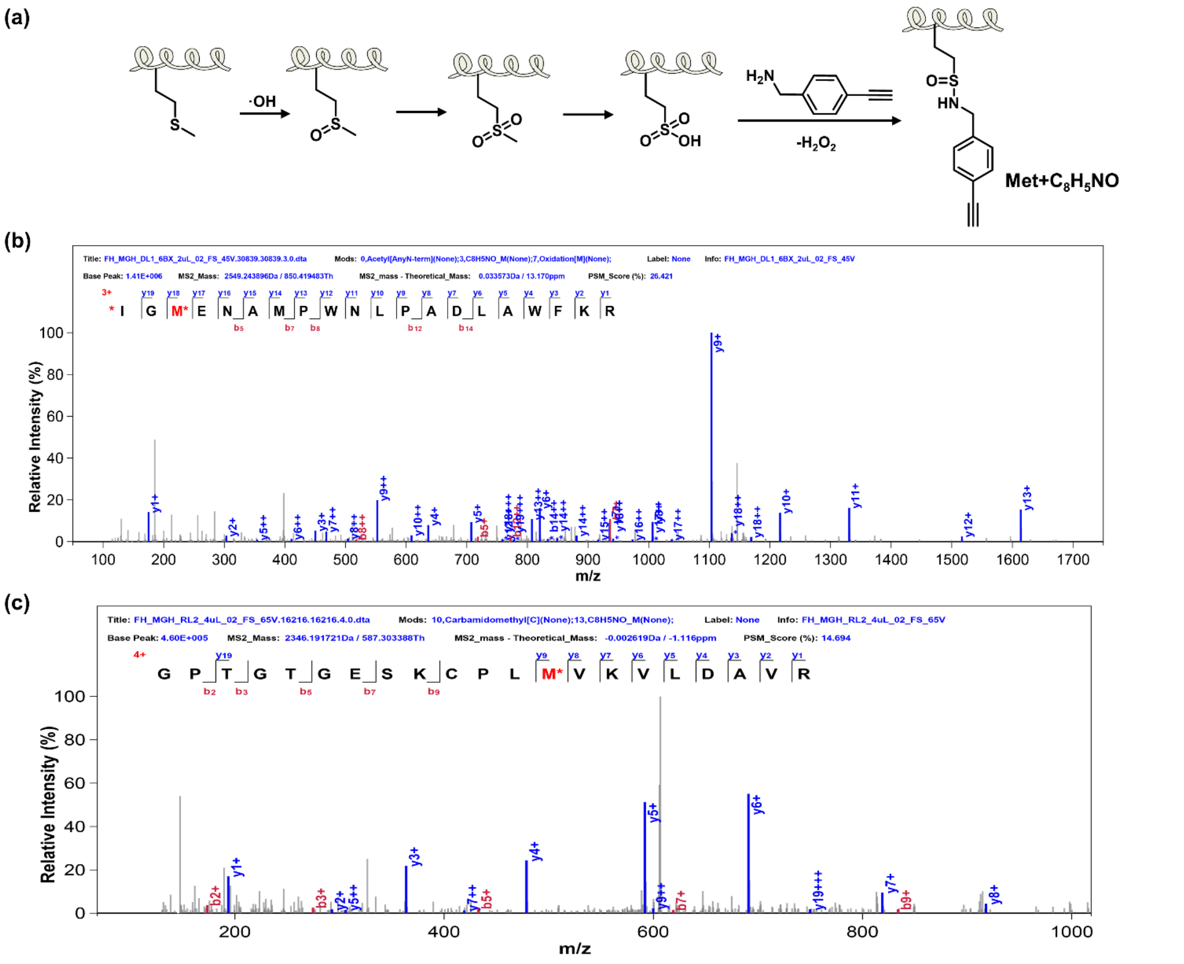

Figure S22. LC-MS/MS spectra of methionine labeled with benzylamine. (a) Proposed labeling mechanism on methionine (+C_8H_5NO) and corresponding identified labeling results on (b) WT-DHFR and (c) WT-TTR protein.[8]

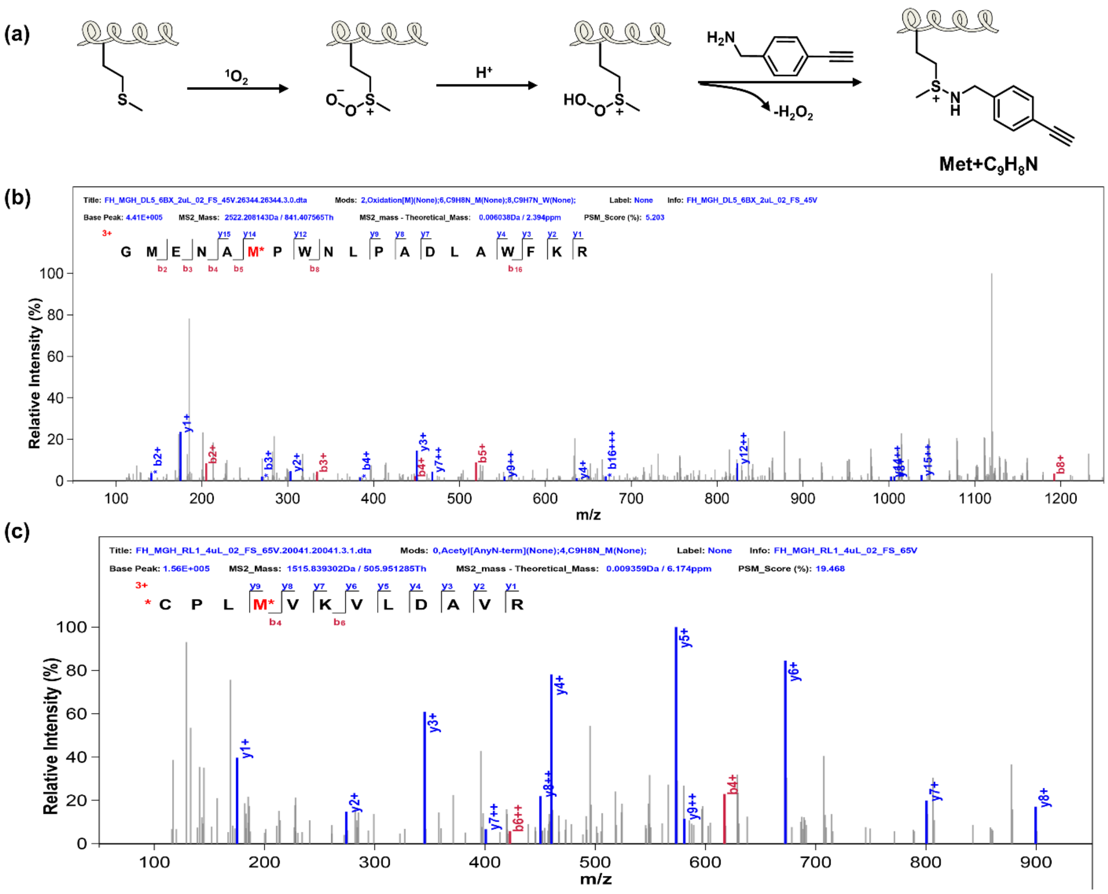

Figure S23. LC-MS/MS spectra of methionine labeled with benzylamine. (a) Proposed labeling mechanism on methionine (+C_9H_8N) and corresponding identified labeling results on (b) WT-DHFR and (c) WT-TTR protein.[8]

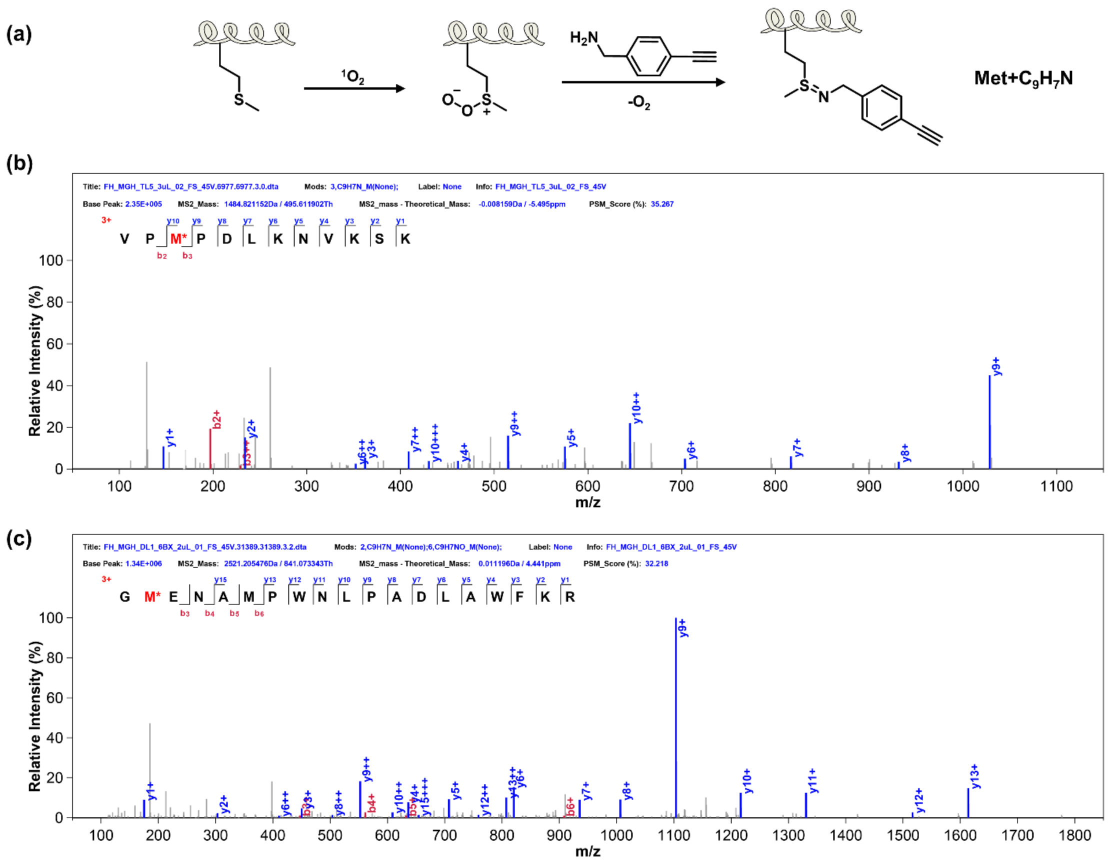

Figure S24. LC-MS/MS spectra of methionine labeled with benzylamine. (a) Proposed labeling mechanism on methionine (+C_9H_7N) and corresponding identified labeling results on (b) Tau-K18 and (c) WT-DHFR protein.

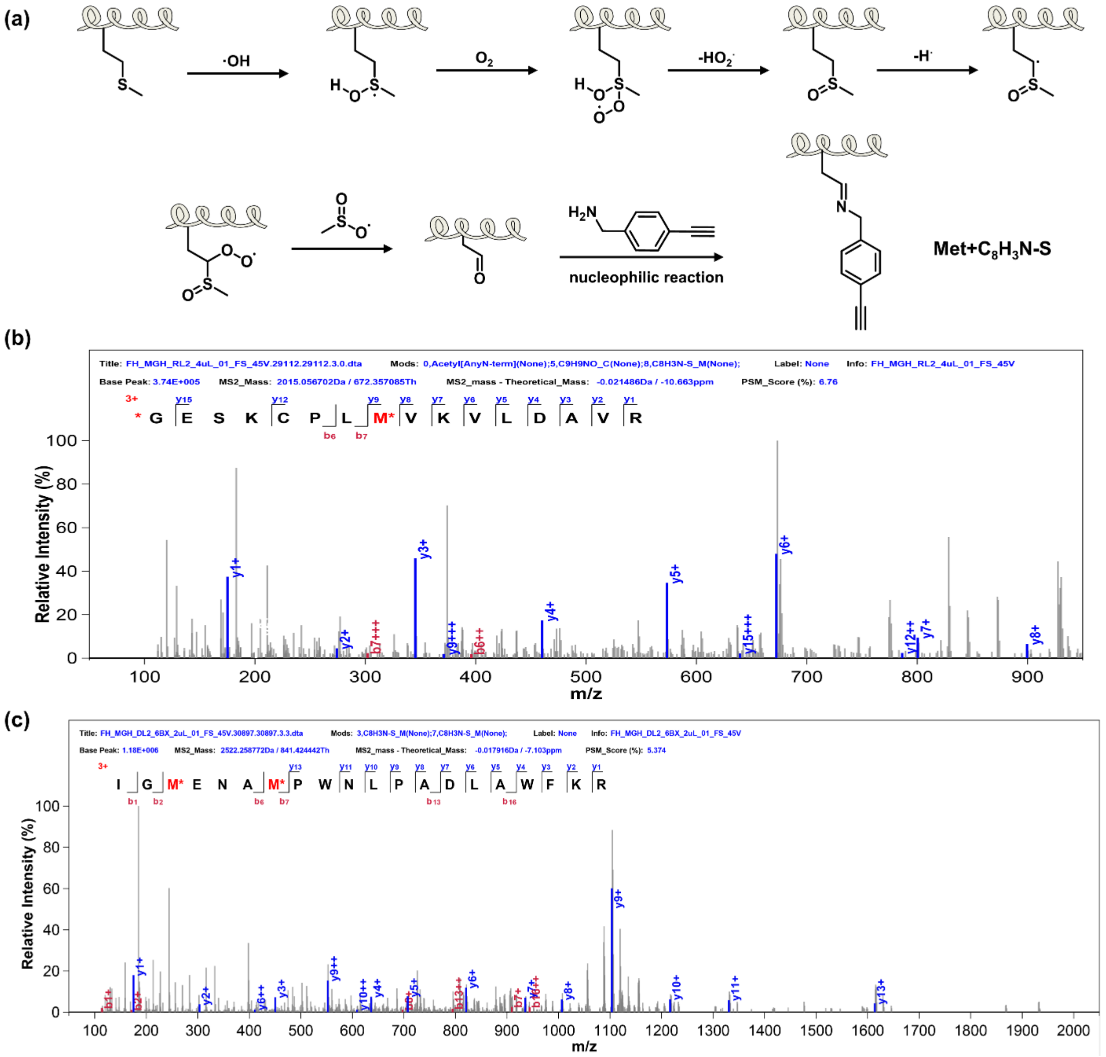

Figure S25. LC-MS/MS spectra of methionine labeled with benzylamine. (a) Proposed labeling mechanism on methionine (+C_8H_3N-S) and corresponding identified labeling results on (b) WT-TTR and (c) WT-DHFR protein.[9]

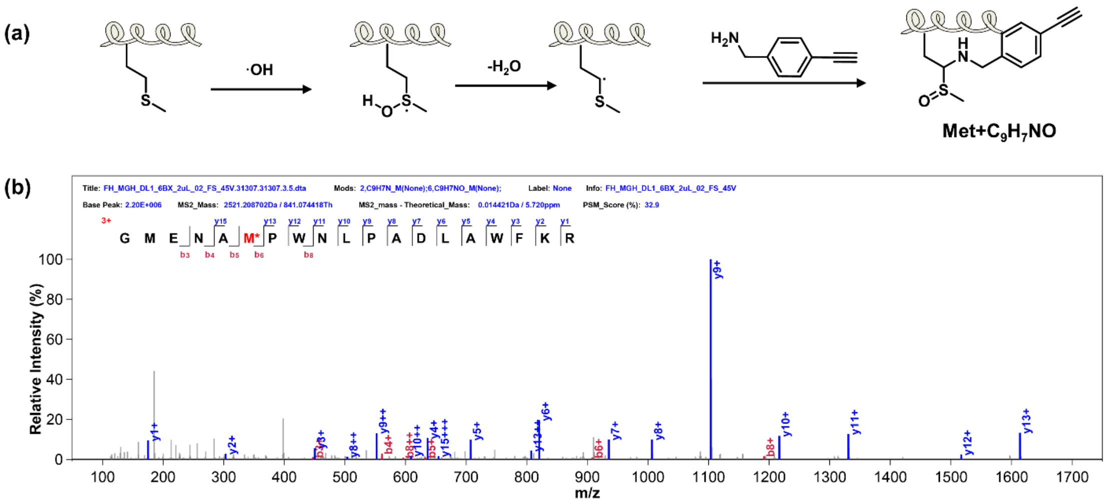

Figure S26. LC-MS/MS spectra of methionine labeled with benzylamine. (a) Proposed labeling mechanism on methionine (+C_9H_7NO) and corresponding identified labeling results on (b) WT-DHFR.

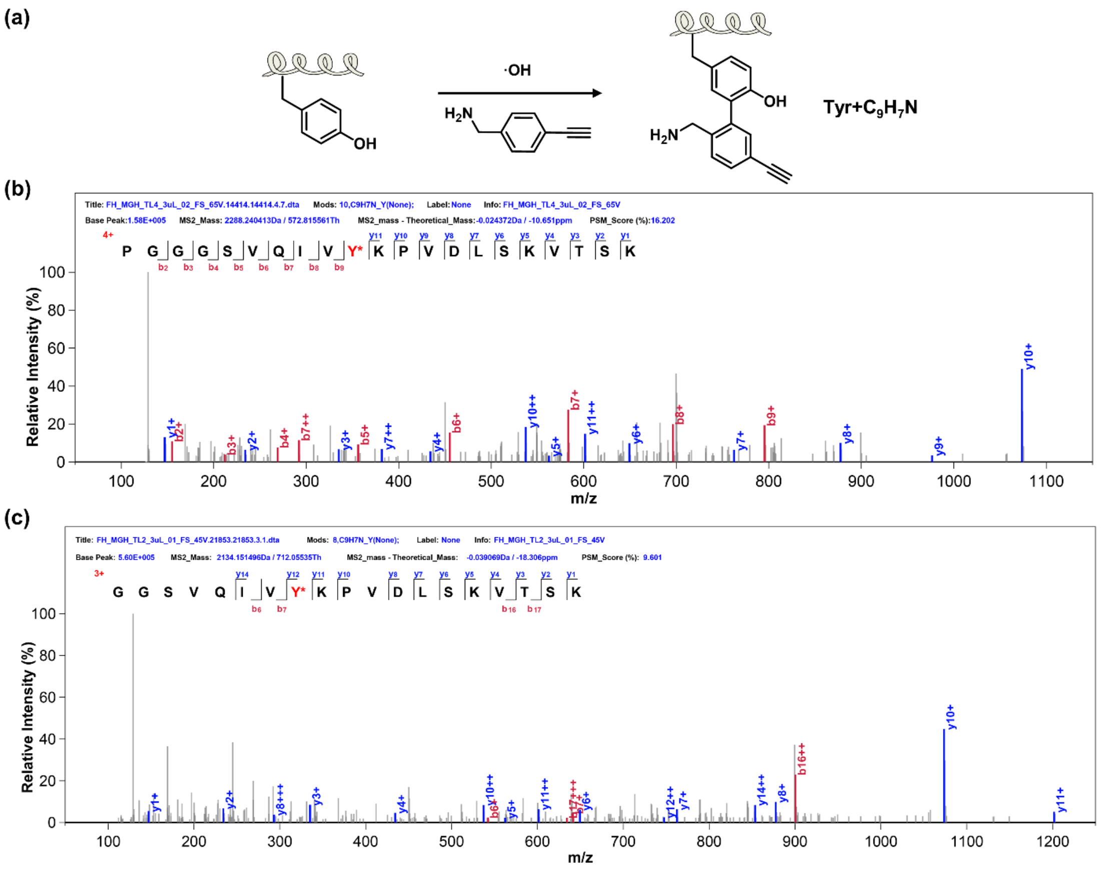

Figure S27. LC-MS/MS spectra of tyrosine labeled with benzylamine. (a) Proposed labeling mechanism on tyrosine (+C_9H_7N) and corresponding identified labeling results on (b) and (c) Tau-K18 protein.[10]

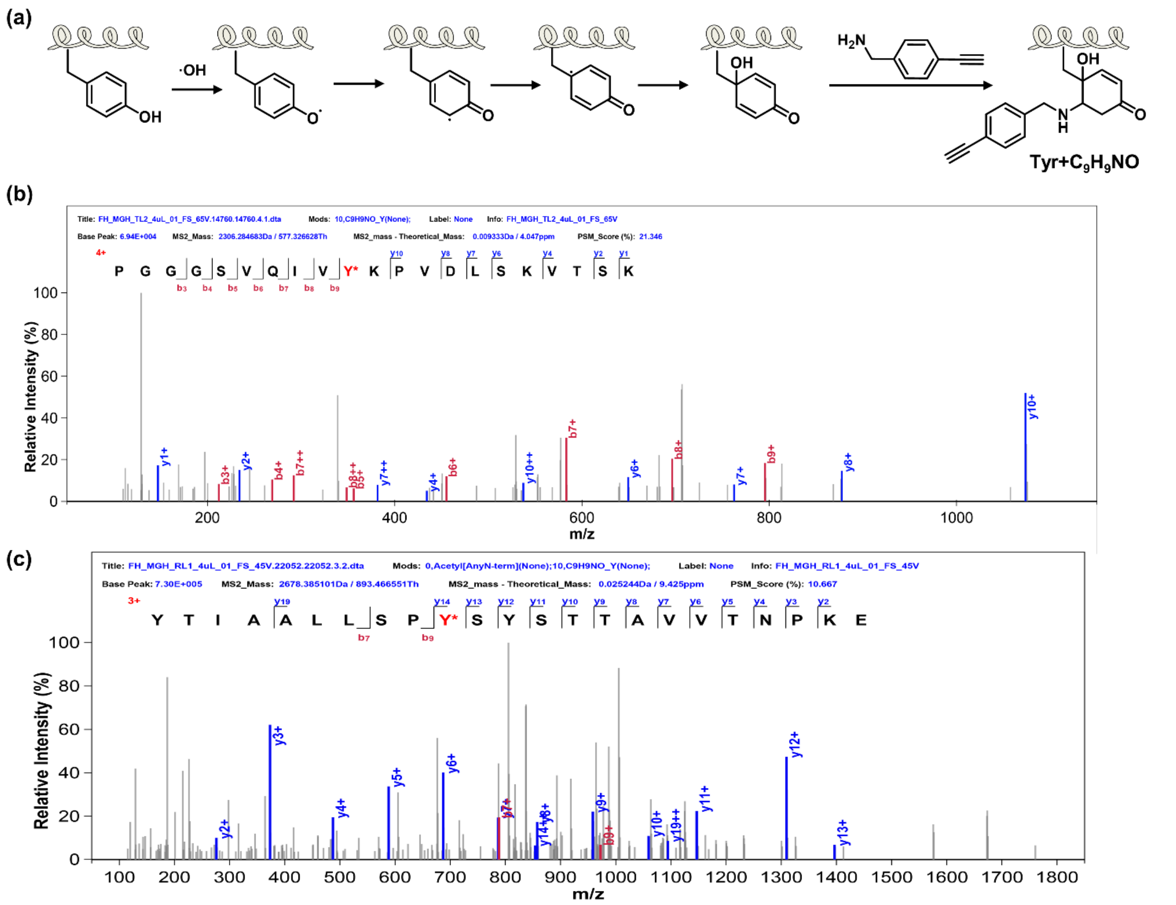

Figure S28. LC-MS/MS spectra of tyrosine labeled with benzylamine. (a) Proposed labeling mechanism on tyrosine (+C_9H_9NO) and corresponding identified labeling results on (b) Tau-K18 and (c) WT-TTR protein.[10]

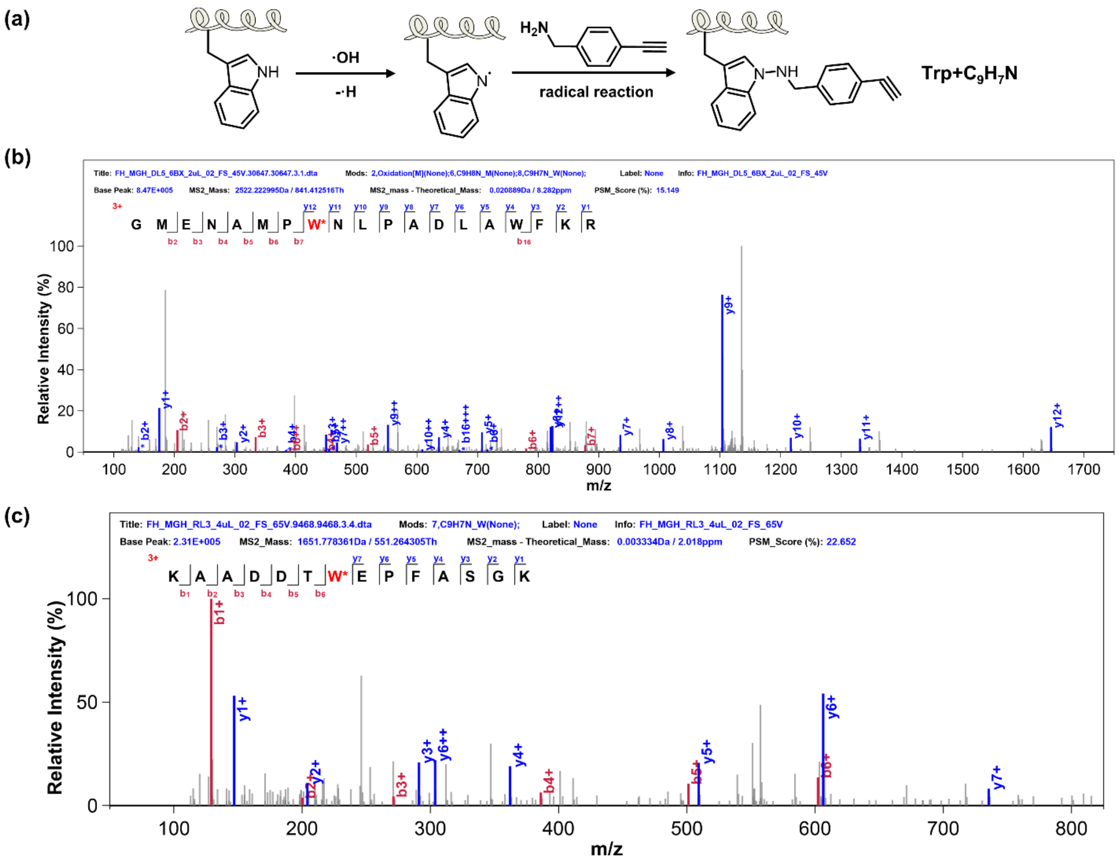

Figure S29. LC-MS/MS spectra of tryptophan labeled with benzylamine. (a) Proposed labeling mechanism on tryptophan (+C_9H_7N) and corresponding identified labeling results on (b) WT-DHFR and (c) WT-TTR protein.[11]

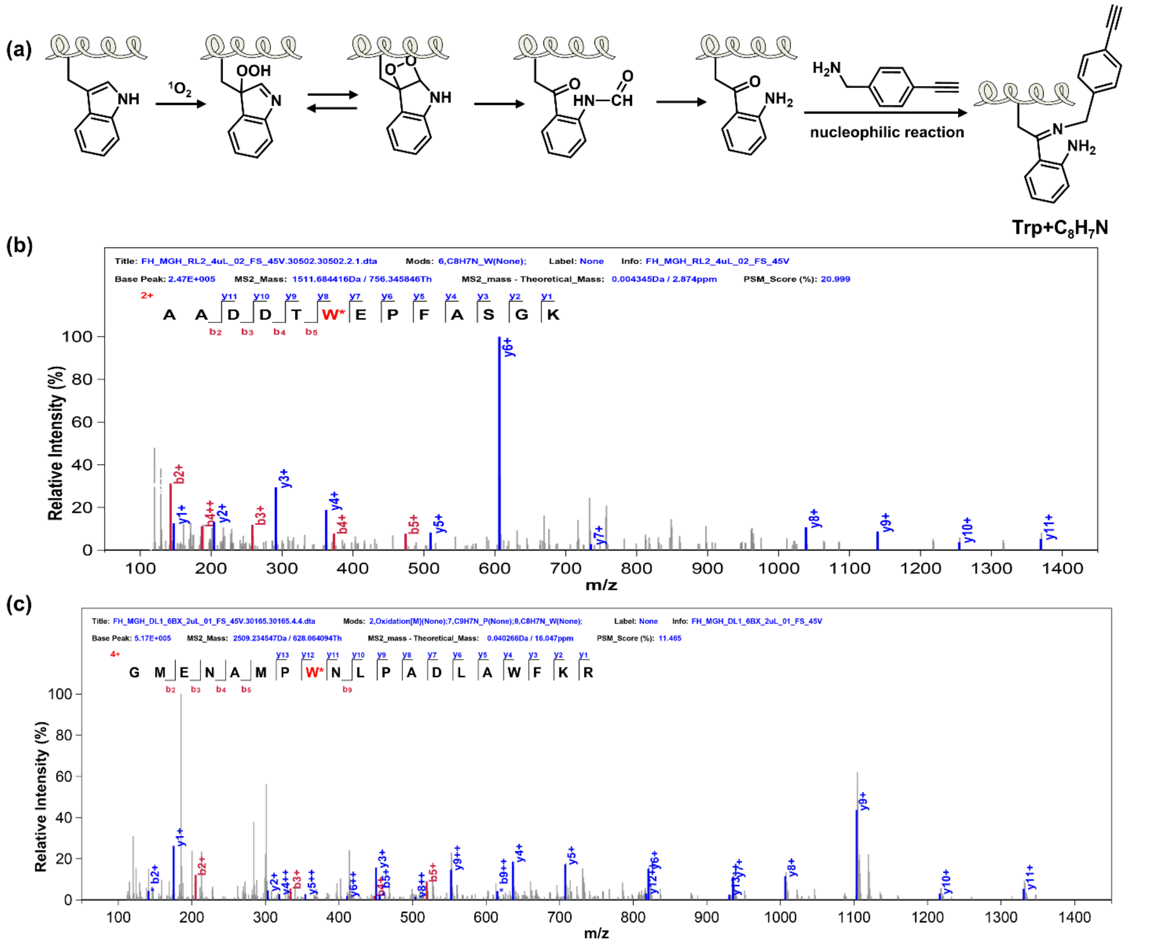

Figure S30. LC-MS/MS spectra of tryptophan labeled with benzylamine. (a) Proposed labeling mechanism on tryptophan (+C_8H_7N) and corresponding identified labeling results on (b) WT-TTR and (c) WT-DHFR protein.[12]

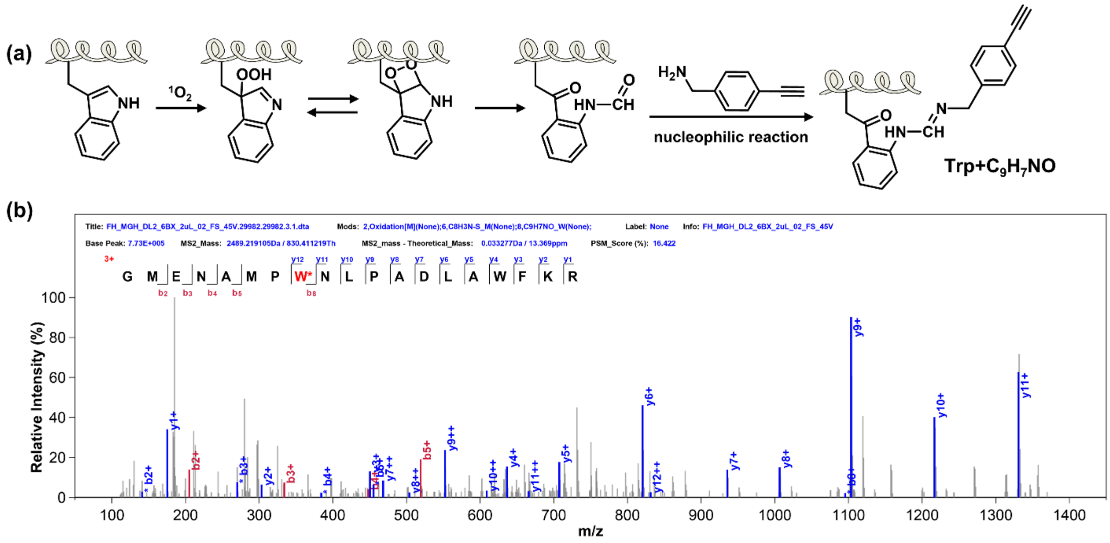

Figure S31. LC-MS/MS spectra of tryptophan labeled with benzylamine. (a) Proposed labeling mechanism on tryptophan (+C_9H_7NO) and corresponding identified labeling results on (b) WT-DHFR.[12]

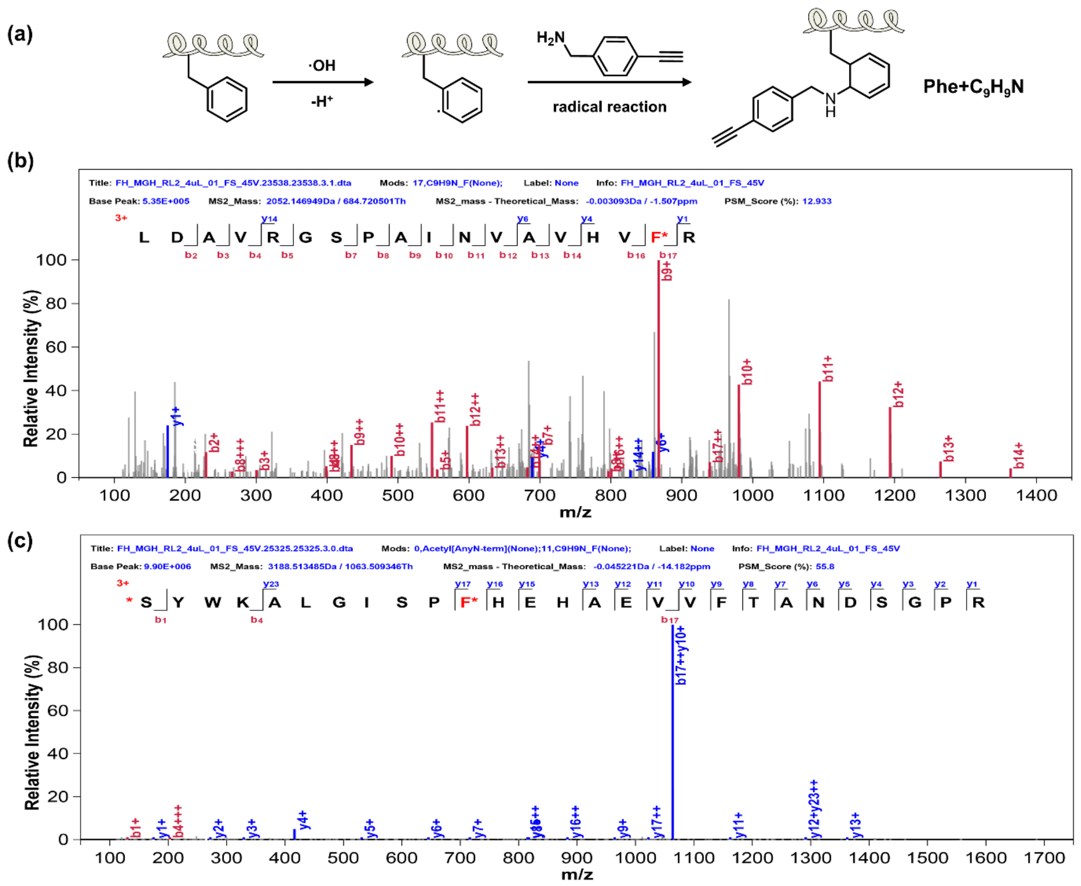

Figure S32. LC-MS/MS spectra of phenylanine labeled with benzylamine. (a) Proposed labeling mechanism on phenylanine (+C_9H_9N) and corresponding identified labeling results on (b) and (c) WT-TTR protein.[8]

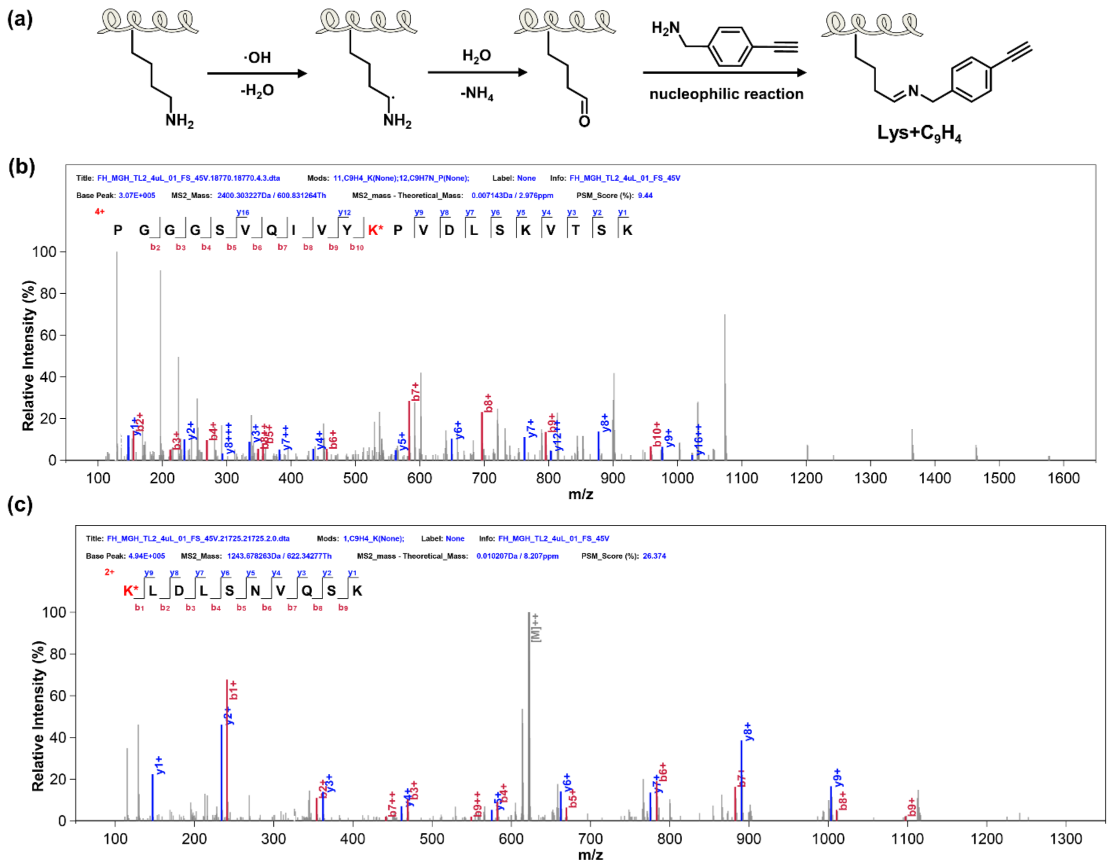

Figure S33. LC-MS/MS spectra of lysine labeled with benzylamine. (a) Proposed labeling mechanism on lysine (+C_9H_4) and corresponding identified labeling results on (b) and (c) Tau-K18 protein.[9]

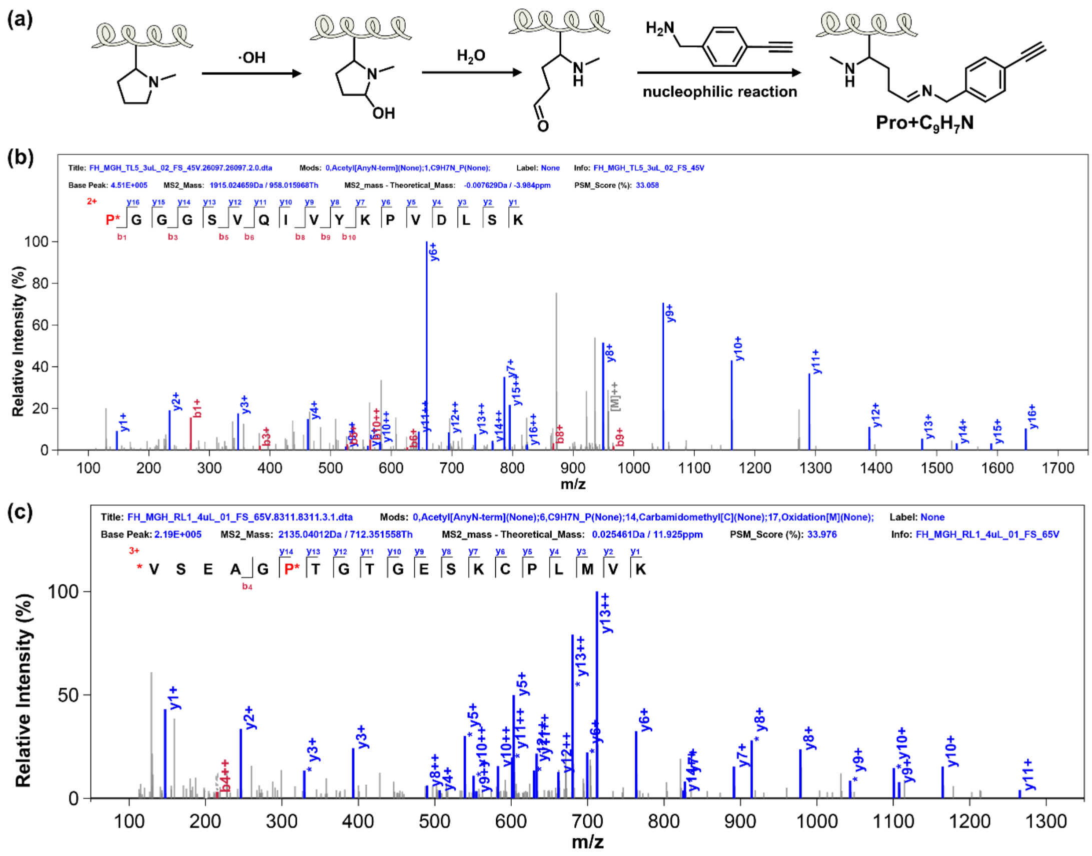

Figure S34. LC-MS/MS spectra of proline labeled with benzylamine. (a) Proposed labeling mechanism on proline (+C_9H_7N) and corresponding identified labeling results on (b) Tau-K18 and (c) WT-TTR protein.[9]

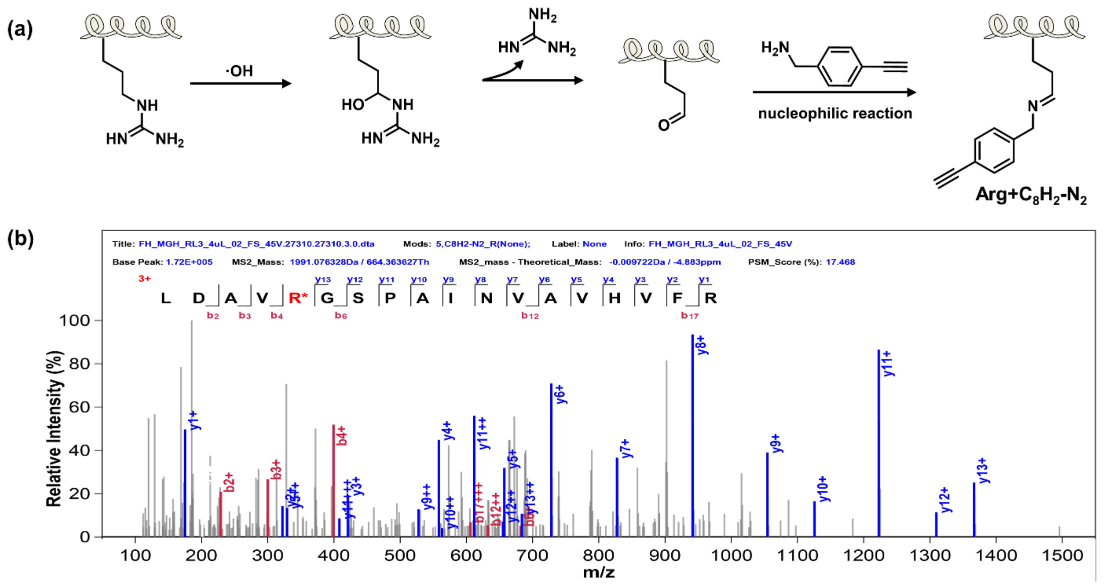

Figure S35. LC-MS/MS spectra of arginine labeled with benzylamine. (a) Proposed labeling mechanism on arginine (+C_8H_2-N_2) and corresponding identified labeling results on (b) WT-TTR protein.[9]

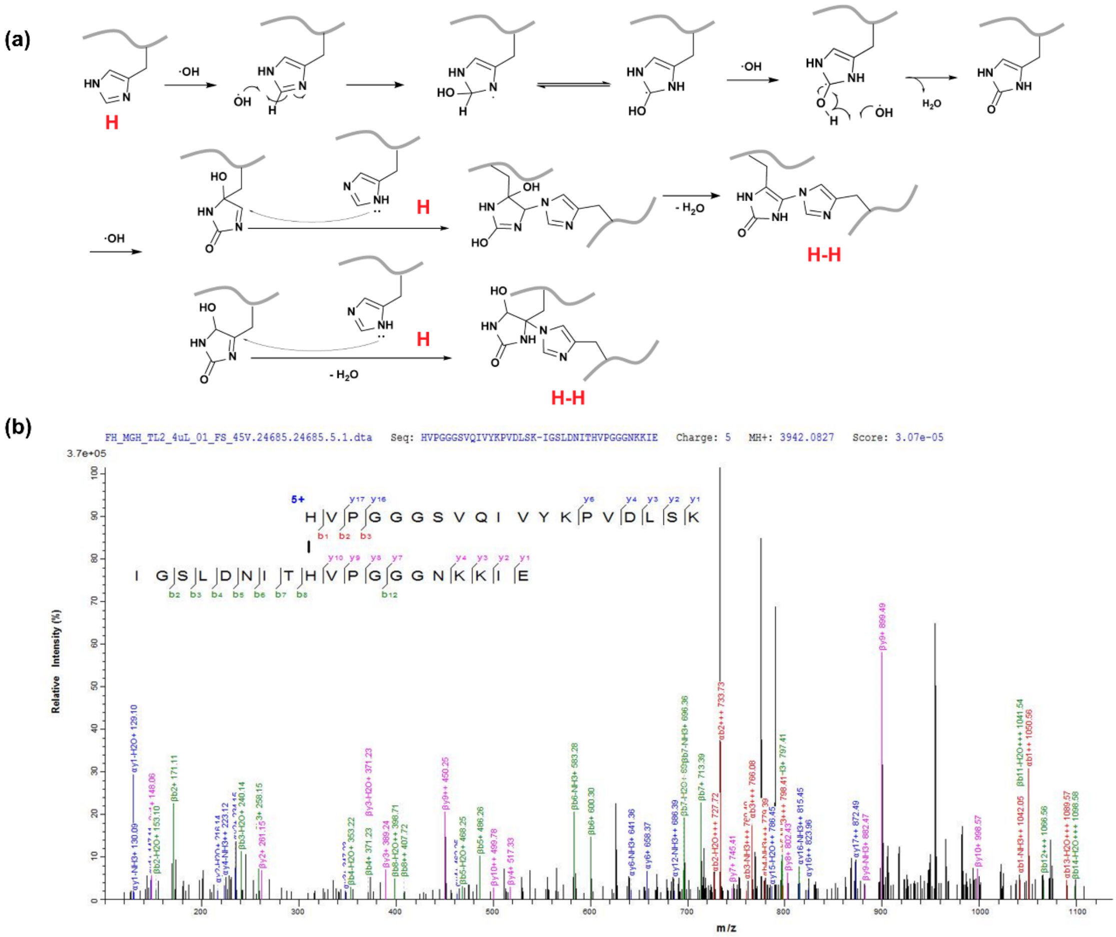

Figure S36. LC-MS/MS spectra of histidine-histidine crosslinking. (a) Proposed histidine-histidine crosslinking mechanism and (b) corresponding identified spectra results.

(a)

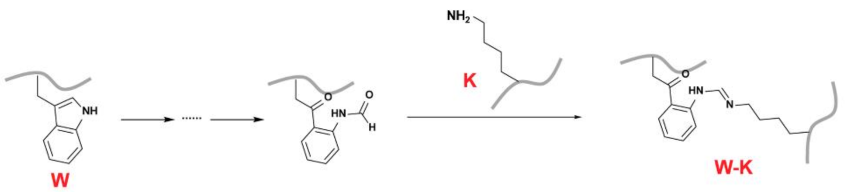

(b)

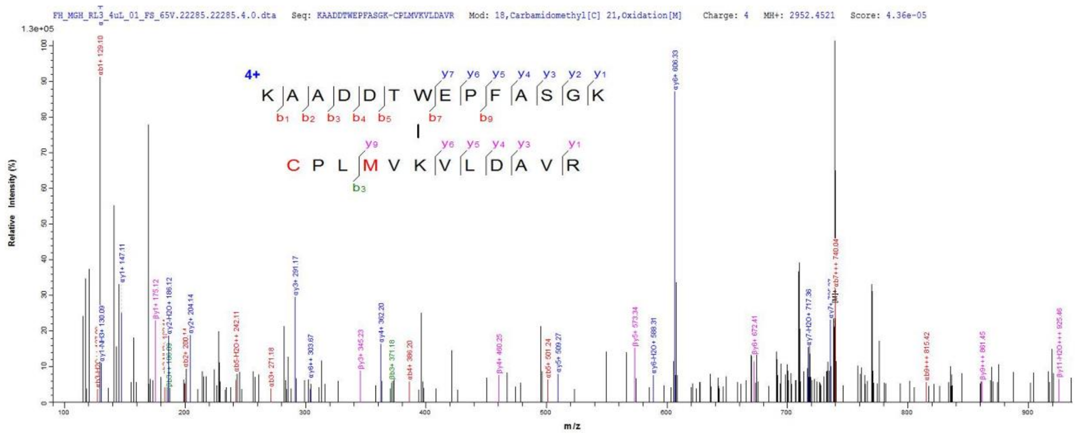

Figure S37. LC-MS/MS spectra of tryptophan-lysine crosslinking. (a) Proposed tryptophan-lysine crosslinking mechanism and (b) corresponding identified spectra results.

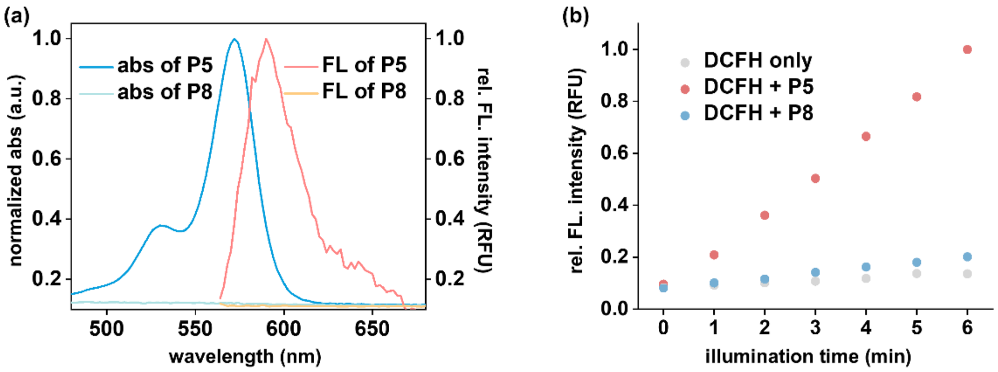

Figure S38. Acetylation of P5 masked its singlet fluorescence and triplet phorosensitizing properties. (a) absorpscence and fluorescence spertra of P5 and its acetylated counterparts P8. (b) ROS production rate of P5 and its acetylated counterparts P8. Error bars: standard error (n = 3).

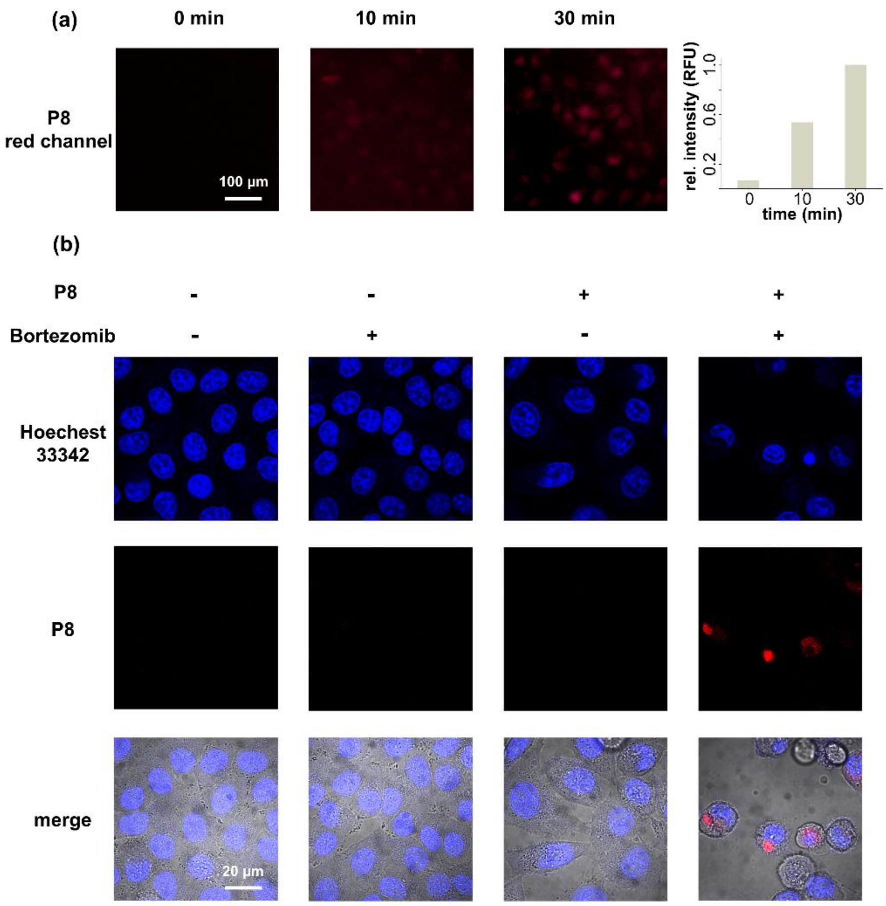

Figure S39. P8 was able to enter HeLa cells and bind aggregated proteome within 30 min. Stress of HeLa cells was induced by Bortezomib (0.8 µM). The concentration of P8 used to image aggregated proteome was 5.0 µM. Hoechst 33342 was used to stain nucleus. Nuclear staining fluorescence was visualized using violet laser (405 nm). Fluorescence of P8 was visualized using green HeNe laser (543 nm).

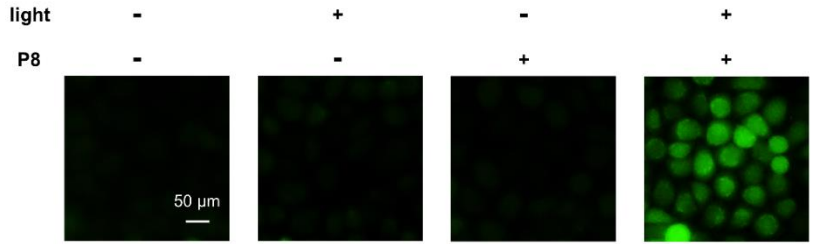

Figure S40. P8 (5.0 μM) generated ROS in live cells under the illumination of white light (25 mW·cm^{-2}) for 10 min. ROS production in cells was detected using DCFH-DA assay kit.

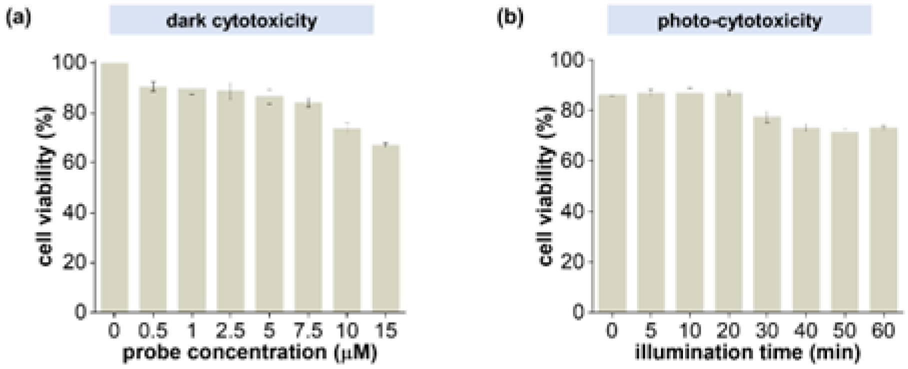

Figure S41. P8 showed satisfying dark toxicity (a) and photo-toxicity (b) under limited illumination time. In photo-toxicity assay and following imaging experiments, P8 probe concentration used was 5.0 µM. Cell viability was measured using MTT assay in HeLa cells. Error bars: standard error (n = 3).

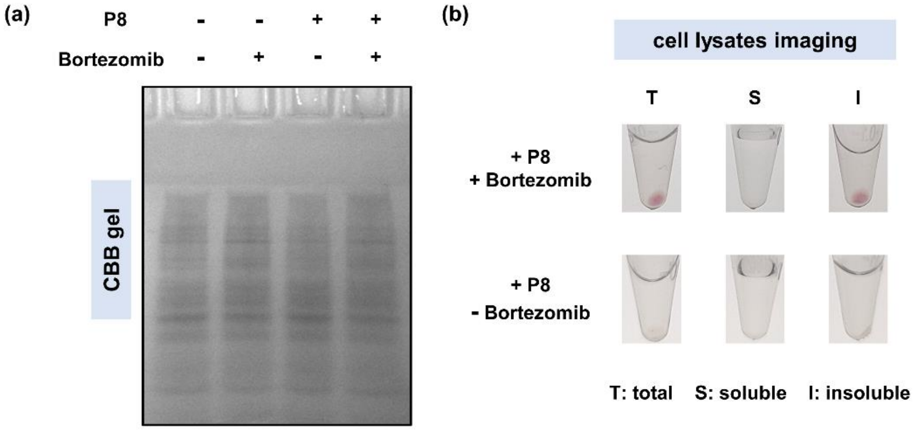

Figure S42. P8 selectively photo labeled aggregated proteome in stressed cell. (a) CBB gel refers to **Figure 5C. Figure 5C** showed that P8 (5.0 μM) efficiently labeled aggregated proteome in Bortezomib (0.8 μM) induced stressed cells under white light illumination (10 mW·cm^{-2}). The illumination time was 20 min. (b) P8 selectively bound to insoluble aggregated proteome over soluble ones. T: total proteins; S: soluble proteins; I: insoluble proteins.

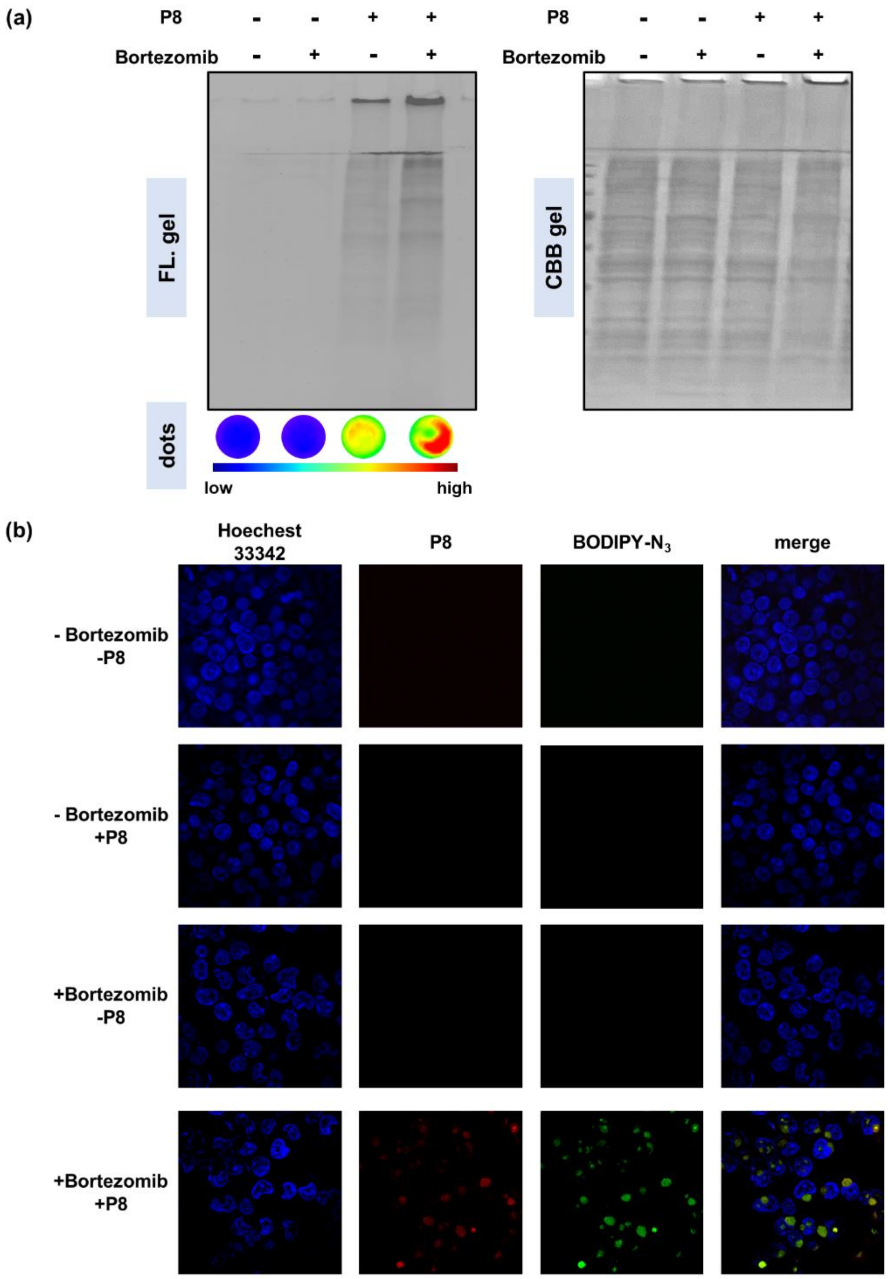

Figure S43. P8 selectively photo labeled aggregated proteome in stressed HEK-293T cells over non-stressed cells. (a) Gel results for the fluorescence labeling and its corresponding CBB staining results. (b) The proximity labeling region (green fluorescence) co-localized well with the aggregated proteome stained by P8 (red fluorescence). The green fluorescence signal of proximity labeling was from conjugation with BODIPY-N_3. [P8]: 5.0 µM; [Bortezomib]: 0.8 µM; Light intensity: 10 mW·cm^{-2}; Illumination time: 20 min.

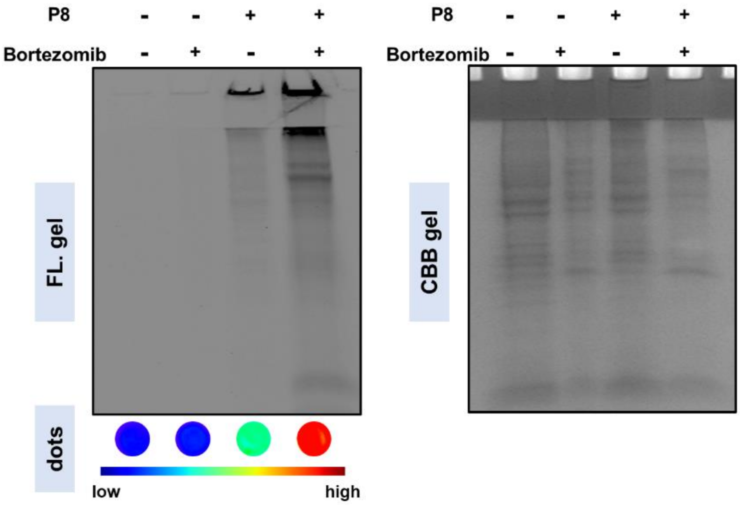

Figure S44. P8 selectively photo labeled aggregated proteome in stressed immune-related RAW 246.7 cells over non-stressed cells. [P8]: 5.0 µM; [Bortezomib]: 0.8 µM; Light intensity: 10 mW·cm^{-2}; Illumination time: 20 min.

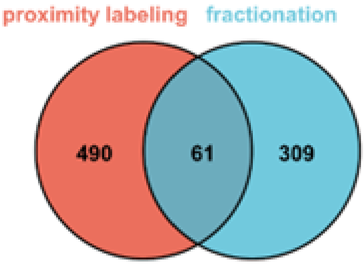

Figure S45. Venn diagram of protein IDs comparing P8-based proximity labeling method (AggID) and traditional fractionation method (PXD044948, and PXD037226).[13]

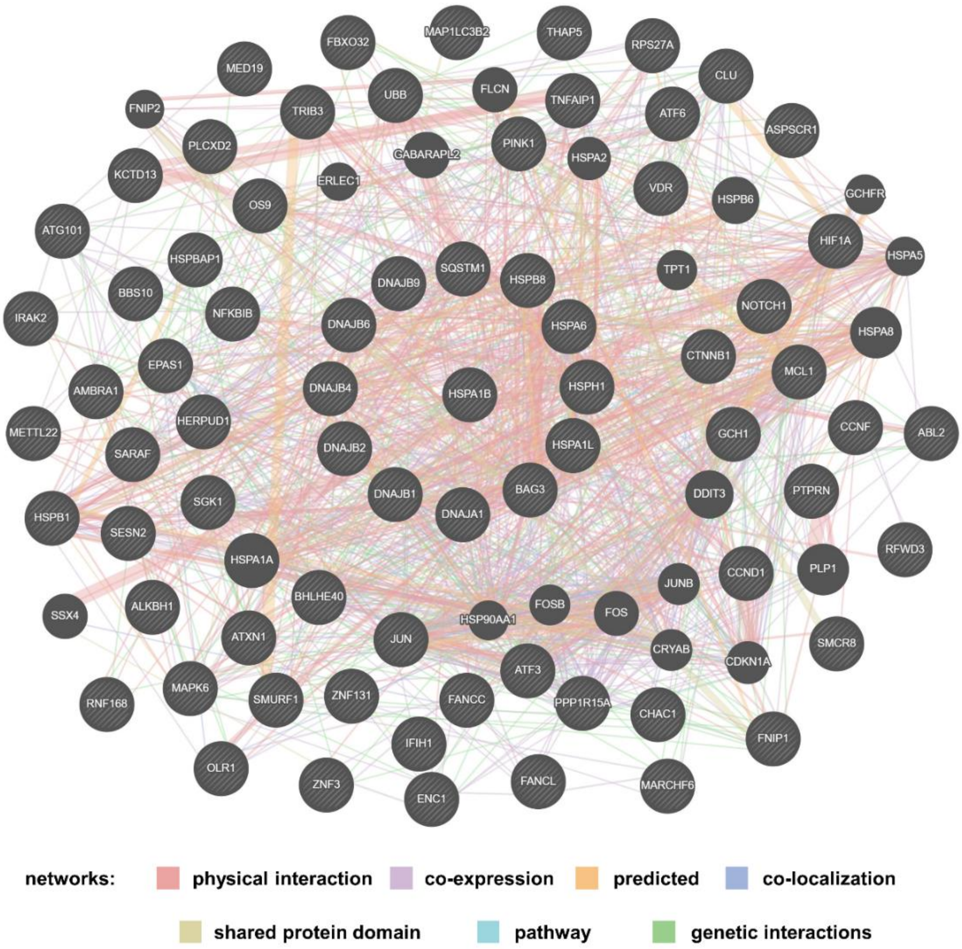

Figure S46. HSP70(HSPA1B) interaction netwok of identified up-regulated proteins.[14]

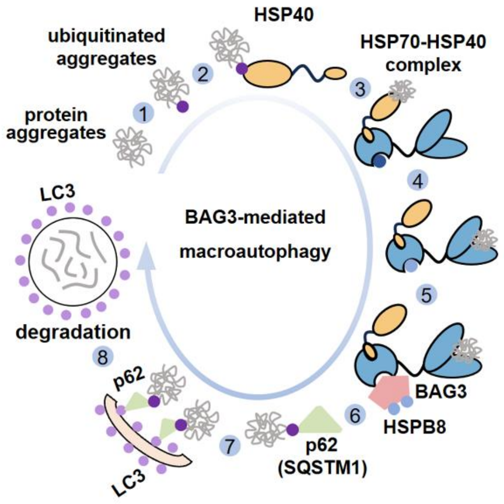

Figure S47. Reported model of BAG3-mediated macroautophagy degradation for aggregated proteins. In stressed cells, (1) misfolded or aggregated proteins are firstly ubiquitinated and (2) then recruited by co-chaperone HSP40. (3) Aggregates-HSP40 complex next bind to the NBD region of HSP70 and (4) further transfer aggregates to the SBD region. (5) A multi-chaperone complex consisting of HSP70, HSP40, HSPB8, and BAG3 is formed in preparation of aggregates degradation. BAG3 induces the selective macroautophagy of the aggregates by complexing with (6) macroautophagy receptor protein p62 that interacts simultaneously with both the aggregates as well as (7) the autophagosome membrane-associated protein LC3. (8) The aggregates are finally degraded by the autophagic system.[15]

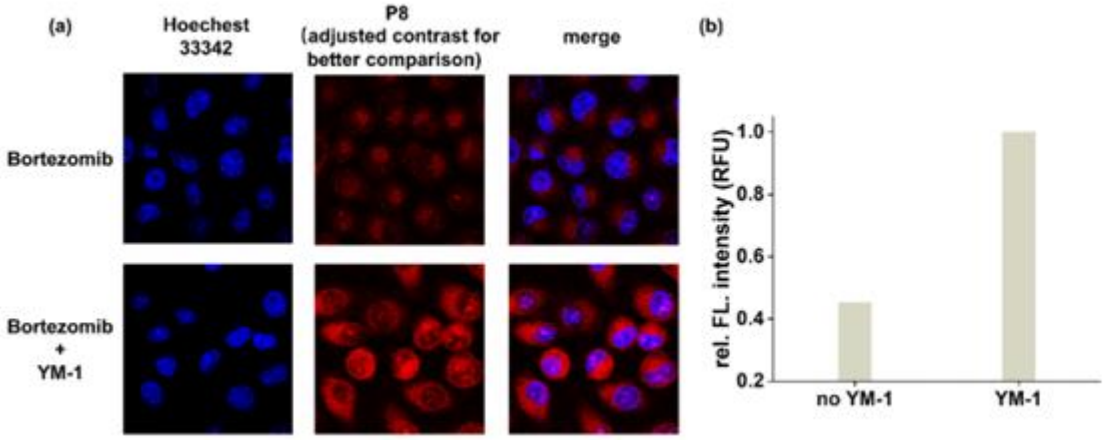

Figure S48. Protein aggregation was exaggerated upon treating with the HSP70-BAG3 inhibitor, YM-1. HeLa cells were subjected to treatment with Bortezomib (0.8 μM) and P8 (5.0 μM) with or without YM-1 (1.0 μM). YM-1 is a small molecule inhibitor that targets autophagy by disrupting the HSP70-BAG3 interaction, resulting in impaired regulation of misfolded and aggregated proteins.[16] These results support our claim that BAG3-mediated autophagic degradation was involved in handling the aggregation induced by proteasome inhibition.

Table S1. Summary of cLogP and binding efficiency of probes P1-P7.

number	name	cLogP	binding efficiency (%)
P1	Ru complex scaffold	NA	1.3±0.9
P2	coumarin scaffold	4.081	12.4±2. 6
P3	BODIPY scaffold	5.028	37.6±4.5
P4	riboflavin scaffold	-0.0029	3.3±5.2
P5	Rose Bengal scaffold	9.04	96.1±0.4
P6	methylene blue scaffold	0.9495	14.4±0.9
P7	cyanine scaffold	0.425	72.6±0.3

Table S2. Summary of proteins photo labeling types hypotheses. (**refers to Figure 4F**)

ranking	amino acids residues	labeling type	ΔM (Da)	spectrum counts
1	Lysine (K)	$+C_9H_4$	+112.0313	3383
2	Proline (P)	$+C_9H_7N$	+129.0578	2821
3	Tryptophan (W)	$+C_9H_7N$	+129.0578	1156
4	Tryptophan (W)	$+C_8H_7N$	+117.0578	1129
5	Arginine (R)	$+C_8H_2$-N_2	+70.0095	1068
6	Methionine (M)	$+C_8H_3N$-S	+81.0545	1057
7	Histidine (H)	$+C_9H_7NO$	+145.0527	1001
8	Methionine (M)	$+C_9H_8N$	+130.0652	975
9	Methionine (M)	$+C_8H_5NO$	+131.0371	933
10	Methionine (M)	$+C_8H_5NO_2$	+147.0321	879
11	Phenylalanine (F)	$+C_9H_9N$	+131.0735	847
12	Tryptophan (W)	$+C_9H_7NO$	+145.0527	841
13	Methionine (M)	$+C_9H_7N$	+129.0579	737
14	Methionine (M)	$+C_9H_7NO$	+145.0528	680
15	cysteine (C)	$+C_9H_7NO_3$	+177.0456	540
16	cysteine (C)	$+C_9H_7N$	+129.0579	518
17	cysteine (C)	$+C_9H_7NO_2$	+161.0477	512
18	cysteine (C)	$+C_9H_9N$	+131.0735	503
19	cysteine (C)	$+C_9H_9NO$	+147.0684	498
20	cysteine (C)	$+C_9H_7NO$	+145.0528	473
21	Tyrosine (Y)	$+C_9H_9NO$	+147.0687	183
22	Tyrosine (Y)	$+C_9H_7N$	+129.0578	119
23	Tyrosine (Y)	$+C_9H_9N$	+131.0735	117

Table S3. Summary of calculated GRAVY of labelled peptide and full length of WT-DHFR. (**refers to Figure 4J**)

number	labelled site	labelled peptide	GRAVY
1	M1	MISLI	2.78
2	R12	LAVDRVIGM	1.333
3	M16	RVIGMENAM	0.267
4	M20	MENAMPWNL	-0.4
5	P21	ENAMPWNLP	-0.2
6	W22	NAMPWNLPA	-0.2
7	P25	PWNLPADLA	0.089
8	W30	ADLAWFKRN	-0.678
9	F31	DLAWFKRNT	-0.956
10	K32	LAWFKRNTL	-0.144
11	R33	AWFKRNTLN	-0.956
12	K38	NTLNKPVIM	0.133
13	P39	TLNKPVIMG	0.478
14	M42	KPVIMGRHT	-0.411
15	R44	VIMGRHTWE	-0.289
16	H45	IMGRHTWES	-0.844
17	W47	GRHTWESIG	-1.1
18	R52	ESIGRPLPG	-0.5
19	P53	SIGRPLPGR	-0.611
20	P55	GRPLPGRKN	-1.844
21	R57	PLPGRKNII	-0.3
22	K58	PLPGRKNIILS	0.3
23	P66	SSQPGTDDV	-1.222
23	R71	GTDDRVTW	-0.644
25	W74	DRVTWVKSV	-0.343
26	K76	RVTWVKSVDE	-0.078
27	C85	IAACGDV	1.557
28	P89	CGDVPEIMV	1.233
29	M92	VPEIMVIGG	1.489
30	R98	IGGGRVYEQFL	0.118
31	Y100	GGRVYEQFL	-0.589
32	F103	VYEQFLP KA	-0.133
33	P105	EQFLPKAQK	-1.278
34	K106	QFLPKAQKL	-0.467
35	K109	PKAQKLYLT	-0.611
**average**			**-0.14**

		full length	-0.29

Table S4. Summary of calculated GRAVY of labelled peptide and full length of Tau-K18. (**refers to Figure 4J**)

number	labelled site	labelled peptide	GRAVY
1	P5	LQTAPVPMP	0.3
2	P7	TAPVPMPDL	0.3
3	M8	APVPMPDLK	-0.056
4	P9	APVPMPDLKNV	-0.644
5	K12	MDPLKNVKS	-0.811
6	K15	LKNVKSKIG	0.422
7	K17	KSKIGST	-0.157
8	K25	TENLKHQPG	-1.609
9	H26	ENLKHQPGG	-1.68
10	P28	LKHQPGGGK	-1.12
11	K32	PGGGKVQII	0.333
12	K38	QIINKKLDL	-0.286
13	K39	IINKKLDLS	0.111
14	K48	NVQSKCGSK	-0.386
15	C49	VQSKCGSKD	-1.122
16	K52	KCGSKDNIK	-1.433
17	K56	KDNIKHVPG	-0.243
18	H57	DNIKHVPGG	-0.867
19	P59	IKHVPGGGS	-0.222
20	Y68	VQIVYKPVD	0.367
21	K69	QIVYKPVDL	0.367
22	P70	IVYKPVDLS	0.622
23	K75	VDLSKVTSK	-0.156
23	K79	KVTSKCGSL	0
25	C80	VTSKCGSLG	0.389
26	H87	LGNIHHKPG	-0.791
27	H88	GNIHHKPGG	-0.791
28	K89	NIHHKPGGG	-1.17
29	P90	HHKPGGGQ	-1.17
30	K98	QVEVKSEKL	-0.767
31	K101	VKSEKLDFK	-0.967
32	F104	EKLDFKDRV	-1.333
33	K111	RVQSKIGSL	-0.156

34	H120	DNITVPGG	-0.511
35	P122	ITHVPGGGN	-0.167
36	K127	PGGGNKKIE	-0.944
37	K128	PGGGNKKIE	-0.944
**average**			**-0.47**
		**full length**	**-0.63**

Table S5. Summary of calculated GRAVY of labelled peptide and full length of WT-TTR. (**refers to Figure 4J**)

number	labelled site	labelled peptide	GRAVY
1	M1	MASHRLLLLC	1.29
2	H4	MASHRLLLLC	1.29
3	R5	MASHRLLLLC	1.29
4	C10	LLLLCLAGL	2.967
5	F16	AGLVFVSEA	1.544
6	P22	SEAGPTGTG	-0.744
7	K29	TGESKCPLM	-0.3
8	C30	GESKCPLMV	0.244
9	P31	ESKCPLMVK	-0.144
10	M33	KCPLMVKVL	1.222
11	K35	PLMVKVLDA	1.189
12	R41	LDAVRGSPA	0.089
13	P44	VRGSPAINV	0.433
14	H51	NVAVHVFR	0.233
15	F53	AVHVFRKAA	0.556
16	R54	VHVFRKAAD	-0.033
17	K55	HVFRKAADD	-0.889
18	W61	ADDTWEPFA	-0.811
19	F64	TWEPFASGKT	-0.8
20	K68	FASGKTSES	-0.7
21	H76	SGELHGLTT	0.467
22	F84	TEEEFVEGI	-0.4
23	Y89	VEGIYKVEI	0.533
23	K90	EGIYKVEID	-0.322
25	K96	EIDTKSYWK	-1.556
26	Y98	DTKSYWKAL	-1.044
27	W99	TKSYWKALG	-0.7
28	K100	KSYWKALGI	-0.122

29	P106	LGISPFHEH	-0.178
30	F107	GISPFHEHA	-0.4
31	H108	ISPFHEHE	-0.744
32	H110	PFHEHAEVV	-0.222
33	F115	AEVVFTAND	0.4
34	P122	NDSGPRRYT	-1.031
35	R123	DSGPRRYTI	-0.838
36	R124	SGPRRYTIA	-0.685
37	Y125	GPRRYTIAA	-0.123
38	P133	ALLSPYSYS	0.033
39	Y134	LLSPYSYST	0.064
40	Y136	SPYSYSTTA	0.754
41	P145	VVTNPK	0.52
42	K146	VTNPKE	-0.217
**average**			**0.05**
		**full length**	**-0.03**

Notes: The calculated peptides contained 9 amino acids. The grand average of hydropathicity (GRAVY) was calculated using Expasy Protparam.

Table S6. 61 proteins in common identified by AggID and traditional fractionation method. (refers to **Figure S45**)

number	protein	function
1	GADD45B	stress response
2	PTPN14	stress response
3	DNAJB6	chaperone
4	POMP	chaperone
5	HSPA1L	chaperone
6	DNAJB9	chaperone
7	HSPH1	chaperone
8	7-Mar	degradation
9	TRIM11	degradation
10	ZFAND5	degradation
11	C16orf72	degradation
12	TRPC4AP	degradation
13	BAG3	degradation
14	MKLN1	degradation
15	ATG101	degradation
16	PLK2	degradation
17	SQSTM1	degradation
18	RLIM	degradation
19	PPTC7	degradation
20	AZIN1	degradation
21	CDC6	DNA/RNA processing
22	CLSPN	DNA/RNA processing
23	ID3	DNA/RNA processing
24	ZNF160	DNA/RNA processing
25	TSPYL2	DNA/RNA processing
26	GMNN	DNA/RNA processing
27	IFRD1	DNA/RNA processing
28	SIRT7	DNA/RNA processing
29	ZC3H3	DNA/RNA processing
30	ZNF503	DNA/RNA processing
31	ETV3	DNA/RNA processing
32	PTOV1	DNA/RNA processing
33	TIPARP	DNA/RNA processing
34	ZNF3	DNA/RNA processing
35	TRAIP	DNA/RNA processing
36	PMF1	DNA/RNA processing

37	PIAS3	DNA/RNA processing
38	GGNBP2	DNA/RNA processing
39	ZNF622	DNA/RNA processing
40	SUPT4H1	DNA/RNA processing
41	RASIP1	synthesis
42	PPP1R15A	synthesis
43	ALAS1	synthesis
44	DUSP2	synthesis
45	JMJD1C	synthesis
46	ABHD4	synthesis
47	PTGS2	synthesis
48	PLA2G16	trafficking
49	BLZF1	trafficking
50	IFT52	trafficking
51	ZFAND2A	others
52	IP6K2	others
53	OSGIN1	others
54	MRFAP1	others
55	ZMYND19	others
56	CCDC82	others
57	OSER1	others
58	BFAR	others
59	SIVA1	others
60	ASPHD1	others
61	MICB	others

Table S7. Photo induced proximity labeling proteins related to proteostasis and non-proteostasis network. (refers to **Figure 6C**)

Stress response	trafficking	chaperone	degradation	DNA/RNA processing	synthesis	others
ANKRD1	APOL1	ABHD3	ABHD2	ARC	ABHD4	ARRDC2
ATF3	APOL6	ASB8	AZIN1	BHLHE40	ALAS1	BCAS4
CFLAR	BLZF1	BTRC	FBXL5	BRF2	AMD1	CCDC103
GADD45B	KDELR3	CLU	FBXO32	CCNA1	ARAP2	CCDC15
HOXC11	MOAP1	DNAJB1	HERPUD1	CCND3	ATP5G1	CCDC82
HSPA1B	PINK1	DNAJB4	KCTD13	CDC6	ATP5G2	CYB5D1
MCL1	PLEKHB2	HSPA1L	KLHL15	CDKN1A	ATP5G3	DDX53
PTPN14	PRELID1	HSPA6	KLHL21	CDT1	DNAH14	DEF8
SESN2	TNKS2	HSPB8	KLHL24	CNOT8	DUSP5	EGFLAM
STYXL1	UHMK1	NECAB3	KLHL25	CPEB1	DYRK3	GRIN2D
TRIB3	AMOTL2	POMP	KLHL28	DGAT2	EIF2AK1	IFFO2
C10orf35	ANKH	TMBIM6	7-Mar	DGCR6L	PPP1R15A	LONRF3
CSF1	ARL6IP1	BAG3	OLR1	DUSP1	PPP1R15B	MORC4
CSRP2	ATP6V1H	BBS10	PCMTD1	DUX1	ASH1L	NRIP3
EIF1AD	B9D1	CHAC1	PRAME	E2F3	BTG1	PHTF2
GDF15	BBS4	DNAJA1	PTPRN	EPAS1	C14orf80	PNMA1
HMGCR	BBS5	DNAJB2	RNF145	ETS1	CCSER2	RIT1
HSPBAP1	BVES	DNAJB6	RNF4	ETS2	CEP44	RND3
IL17RA	EFCAB7	DNAJB9	SOCS3	GEN1	CEP57L1	TUFT1
NLRP1	FAM46A	FNIP1	TNKS	GPATCH3	CHKA	ZFAND2A
RHPN2	FAM58A	GBP3	TRIM35	HDAC11	DUSP10	ZSWIM4
	GOLT1B	HSPB1	TRPC4AP	HIF1A	DUSP14	A0A6I8PU40
	GUF1	HSPH1	WSB1	ID1	DUSP2	ABTB2
	IFT52	15-Sep	ZFAND5	IER2	ETNK1	ADIPOR2
	MAPK8IP3	SERPINB7	AMBRA1	IER5	FAM83G	ANKRD18A
	NFKBIB	SPTY2D1	ANKRD13B	IFIT2	FDFT1	ANKRD39
	PARP8	SYNM	ARRDC4	IFRD1	FIGN	ARL5B
	PDE4D	HIPK1	ASB6	IL3RA	GNB5	ARMCX5
	PICK1	NUAK1	ASXL1	JUN	JMJD1C	ASPHD1
	PLA2G16	PIM1	ATG101	KRBOX4	JMJD4	ASPSCR1
	PNPLA2	PIM2	BCKDHB	MAFK	LMTK2	BFAR
	PNPLA3	SIK1	C16orf72	MED10	LPIN3	BOP
	RAB39A	CDK6	CCNB1IP1	NBPF1	MAD2L1BP	C12orf4
	RASSF9		CCRN4L	NFE2L1	MAPK6	C15orf39
	RHOBTB3		CDKN2A.1	NR1D1	METTL22	C16orf87

RHOD	DCAF12	OAS1	MYO10	C17orf51
SGK1	FAM83D	OASL	NAA60	C1orf198
SNF8	FANCL	RBM48	NDUFS4	C2orf42
SNX10	FBXO44	RGAG4	NEK2	C5orf34
SNX14	FBXW11	RHEBL1	NMNAT2	C6orf62
SNX15	FBXW5	TRMT44	NSUN3	CCDC138
SPHK1	FEM1C	ZFP37	OSGIN2	CCDC28A
TGOLN2	GNL3L	ZNF195	PANK3	CCDC71L
UGCG	HERC6	ZNF200	PAQR5	CCNJL
WHAMM	KCTD10	ZNF317	PDK4	CENPO
ZBED5	KCTD5	ABLIM3	PHLDA2	CEP104
	KLHDC10	AHR	PLIN2	CEP120
	MAGEA3	ALKBH1	PPP2R3C	CEP95
	MAGEA6	AP5Z1	PTGS2	CHIC1
	6-Mar	APEX2	RASIP1	CNKSR3
	MKLN1	ARMC7	RELT	CXorf40B
	NDFIP2	ATF6	RGS2	CYP26B1
	NKIRAS1	ATXN1	RNF10	DIAPH2
	OS9	BAHD1	RNF168	DONSON
	OTUD1	BMP6	RNF19B	EID2
	OTUD5	CBX6	SENP5	FAAH2
	PFKFB3	CCDC130	SLC25A25	FAM83A
	PJA1	CCDC59	SLC25A38	FOSL1
	PJA2	CCNC	SLC26A11	FRMD6
	PLK2	CCND1	SLC37A4	FRMD8
	PPTC7	CCNF	SLC9A7	GCH1
	RFWD2	CENPT	SMCR8	GPATCH1
	RFWD3	CLSPN	SPICE1	GPATCH2L
	RLIM	CYP1B1	SQLE	GRID2
	RMND5B	CYR61	SSR2	HHAT
	RPS27A	DUSP16	STIL	HMGXB3
	SMURF1	EAF1	TBC1D30	IFITM10
	SQSTM1	ELOF1	TMEM170A	IL7R
	TNFAIP1	ENC1	WEE1	IP6K2
	TRIM11	ETV3	ABHD4	ITPRIPL2
	TRIM37	FAM127B	ALAS1	JPH1
	TTLL4	FANCC	AMD1	KCNG3
	UBR3	GATB	ARAP2	KCTD2

USP16	GGNBP2	ATP5G1	KRBA1
USP18	GMNN	ATP5G2	LAMA1
USP37	GNAS	ATP5G3	LDOC1L
XDH	HIST1H2AC	DNAH14	LRRC42
YOD1	HOXC13	DUSP5	LRRC58
YPEL5	ID3	DYRK3	LRRC75A
ZFAND6	IFIH1	EIF2AK1	MAGEA12
ZRANB1	IFIT1	PPP1R15A	MAPKBP1
ZUFSP	ING1	PPP1R15B	MBIP
ZYG11A	IRAK2	ASH1L	MBOAT2
CTNNB1	ISG20	BTG1	MCOLN3
	JMY	C14orf80	MFAP3
	KANSL1	CCSER2	MICB
	LRIF1	CEP44	MORN1
	MAML1	CEP57L1	MRFAP1
	MAP1LC3B2	CHKA	MRPL33
	MCM10	DUSP10	MZT2A
	MED19	DUSP14	NAV2
	MGA	DUSP2	NOTCH1
	MSX1	ETNK1	NT5C1A
	NABP1	FAM83G	OFD1
	NCOA4	FDFT1	OSER1
	NOVA2	FIGN	OSGIN1
	NR4A2	GNB5	PCNXL4
	NRIP1	JMJD1C	PLCXD1
	PEG10	JMJD4	PLCXD2
	PER3	LMTK2	PRADC1
	PHF20	LPIN3	PRICKLE3
	PHLDA3	MAD2L1BP	RGPD5
	PIAS3	MAPK6	SARAF
	PMF1	METTL22	SIVA1
	POC1A	MYO10	SLMO2
	POLH	NAA60	SMIM11
	POLR3K	NDUFS4	SPAG4
	PPM1D	NEK2	SUPT7L
	PRDM6	NMNAT2	SYDE2
	PTOV1	NSUN3	TM2D2
	RBM5	OSGIN2	TNFSF9

RBPMS	PANK3	TSPYL1
SCNM1	PAQR5	TTC14
SIRT7	PDK4	TUBD1
SIX4	PHLDA2	UBL5
SNAPC1	PLIN2	UBXN2A
SNAPC3	PPP2R3C	ULBP3
SPATC1L	PTGS2	ZMYND19
SREBF2	RASIP1	ZSWIM3
STAU1	RELT	ZSWIM6
STON2	RGS2	JAK1
SUPT4H1	RNF10	
TAF7	RNF168	
TBX2	RNF19B	
TDP2	SENP5	
TDRD12	SLC25A25	
TDRD9	SLC25A38	
TEN1	SLC26A11	
TERF2IP	SLC37A4	
TFDP2	SLC9A7	
THAP5	SMCR8	
TIPARP	SPICE1	
TNIP2	SQLE	
TPGS2	SSR2	
TRAIP	STIL	
TSC22D1	TBC1D30	
TSPYL2	TMEM170A	
UBB	WEE1	
USB1		
VDR		
ZBTB11		
ZBTB20		
ZC3H3		
ZFP36L1		
ZFX		
ZNF121		
ZNF131		
ZNF14		
ZNF160		

ZNF260
ZNF292
ZNF3
ZNF304
ZNF35
ZNF410
ZNF417
ZNF419
ZNF426
ZNF503
ZNF562
ZNF600
ZNF622
ZNF7
ZNF724P
ZNF777
ZNF791
ZNF81
ZNF813
ZNF845
ZNHIT6
ZSCAN26
ZXDB
ZXDC

Table S8. Summary of the functions of proteins with fold change > 4, P-value < 0.01 in volcano plot. (refers to **Figure 6B**)

number	protein	function
1	HSPA1L	chaperone
2	HSPA6	chaperone
3	NECAB3	chaperone
4	STYXL1	chaperone
5	HERPUD1	degradation
6	KLHL25	degradation
7	MOAP1	degradation
8	PCMTD1	degradation
9	TNKS2	degradation
10	WSB1	degradation
11	PJA1	degradation
12	PLK2	degradation
13	RLIM	degradation
14	CDC6	DNA/RNA processing
15	CDKN1A	DNA/RNA processing
16	EPAS1	DNA/RNA processing
17	HIF1A	DNA/RNA processing
18	IER5	DNA/RNA processing
19	RBM48	DNA/RNA processing
20	ZFP37	DNA/RNA processing
21	ANKRD1	stress response
22	TRIB3	stress response
23	HMGCR	stress response
24	ATP5G3	synthesis
25	PPP1R15A	synthesis
26	ANKRD42	not defined
27	ARRDC2	not defined
28	NRIP3	not defined
29	ZFAND2A	not defined
30	ADIPOR2	not defined
31	LPPC58	not defined
32	TIPARP	not defined

Notes: Proteins termed as not defined were those with no reported functions at UniProt or are not related to protein homeostasis.

Table S9. Summary of spectrum counts on labeling sites and corresponding SASA values of HSPA1B (HSP70). (refers to **Figure 6D**)

number	labeling sites	spectrum counts (-Bortezomib)	spectrum counts (+Bortezomib)	SASA	flip-out residues labeling
1	Y15	1	3	58.44	YES
2	C17	4	22	2.16	YES
3	K25	3	19	108.83	NO
4	K77	0	1	80.48	YES
5	F78	0	3	36.91	YES
6	P81	1	8	109.28	YES
7	M87	1	19	51.42	YES
8	K88	1	37	160.35	NO
9	H89	0	22	65.9	YES
10	W90	0	17	20.72	YES

Notes: The solvent accessible surface areas (SASA) values were calculated using NACCESS program.

3. Synthesis and Characterizations

Synthesis of **P8**:

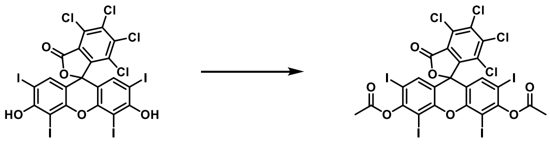

Scheme S1. Synthetic procedure of **P8**.

Synthesis of **P8** refers to the previous published work.[17] To a suspension of Rose Bengal (P5, 0.5 mmol) in DCM (10 mL), 0.05 mmol 4-dimethylaminopyridine and 1.1 mmol pyridine were added. Next, 1.1 mmol acyl chloride was added dropwise and the resulting solution was reacted for 4 h at room temperature. The obtained solution was washed with water and saturated aqueous NH_4Cl before concentrated under reduced pressure. The final red solid product was obtained after column chromatography on silica gel (PE/EA=1/1, v/v). Yield: 481.8 mg (91.3%); ^{1}H NMR (700 MHz, DMSO-d_6) δ 7.85 (s, 2H), δ 2.44 (s, 6H); ^{13}C NMR (176 MHz, DMSO-d_6) δ 170.8, 167.4, 163.4, 154.7, 152.1, 139.4, 137.3, 136.1, 132.1, 127.2, 125.1, 116.6, 87., 84.8, 60.2, 21.7; HRMS (ESI+) *m/z*: [M+H]$^+$ calcd 1056.5276, found 1056.5252.

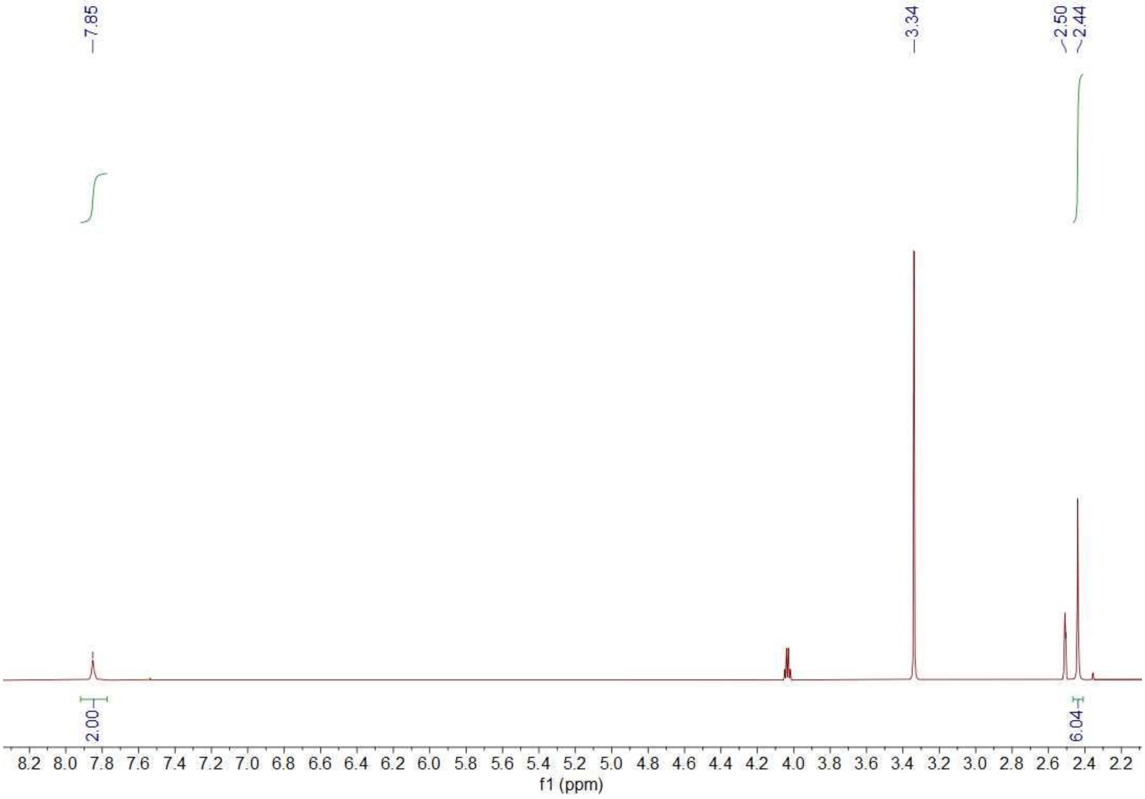

Figure S49. ^{1}H NMR spectrum of **P8**.

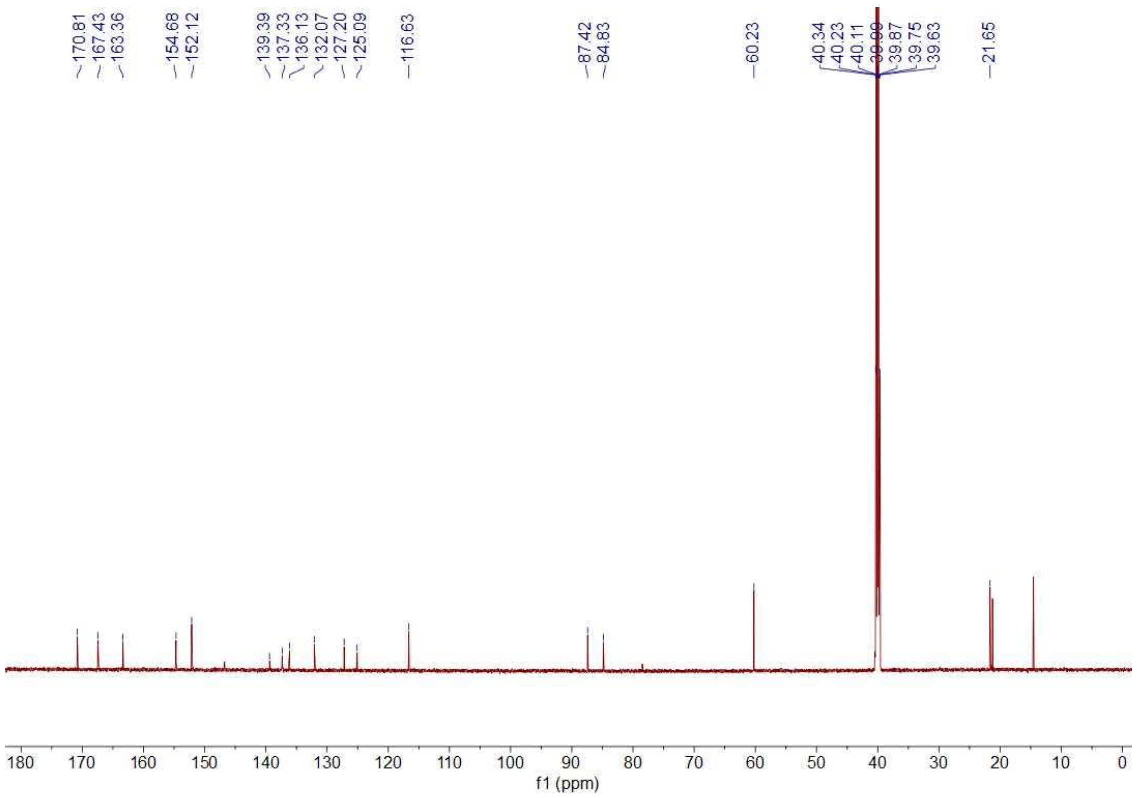

Figure S50. ^{13}C NMR spectrum of **P8**.

4. References

[1] Y. Huang, Y. Bai, W. Jin, D. Shen, H. Lyu, L. Zeng, M. Wang, Y. Liu, *Biochemistry* **2021**, *60*, 2447-2456.
[2] D. W. Baggett, A. Nath, *Biochemistry* **2018**, *57*, 6099-6107.
[3] P. Kumar, A. Nagarajan, P. D. Uchil, *Cold Spring Harbor protocols* **2018**, *6*, 469-417.
[4] S. Okuda, Y. Watanabe, Y. Moriya, S. Kawano, T. Yamamoto, M. Matsumoto, T. Takami, D. Kobayashi, N. Araki, A. C. Yoshizawa, T. Tabata, N. Sugiyama, S. Goto, Y. Ishihama, *Nucleic Acids Res.* **2017**, *45*, D1107-D1111.
[5] E. C. B. Johnson, E. K. Carter, E. B. Dammer, D. M. Duong, E. S. Gerasimov, Y. Liu, J. Liu, R. Betarbet, L. Ping, L. Yin, *Nat. Neurosci.* **2022**, *25*, 213-225.
[6] Y. Liu, Y. Ge, R. Zeng, W. S. C. Ngai, X. Fan, P. R. Chen, *CCS Chem.* **2023**, *5*, 802-813.
[7] J. Ye, S. Bazzi, T. Fritz, K. Tittmann, R. A. Mata, J. Uranga, *Angew. Chem. Int. Ed.* **2023**, e202304163.
[8] L. Grassi, C. Cabrele, *Amino Acids* **2019**, *51*, 1409-1431.
[9] X. R. Liu, M. M. Zhang, B. Zhang, D. L. Rempel, M. L. Gross, *Anal. Chem.* **2019**, *91*, 9238-9245.
[10] J. E. Plowman, S. Deb-Choudhury, A. J. Grosvenor, J. M. Dyer, *Photochem. Photobiol. Sci.* **2013**, *12*, 1960-1967.
[11] R. Kehm, T. Baldensperger, J. Raupbach, A. Höhn, *Redox Biol.* **2021**, *42*, 101901.
[12] P. Di Mascio, G. R. Martinez, S. Miyamoto, G. E. Ronsein, M. H. G. Medeiros, J. Cadet, *Chem. Rev.* **2019**, *119*, 2043-2086.
[13] H. Feng, Q. Zhao, B. Zhang, H. Hu, M. Liu, K. Wu, X. Li, X. Zhang, L. Zhang, Y. Liu, *Angew. Chem. Int. Ed.* **2022**, *62*, e202215215.
[14] a) D. Warde-Farley, S. L. Donaldson, O. Comes, K. Zuberi, R. Badrawi, P. Chao, M. Franz, C. Grouios, F. Kazi, C. T. Lopes, A. Maitland, S. Mostafavi, J. Montojo, Q. Shao, G. Wright, G. D. Bader, Q. Morris, *Nucleic Acids Res.* **2010**, *38*, W214-W220; b) K. Zuberi, M. Franz, H. Rodriguez, J. Montojo, C. T. Lopes, G. D. Bader, Q. Morris, *Nucleic Acids Res.* **2013**, *41*, W115-W122; c) M. Franz, H. Rodriguez, C. Lopes, K. Zuberi, J. Montojo, G. D. Bader, Q. Morris, *Nucleic Acids Res.* **2018**, *46*, W60-W64.
[15] C. Behl, *Trends Pharmacol. Sci.* **2016**, *37*, 672-688.
[16] a) A. B. Meriin, A. Narayanan, L. Meng, I. Alexandrov, X. Varelas, I. I. Cissé, M. Y. Sherman, Proc. Natl. Acad. U. S. A. 2018, 115, E7043-E7052; b) T. A. Colvin, V. L. Gabai, J. Gong, S. K. Calderwood, H. Li, S. Gummuluru, O. N. Matchuk, S. G. Smirnova, N. V. Orlova, I. A. Zamulaeva, Cancer Res. 2014, 74, 4731-4740.
[17] W. Chyan, H. R. Kilgore, B. Gold, R. T. Raines, *J. Org. Chem.* **2017**, *82*, 4297-4304.
